# dStruct: identifying differentially reactive regions from RNA structurome profiling data

**DOI:** 10.1186/s13059-019-1641-3

**Published:** 2019-02-21

**Authors:** Krishna Choudhary, Yu-Hsuan Lai, Elizabeth J. Tran, Sharon Aviran

**Affiliations:** 1 Department of Biomedical Engineering and Genome Center, University of California, Davis, One Shields Avenue, Davis, 95616 CA USA; 2 Department of Biochemistry, Purdue University, BCHM 305, 175 S. University Street, West Lafayette, 47907-2063 IN USA; 3 Purdue University Center for Cancer Research, Purdue University, Hansen Life Sciences Research Building, Room 141, 201 S. University Street, West Lafayette, 47907-2064 IN USA

**Keywords:** RNA structure, Structure probing, Differential analysis, Transcriptome-wide profiling, SHAPE, DMS, PARS

## Abstract

RNA biology is revolutionized by recent developments of diverse high-throughput technologies for transcriptome-wide profiling of molecular RNA structures. RNA structurome profiling data can be used to identify differentially structured regions between groups of samples. Existing methods are limited in scope to specific technologies and/or do not account for biological variation. Here, we present dStruct which is the first broadly applicable method for differential analysis accounting for biological variation in structurome profiling data. dStruct is compatible with diverse profiling technologies, is validated with experimental data and simulations, and outperforms existing methods.

**Electronic supplementary material:**

The online version of this article (10.1186/s13059-019-1641-3) contains supplementary material, which is available to authorized users.

## Background

RNA molecules adopt diverse and intricate structures, which confer on them the capacity to perform key roles in myriads of cellular processes [1, 2]. Structures, and hence functions, of RNAs are modulated by a number of factors, such as solution environment (in vivo or in vitro), presence of RNA-binding proteins or ligands, mutation in the RNA sequence, and temperature [3]. The amalgamation of classic chemical probing methods, which probe RNA structure at nucleotide resolution, with next-generation sequencing has ushered in a new era of RNA structuromics [2, 3]. In fact, recent developments have led to a diversity of structure probing or structure profiling (SP) technologies [4, 5]. These technologies have made it possible to perform comparative analysis of structures of select RNAs or whole RNA structuromes simultaneously [6–21].

SP technologies result in nucleotide-level scores, called as *reactivities*, that summarize one or more aspects of local structure (e.g., steric constraint due to base pairing interaction). To this end, they utilize probing reagents that react with RNA nucleotides in a structure-sensitive manner. The degree of reaction at a nucleotide is a function of local stereochemistry. A number of reagents (e.g., SHAPE, DMS, nucleases) exist, which react with sensitivity to different aspects of local stereochemistry [22–24]. Moreover, depending on the reagent, the reaction results either in chemical modification of the sugar/base moiety or a cleavage of the RNA strand. Its degree is captured in a cDNA library through primer extension by reverse transcriptase, which either stops at modified nucleotides or proceeds but introduces a mutation [3]. In addition, to assess the background noise, most SP technologies use samples that are not treated with reagent [19, 25–28]. Furthermore, there are diverse library preparation methods. For example, some methods enrich for modified transcript copies [7, 29]. Indeed, SP technologies differ in their choices of probing reagents and key library preparation steps. Yet, each technology has its advantages, which might make it the preferred choice for certain studies. Irrespective of the SP technology and end goals of a study, cDNA libraries are sequenced and data is processed to obtain reactivities. Often, this involves combining information from the treated and untreated control samples [19, 30–32]. The sequence of nucleotide reactivities for a transcript is called its *reactivity profile*. It is noteworthy that reactivity profiles are estimated using approaches customized to the SP technology used for a study [6]. Hence, different approaches yield reactivities with different statistical properties [33, 34]. Nonetheless, amid the diversity of protocols and reactivity estimation methods, identifying differentially reactive regions (DRRs) is a common step in the majority of SP studies [6]. In this article, we focus on identifying DRRs from SP data.

Several methods have been developed for differential analysis of SP data [8, 9, 20, 21, 35]. They utilize two common principles. First, they premise that differential structure manifests at a regional level and not at individual isolated nucleotides. Second, they recognize that SP data manifests substantial noise at the nucleotide level. Despite these shared principles, these methods differ in how they account for noise in the data. deltaSHAPE [8] and StrucDiff [9] address nucleotide-level noise by smoothing reactivity or count profiles. Subsequently, they find DRRs from smoothed profiles. Note that we call the method developed by Wan et al. StrucDiff after the name of the score it employs. In contrast, the method included in the PARCEL pipeline assesses the significance of changes in counts at the nucleotide level first. It considers nucleotides as “genes” in an RNA-seq model. To this end, it uses edgeR [36] to account for nucleotide-level noise and compute *p* values for changes in counts. Next, it chains together nucleotides with significant changes as DRRs by performing a second statistical test [21] (henceforth, we refer to this method as PARCEL). Similarly, Mizrahi et al. account for nucleotide-level noise with a two-step “regression and spatial analysis” approach [20] (for convenience, we acronymize this method as RASA). Specifically, to evaluate the changes in reactivities at the nucleotide level, they use generalized mixed model extension of logistic regression with counts and coverages as inputs. Next, they identify the regions with clusters of differentially reactive nucleotides using a permutation test. Another method, classSNitch, utilizes a machine learning classifier that learns from training data how to distinguish between nucleotide-level noise and DRRs [35].

Despite notable developments in existing methods, several key challenges remain unaddressed. First, it is known that different regions in RNAs manifest different levels of variation among biological replicates (henceforth, called biological variation) [30, 37–40]. Hence, inherently variable regions should be distinguished from DRRs. Indeed, DRRs are expected to differ consistently between the two groups of samples distinguished by a structure-altering factor. Furthermore, between-group variation in DRRs should significantly exceed the variation between samples of the same group. However, deltaSHAPE, StrucDiff,and classSNitch do not account for biological variation. While PARCEL and RASA account for biological variation, they are limited in scope to specific technologies. One issue that underlies this limitation is that they do not utilize untreated samples. Yet, untreated samples are an integral component of most SP technologies [6, 7, 19, 25–28]. Importantly, combining information from both treated and untreated samples has been shown to provide accurate assessment of reactivities [30–32]. Furthermore, broadly applicable approaches for estimating reactivities combine information from the two kinds of samples and yield analog reactivity values [30, 31]. As PARCEL and RASA are based on counts, they are not readily applicable to analog reactivity readouts. Besides this, PARCEL does not account for coverage variations within a transcript. Second, in many studies, candidate regions, which might be DRRs, are not known a priori [8, 11, 18]. Hence, they need to be constructed de novo. However, StrucDiff and classSNitch require predefined regions, which are typically obtained from a collateral study. For example, a collateral study might indicate sites with single-nucleotide variants between two cell lines, and candidate regions might be constructed as short stretches of nucleotides flanking each variant [9, 17]. Third, searching for DRRs in transcriptome-wide data might involve testing multiple hypotheses. While each hypothesis considers the same question about the presence/absence of a differential signal, a separate test might be conducted for each candidate region of each candidate RNA. This leads to the so-called multiple testing problem [41]. Since no hypothesis test is perfect, there is a risk of false positive from each test. When we test numerous hypotheses on a dataset simultaneously, the associated risk of false-positive results grows. Hence, it is recommended that *p* values (or alternative summaries of statistical significance) assessed from each test be adjusted to control for the risk of false discoveries. However, deltaSHAPE, classSNitch, and RASA do not perform multiple testing correction. Fourth, if candidate regions were known a priori, restricting search of DRRs to the predefined candidates before statistical testing might improve power in the context of multiple testing [41]. We call this scenario *guideddiscovery*. However, deltaSHAPE, PARCEL, and RASA allow for comparison with a priori knowledge only after de novo discovery of DRRs. Fifth, of significance in SP data is the “pattern” of reactivities in a region [33, 35, 39, 42–45]. Specifically, in a DRR, while some nucleotides could become more reactive, others could become less reactive, thereby keeping the average level insignificantly altered while altering the reactivity pattern in that region [10, 46]. For example, this could be indicative of a hairpin transitioning to a G-quadruplex [47]. Indeed, in a study assessing how experts classify the differences in reactivity profiles by visual inspection, reactivity pattern was found to be key to human decision [35]. However, none of the methods except for classSNitch explicitly account for reactivity patterns. While classSNitch accounts for reactivity patterns, it utilizes a classifier trained with SHAPE data only. Hence, it is limited in scope to SHAPE data. Finally, the need to account for reactivity patterns limits the applicability of differential analysis methods commonly used in other genomic disciplines (e.g., differential methylation analysis from bisulfite sequencing data). These methods generally seek regional changes in the signal’s magnitude and not the signal’s pattern [48, 49]. Yet, it has been demonstrated that specialized methods accounting for signal patterns in ChIP-seq and bisulfite sequencing data can improve power to detect differential regions [50, 51].

**Fig. 1:**
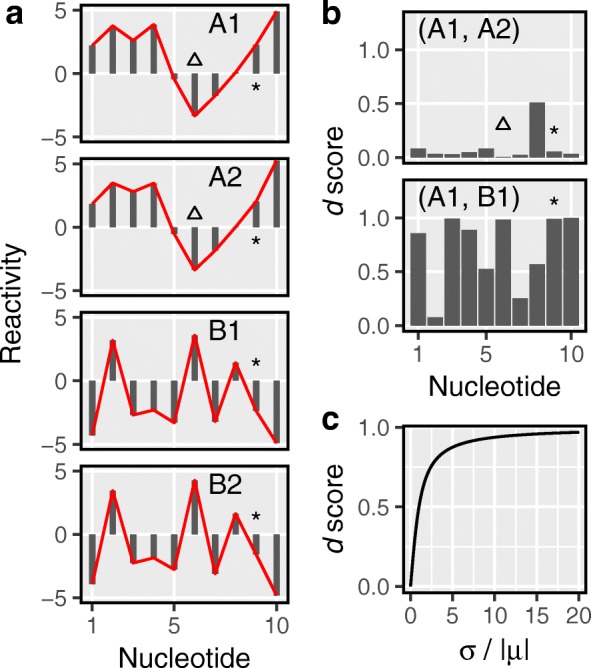
The *d* score quantifies the dissimilarity between reactivities. **a** Four hypothetical reactivity profiles, labeled A1 and A2 (group A) and B1 and B2 (group B). Red lines highlight the reactivity patterns. Triangles mark a nucleotide that maintains identical reactivities within groups. Asterisks mark a nucleotide that flips its reactivity between groups. **b** Comparison of samples from the same group (e.g., A1, A2) results in *d* scores lower than those from between-group comparisons (e.g., A1, B1). A triangle highlights the low *d* score of a nucleotide with high within-group agreement. An asterisk highlights a nucleotide that displays high within-group agreement and therefore results in a low within-group *d* score. It also displays poor between-group agreement, which results in a high between-group *d* score. **c** The *d* score monotonically increases with the absolute value of coefficient of variation

**Fig. 2:**
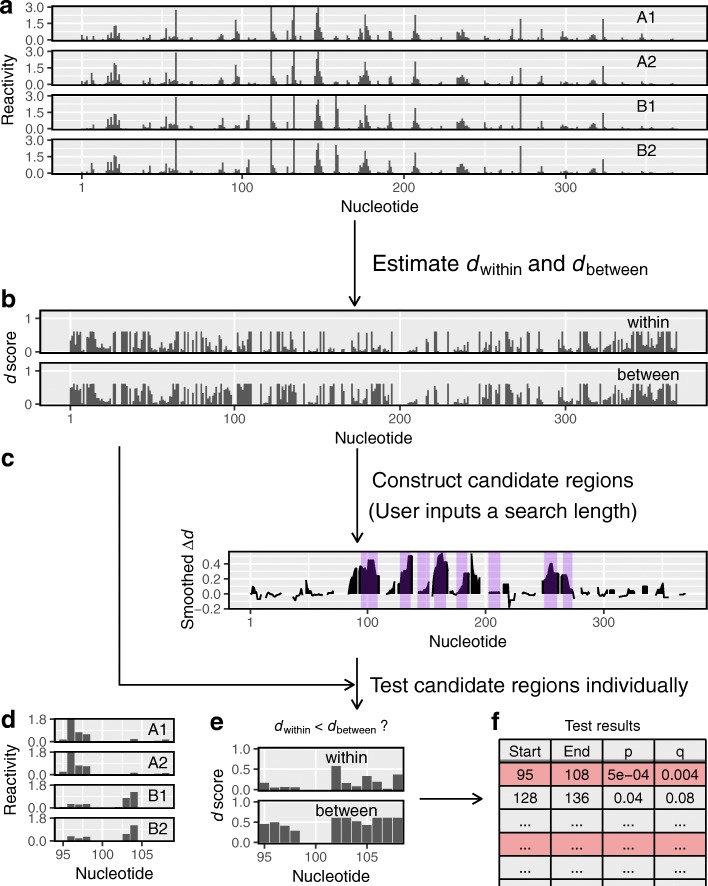
dStruct identifies differentially reactive regions. **a** Users input samples of reactivity profiles, some from group A and some from group B. **b** In the first step, dStruct quantifies the within-group and between-group variations in terms of *d* scores. **c** In the second step, dStruct identifies regions where the between-group variation appears to be greater than the within-group variation. These are highlighted by purple background. This step is skipped if users provide a list of candidate regions. **d** Reactivity profiles for one of the candidate regions. **e** In the third step, dStruct compares the *d*_within_ and *d*_between_ profiles using a Wilcoxon signed-rank test. **f** The results are output as a list of region identifiers, such as the start and end locations of the candidates tested, and the *p* values and *q* values for each region

To address the aforementioned limitations of existing methods, we present dStruct, which identifies DRRs from SP data within a single RNA or a transcriptome. Central to dStruct is a dissimilarity measure, called *d* score. dStruct starts by assessing within-group and between-group variations in reactivities, in terms of nucleotide *d* scores (Figs. 1 and 2a, b). Due to the effect of structure-altering factors in DRRs, the between-group variation is expected to be higher than the within-group variation (Fig. 1b). Hence, next, dStruct screens for regions with evidence of increased *d* scores between groups. This step is skipped if a predefined set of candidate regions is available. Finally, dStruct compares the within-group nucleotide *d* scores with the between-group scores using Wilcoxon signed-rank test and controls the FDR using the Benjamini-Hochberg procedure [52, 53]. dStruct is the first differential analysis method that both directly accounts for biological variation and is applicable to diverse SP protocols. We validated dStruct with data from different SP technologies, namely, SHAPE-Seq, Structure-Seq, and PARS, as well as with simulations. Test datasets vary in size from single RNAs to transcriptomes and feature samples from bacteria, virus, fungi, and humans. In addition, the structure-altering factors include protein binding, ligand binding, and single-nucleotide variants. Besides utilizing real data, we developed a novel approach to simulate biological replicates of SP data. In particular, existing approaches do not provide a way to generate correlated biological replicates [54, 55]. We addressed this gap to allow for proper assessment of dStruct’s performance. dStruct enables guided as well as de novo discovery. In all tests, we demonstrate that for a properly controlled FDR, dStruct has a higher power than existing methods. Besides validations, we discuss in detail the limitations of dStruct as well as of existing approaches.

## Results

### Dissimilarity measure

We define a dissimilarity measure for reactivities, which we call a *d* score. Given a transcript of length *n* and a set of *m* reactivity profiles for the transcript, let *r*_*ij*_ represents the reactivity of nucleotide *i* in profile *j*. If *σ*_*i*_ and *μ*_*i*_ represent the sample standard deviation and mean of reactivities for nucleotide *i*, respectively, then the *d* score of nucleotide *i* is defined as:

1 $$\begin{array}{@{}rcl@{}} d_{i} & = & \frac{2}{\pi} \arctan \left(\frac{\sigma_{i}}{\left| \mu_{i} \right|} \right). \end{array} $$

For *m*=2, the above expression simplifies to:

2 $$\begin{array}{@{}rcl@{}} d_{i} & = & \frac{2}{\pi} \arctan \left(\sqrt{2}\left|\frac{r_{i1} - r_{i2} }{ r_{i1} + r_{i2} }\right| \right). \end{array} $$

Taking the ratio of *σ*_*i*_ and *μ*_*i*_ accounts for the fact that higher reactivities tend to manifest higher fluctuations [56]. However, the ratio by itself is very sensitive to small changes in *μ*_*i*_, especially when *μ*_*i*_ is small. For example, PARS reactivities can be both positive and negative (Fig. 1a). This can result in *μ*_*i*_ being close to 0, while *σ*_*i*_ remains high (e.g., nucleotide highlighted with asterisks in Fig. 1a). Importantly, *σ*_*i*_/|*μ*_*i*_| increases very fast and approaches infinity as *μ*_*i*_ decreases and approaches zero (Additional file 1: Figure S1). However, the dStruct pipeline involves taking means of *d*_*i*_ as we describe below. Since the mean is not robust to outliers, extremely high values of *σ*_*i*_/|*μ*_*i*_| could pose problems in the dStruct pipeline. Hence, we reduce the sensitivity of *σ*_*i*_/|*μ*_*i*_| to changes in *μ*_*i*_ by transforming it with the arctan function. While *σ*_*i*_/|*μ*_*i*_| is unbounded, its arctan-transformed value is bounded between 0 and *π*/2. To restrict its range to [0, 1], we scale it by 2/*π* (Fig. 1b). *d*_*i*_ is 0 when the same reactivity is observed for nucleotide *i* in all samples (e.g., nucleotide highlighted with triangles in Fig. 1a, b). It monotonically increases as *r*_*ij*_ becomes more dispersed (Fig. 1c; see the “Methods” section for details).

### Differentially reactive regions

Equipped with *d* score as a dissimilarity measure, we have developed a method to identify DRRs. Our method has three steps (Fig. 2). First, we assess the within-group and between-group variations in terms of *d* scores. Next, we distinguish between de novo and guided discovery situations. To discover DRRs de novo, we need to identify regions that are potential candidates for DRRs. This is done in the second step by screening for regions where between-group variation appears to be higher, on average, than within-group variation. Note that this step is skipped for guided discovery as candidate regions are predefined from a collateral study. In the third step, to assess the statistical significance at each candidate region, the variation between groups in that region is compared to the variation within groups. If the between-group variation is found to be significantly higher, the region is reported as a DRR. In what follows, we briefly describe each step.

#### Step 1

Given *m*_*A*_ and *m*_*B*_ samples from groups A and B, respectively, let *m*= max(*m*_*A*_,*m*_*B*_) (Fig. 2a). We construct all possible subsets of the *m*_*A*_+*m*_*B*_ samples, such that each subset has *m* samples. Of these subsets, at maximum, two will be homogeneous, i.e., they will comprise of samples from A only or B only. If *m*_*A*_≠*m*_*B*_, there will be only one homogeneous subset with samples from group A if *m*_*A*_>*m*_*B*_ or with samples from group B if *m*_*A*_<*m*_*B*_. All other subsets will be heterogeneous. For each subset, we assess the *d* score for each nucleotide as previously described “Dissimilarity measure”. We use the nucleotide-wise average of *d* scores across homogeneous subsets as the measure of within-group variation, denoted as *d*_within_ (Fig. 2b). Similarly, the average of *d* scores across heterogeneous subsets is used as the measure of between-group variation, denoted as *d*_between_.

#### Step 2

The second step is performed only for de novo discovery, as it constructs candidates for DRRs. In the absence of prior knowledge of where DRRs start and end, we rely on the evidence in the data to construct the so-called data-driven regions [49]. In our case, the evidence is in the difference between *d*_between_ and *d*_within_. Hence, we define *Δd*=*d*_between_−*d*_within_. If *Δd* is positive for all nucleotides in a contiguous region of length greater than or equal to a user-specified length, the region is a potential DRR candidate (Fig. 2b, c). However, DRRs could have altered reactivity patterns without necessarily having altered reactivities at all nucleotides. Indeed, in DRRs, some nucleotides may have *Δd*≤0. Hence, we smooth the *Δd* profile prior to screening for candidates (see the “Methods” section). Then, we search for regions that have a positive value of smoothed *Δd* for all nucleotides (highlighted in purple in Fig. 2c). These regions are deemed potential candidates for DRRs. Note that the smoothed *Δd* profile is used only to construct candidate regions. Inputs to the final step are unsmoothed profiles obtained in Step 1.

#### Step 3

The significance of the differential reactivity pattern in a candidate region (see Fig. 2d for an example) is determined by comparing *d*_within_ and *d*_between_ for the region (Fig. 2e). Specifically, we perform Wilcoxon signed-rank test to test the null hypothesis against the one-sided alternative hypothesis that the population mean of *d*_between_−*d*_within_ is positive [52]. For the set of screened regions from all transcripts, the FDR is controlled using the Benjamini-Hochberg procedure to obtain *q* values (i.e., FDR-adjusted *p* values) [53]. Finally, users obtain a list of regions with their corresponding *p* values and *q* values (Fig. 2f). At this point, it is noteworthy that the final step of the statistical testing is performed only for regions that meet a criterion for minimum quality, i.e., if their average *d*_within_ is less than a threshold (see Additional file 1: Figure S2). Henceforth, we call this criterion the *minimum quality threshold*. Keeping the average *d*_within_ below this threshold ensures that samples in the same group have similar profiles in the region of interest, see the “Methods” section for additional details.

### Validation with small datasets

We tested dStruct on three small datasets for which prior knowledge of DRRs is available from independent sources. In addition, we compared its performance to that of RASA, PARCEL, and deltaSHAPE. Overall, we found that dStruct discovers DRRs de novo while having a minimal false-positive rate. Note that we defer comparison with StrucDiff until the section on large datasets, as the small datasets considered below do not have predefined candidates for DRRs. We require SHAPE data with both predefined candidates and replicate samples to compare dStruct and classSNitch. This is because classSNitch is currently trained for guided discovery in SHAPE data only and dStruct requires replicates. Since data satisfying requirements of classSNitch and dStruct simultaneously is not available, we have excluded classSNitch from performance comparisons.

#### dStruct accurately rejects transcripts with no DRRs

We obtained three replicate samples of four *Saccharomyces cerevisiae* rRNAs (5S, 5.8S, 18S, and 25S) from in vivo DMS probing using the Structure-Seq protocol (see the “Methods” section). Since we probed the samples under identical conditions, there should not be any DRRs between replicated profiles of the same rRNA. To assess the specificities of dStruct and the other methods, for each RNA, we performed null comparisons of each possible pair of samples (labeled group A) with the single remaining sample (labeled group B). Therefore, we created 12 test cases (3 for each of the 4 rRNAs), in which we searched for DRRs. We tested deltaSHAPE, RASA, and PARCEL with default search parameters. deltaSHAPE and RASA use windows of 5 nt and 50 nt by default, respectively. We tested dStruct for both window lengths. PARCEL does not require predefined window lengths. Furthermore, deltaSHAPE accepts only one sample per group. Hence, for group A, we input reactivities assessed from pooled counts to deltaSHAPE (i.e., we tallied counts and coverages across all samples). We summarized the performances as follows.

**Fig. 3:**
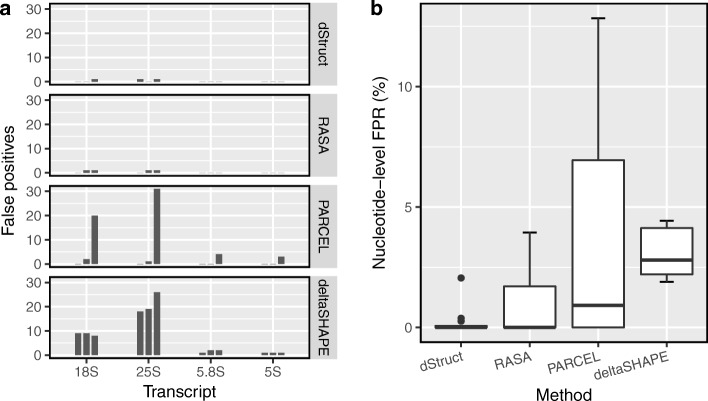
dStruct had a low false-positive rate in null comparisons. In a comparison of biological replicates of rRNAs probed in vivo under identical conditions, **a** dStruct and RASA reported 3 and 4 false positives, respectively. In contrast, PARCEL and deltaSHAPE reported 61 and 97 false positives, respectively. **b** dStruct had lower nucleotide-level false-positive rates than RASA, PARCEL, and deltaSHAPE

We tallied the number of DRRs reported by each method. Out of the 12 test cases, dStruct reported 3 DRRs when searching over 5 nt windows (Fig. 3a). Its performance was similar or better for longer windows (data not shown). For example, it reported no DRRs when searching over 50-nt windows (data not shown). RASA performed comparably to dStruct, reporting 4 DRRs. In contrast, PARCEL and deltaSHAPE reported 61 and 97 DRRs, respectively.

We calculated the false-positive rates at the nucleotide level as the fraction of nucleotides incorrectly reported as positives for a transcript. For dStruct, the rate was 0% in 9 cases and 0.2%, 0.3%, and 2% in the remaining cases whereas the other methods displayed higher rates (Fig. 3b).

dStruct’s superior performance could be attributed to the fact that it overcomes limitations of the existing methods. In particular, RASA and PARCEL do not account for information obtained from untreated control samples. Structure-Seq, however, does integrate it into the resulting reactivities [25]. PARCEL also does not account for coverage variations within a transcript, which is known to be a significant issue [57]. Additionally, dStruct controls for false discoveries by adjusting the *p* values for multiple tests whereas deltaSHAPE and RASA do not. For detailed overview of deltaSHAPE, RASA, PARCEL, and their limitations, see Additional file 1: Sections S1-S3.

#### dStruct identifies DRRs from ligand-mediated structure alteration

**Fig. 4:**
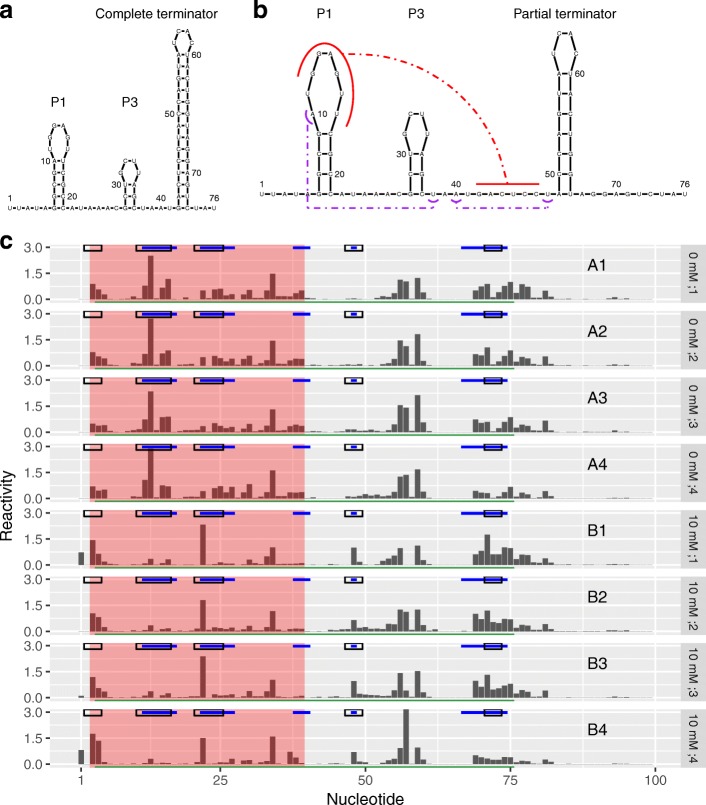
dStruct identified DRRs from a ligand-mediated structure alteration. Fluoride ions bind the *crcB* fluoride riboswitch and alter its structure. **a** Secondary structure in the absence of fluoride ions. **b** Secondary structure in the presence of 10 mM fluoride ions. The red curves highlight the pseudoknot between nucleotides 12–17 and 42–47. The purple curves highlight long-range interactions between the nucleotide pairs (10, 38) and (40, 48). **c** Eight samples of reactivity profiles, four from group A (A1, A2, A3, and A4) with 0 mM fluoride ions and four from group B (B1, B2, B3, and B4) with 10 mM fluoride ions. Solid blue lines mark DRRs that are considered as the ground truth. Hollow black rectangles mark the DRRs called by deltaSHAPE. A red background marks the DRRs called by dStruct. A green line marks the DRR called by PARCEL. Note that RASA did not report any DRRs

Next, we considered cotranscriptional SHAPE-Seq data for the *Bacillus cereus crcB* fluoride riboswitch (100 nt in length), probed in vitro in the absence and presence of fluoride ions [12]. It featured four samples for each group. The presence of fluoride prevents completion of a terminator hairpin by stabilizing a pseudoknot (Fig. 4a, b). Such a mechanism allows fluoride-mediated transcription control. Between the conditions with and without fluoride, nucleotides 12–17, 38, 40, and 67–74 have altered base pairing states [10, 58, 59]. In addition, Watters et al. observed distinct reactivity changes at nucleotides 22–27 from visual examination of an independent dataset [10]. These nucleotides join the P1 and P3 helices but do not have altered base pairing states between conditions. However, these changes were observed consistently over a range of intermediate lengths that were probed cotranscriptionally. Hence, Watters et al. inferred that they were related to fluoride-mediated stabilization of the pseudoknot. Furthermore, we noted a consistent increase in the reactivity at nucleotide 48 in the presence of fluoride, consistent with prior observations by Watters et al. [10]. Given the reproducibility of this change, we regarded nucleotide 48 as differentially reactive. Taken together, we considered nucleotides 12–17, 22–27, 38–40, 48, and 67–74 as our ground truth of DRRs (highlighted in blue on top of each sample in Fig. 4c). Note that in the absence of fluoride, nucleotides 42–47 pair with nucleotides 68–74 and are part of a hairpin. In the presence of fluoride, nucleotides 42–47 pair with nucleotides 12–17 to form a pseudoknot. However, ligand binding is not expected to change the reactivities at nucleotides 42–47 because this region is paired in both the liganded and unliganded states. Hence, we excluded it from the ground truth.

We searched for DRRs using dStruct, deltaSHAPE, PARCEL, and RASA. For comparability, we specified the same window length of 5 nt to dStruct, deltaSHAPE, and RASA. We chose 5 nt because it is the default in deltaSHAPE. The default used by RASA is 50 nt, which is too long for a short transcript of length 100 nt. Note that PARCEL does not require a window length.

dStruct reported a DRR from 3–39, encompassing regions 12–17 and 22–27 and overlapping region 38–40 (region highlighted with red background in Fig. 4c). However, this DRR joined together three separate regions and extended to additional nucleotides towards the 5 ^′^ end. This is due to dStruct’s propensity to screen for the longest possible contiguous regions. While dStruct did not report any false positives, it did not recognize the DRR within 67–74. This region was screened as a candidate but had a *p* value of 0.071 and *q* value of 0.106, both above our desired significance level of 0.05. This is because in this region, the within-group profiles were noisy and not consistently altered between the groups. For example, the reactivity patterns for this region look identical between samples A4 and B4 (Fig. 4c). Additionally, dStruct could not identify the differential reactivity at the isolated nucleotide 48. Indeed, one limitation of dStruct is that it might not report changes at isolated nucleotides even if such changes were real signal. This is due to the fact that differential signals at isolated nucleotides get diluted when scanning over windows. For example, nucleotide 48 is flanked by nucleotides that do not have differential signals. In the “Discussion” section, we propose ways to mitigate this limitation.

Similarly, deltaSHAPE correctly identified DRRs from 11–16 and 21–25, but it also correctly identified 47–49 and 71–73 (marked by black rectangles on top of each sample in Fig. 4c). However, it incorrectly reported region 2–4 and failed to identify region 38–40. PARCEL reported a single DRR that stretched from nucleotides 4–75 (marked by a green line at the bottom of each sample). This DRR correctly encompassed all the real DRRs but also included regions that separate them. RASA did not report any DRRs when searching over 5 nt. It is noteworthy that RASA did not report any DRRs when searching over its default window length of 50 nt either.

Our results highlight that a key difference between the outputs of dStruct, deltaSHAPE, and PARCEL lies in the lengths of DRRs. dStruct identifies contiguous stretches of nucleotides that manifest reactivity changes. While dStruct might join together nearby DRRs, it does so only if they are separated by no more than twice the specified search length. For example, the DRRs from 38–40 and 67–74 are separated by 27 nt with only one differentially reactive nucleotide. This prevents dStruct from extending its reported DRR (3–39 nt) beyond nucleotide 39. In contrast, deltaSHAPE was developed to identify compact regions that might be DRRs. Hence, it yields several short regions as DRRs. Finally, PARCEL was developed to identify the longest possible regions that have at least one nucleotide with significant changes. Thus, it includes long stretches of nucleotides without a differential signal in the reported DRRs. For example, it reported the entire span from the most 5 ^′^ real DRR to the most 3 ^′^ real DRR and included everything in between.

#### dStruct identifies sites of RNA-protein interactions

We tested dStruct on another SHAPE-Seq dataset, which structurally characterizes the HIV Rev-response element (RRE)—a part of a viral RNA intron [11]. RRE binds multiple copies of Rev protein to form a complex that facilitates export of unspliced viral transcripts from the nucleus to the cytoplasm during late stage of HIV infection. Regions of Rev-RRE interactions have been identified using independent methods and provided us with a ground truth for comparisons (Additional file 1: Figure S3) [11, 60–62]. We obtained reactivity profiles for six samples — three replicates each in the presence and absence of Rev. However, counts and coverage information were not available. When searching for regions of length 5 nt or more, dStruct identified 10 DRRs that overlapped 6 of the 7 regions known to bind Rev. However, two of the reported DRRs were false positives. As RASA, PARCEL, and deltaSHAPE require coverage information, we could not apply them to this dataset. At this point, it is worth noting that RASA and PARCEL are based on counts and do not accept reactivities directly. Hence, they are not compatible with available datasets that contain only reactivities or with computational methods that output reactivities [30, 31].

### Validation with large datasets

We tested the methods on two large datasets, one with simulated DRRs and another with DRRs due to known single-nucleotide variants. In all tests, dStruct outperformed the existing methods.

#### dStruct identifies simulated DRRs with properly controlled FDR

We used simulations to assess the methods’ capability in discovering DRRs de novo from transcriptome-wide SP data. To this end, we obtained three replicate samples of the *S. cerevisiae* mRNA structurome using in vivo DMS probing (see the “Methods” section). Next, to mimic realistic trends in within-group variation, coverages, and transcript lengths, we introduced simulated DRRs into these samples. One of the samples was randomly labeled as group A, and the other two were labeled as group B. To start with, we randomly selected 1000 regions in the transcriptome for DRRs. The length of each region was chosen in the range of 50–75 nt, which is the usual range of lengths for search of structured regions [34]. Note that while we simulated the structural profiles for groups A and B over this range, we allowed the simulated DRRs to be shorter, as described next.

RNAs often adopt multiple structural conformations, and reactivities summarize measurements over the entire structure ensembles. Hence, we obtained reactivities for selected regions as ensemble-weighted average of profiles simulated for structures in an ensemble. For each of the selected regions, we sampled up to 1000 unique secondary structures using the ViennaRNA package [63]. Each of the unique structures was assigned an ensemble weight that reflected its proportion in the structure ensemble. The ensemble weights were randomly sampled from arbitrarily chosen probability density functions (see the “Methods” section). For each group, we selected up to five structures that were assigned high weights and hence dominated the overall reactivity profile for that group. The reactivity profiles differed between groups due to the disjoint selection of dominant structures. We introduced within-group variation by adding noise to ensemble weights. In addition, we controlled the between-group variation by controlling the weight of the minimum free energy (MFE) structure in each group. For example, increasing the weight of the MFE structure in both groups increased the similarity of their structure ensembles, thereby reducing the between-group variation. For each structure, we generated a DMS reactivity profile by sampling reactivities using probability density functions for reactivities of paired and unpaired nucleotides [54]. The probability density functions were obtained by fitting a Gaussian mixture model to our data using patteRNA [33, 64]. The final reactivity profile for each region was obtained as the ensemble-weighted average of profiles for individual structures (see the “Methods” section for details).

Overall, we simulated a range of within-group and between-group variations in reactivities, as reflected in the resulting within-group and between-group Pearson correlation coefficients (Additional file 1: Figure S4A). Since all simulated structures for a region represented folding of the same short sequence, there were stretches within them that did not have altered base pairing states between the groups. Indeed, the pairing states were altered for stretches shorter than the complete chosen regions (median length 11 nt; Additional file 1: Figure S4B). Therefore, we ran dStruct, RASA, and deltaSHAPE with a search length of 11 nt. As noted earlier, PARCEL automatically determines the appropriate length for each DRR.

We evaluated the methods in terms of power and observed FDR. Power was calculated as the proportion of simulated DRRs that overlapped at least one reported DRR. The observed FDR was calculated as the proportion of reported DRRs that did not overlap any simulated DRRs. We observed the following performances.

**Fig. 5:**
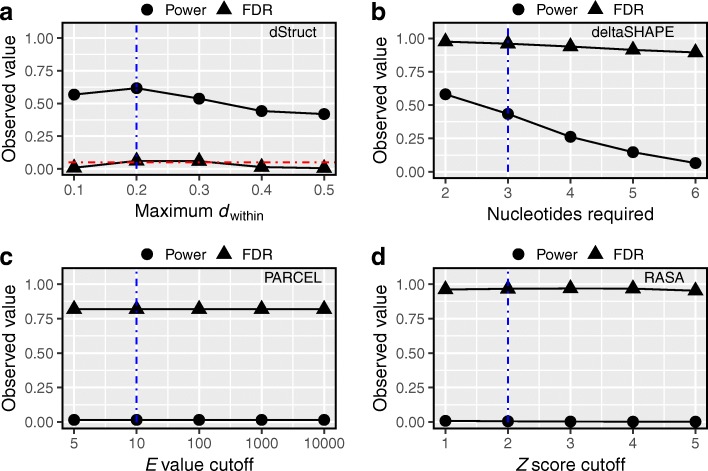
dStruct properly controlled the false discovery rates in simulated data. We searched for DRRs in simulated data using dStruct, deltaSHAPE, PARCEL, and RASA (**a**–**d**, respectively). The powers (circles) and FDRs (triangles) are plotted for each method. We tested each method for a range of stringency levels. Vertical dotted blue lines mark the default parameter settings. A horizontal dotted red line in **a** marks the specified target FDR. deltaSHAPE, PARCEL, and RASA do not control for FDR. *X*-axis labels indicate the parameter tuned for each method. **a** dStruct calls a candidate region a DRR only if it satisfies a quality threshold as well as has significant *p* value and *q* value. The quality threshold is specified in terms of a maximum allowed within-group variation, measured as the average *d*_within_ in the region. **b** deltaSHAPE chains together differentially reactive nucleotides as DRRs if a minimum number of them are colocalized within a specified search length. **c** PARCEL quantifies the statistical significance of structural changes in a region in terms of an *E* value. Under the null hypothesis of no differential signal, it is computed as the number of regions that can be expected to have structural change scores at least as high as the given region’s score. **d** RASA identifies DRRs as the regions that have significant clustering of nucleotides with large changes in reactivities. The significance of the observed clustering is evaluated by comparing the observed distribution of the numbers of such nucleotides in sliding windows of a specified length with their null distribution obtained from permutations. The comparison is done in terms of standard *Z* scores

We tested dStruct’s performance for several values of the minimum quality threshold (see the “Methods” section and Additional file 1: Figure S2). The threshold was specified in terms of maximum dissimilarity of reactivity profiles within the same group, i.e., maximum *d*_within_. We observed that dStruct had reasonably high power (∼ 60%) to discover DRRs for a range of the quality threshold (Fig. 5a). In addition, its FDR was properly controlled to the specified target level of 5%.

deltaSHAPE calls DRRs based on the number of nucleotides in a region that manifest significant changes in reactivities (see the “Methods” section and Additional file 1: Section S1). Requiring fewer nucleotides amounts to a less stringent criterion. We tested deltaSHAPE’s performance for a range of stringency levels. We observed consistently high FDR in its detections (Fig. 5b). For the least stringent criterion, deltaSHAPE’s power was comparable to that of dStruct, albeit at the cost of excessive FDR (Fig. 5a, b). Its high FDR could be attributed to its tendency to always report DRRs in transcripts that have high coverage (see Additional file 1: Section S1 for a detailed overview of deltaSHAPE and its limitations). This is because deltaSHAPE calls DRRs from locally smoothed reactivity profiles. However, smoothing artificially spreads noise at a nucleotide into neighboring nucleotides. This might amplify the noise leading to false appearance of a strong differential signal. In addition, deltaSHAPE does not account for biological variation and does not control for false discoveries.

PARCEL calls DRRs based on the *E* value statistic, which quantifies the statistical significance of reactivity changes in a region (see the “Methods” section and Additional file 1: Section S2 for details). A lower cutoff for *E* values represents a more stringent criterion. We tested PARCEL’s performance on a range of cutoff values (Fig. 5c). We observed a consistently high FDR (∼ 82%) and low detection power (< 1%). PARCEL’s poor performance could be attributed to the fact that it was designed to work in conjunction with a specific SP technology [21]. As such, it does not consider untreated samples or coverage variations across a transcript, which are important issues in transcriptome-wide data from most technologies [6, 30] (see Additional file 1: Section S2 for a detailed overview of PARCEL and its limitations).

RASA calls DRRs in two steps. It uses a generalized mixed model to quantify the significance of reactivity changes for each nucleotide. Then, it identifies regions enriched in differentially reactive nucleotides via permutation testing (see the “Methods” section and Additional file 1: Section S3). Since RASA quantifies enrichment in terms of *Z* scores, we assessed its performance for a range of *Z* score cutoffs (Fig. 5d). The lower the cutoff, the less stringent was the criterion for calling a DRR. We observed that RASA consistently yielded excessively high FDR and very low power. This could be explained by the fact that it does not utilize untreated samples to compute reactivities. Hence, its application might not be suitable for SP technologies like Structure-Seq, which relies on untreated samples [25]. In addition, RASA does not perform multiple testing correction to control for false discoveries (see Additional file 1: Section S3 for a detailed overview of RASA and its limitations).

Overall, we conclude from these comparisons that dStruct has higher power than existing methods and that its observed FDR is properly controlled to the specified target of 5%. We provide additional performance summaries for all methods in Additional file 1: Figure S5. Interestingly, we found that the proportions of transcript lengths reported by dStruct as DRRs correlated well with their simulated ground truths (Additional file 1: Figure S6). This did not hold for the other methods.

Finally, we assessed the effect of varying the specified search length on dStruct’s performance (Additional file 1: Figure S5F). We found that dStruct’s power remained approximately constant up to a search length of 25 nt, from which point it monotonically decreased. This is expected because a higher minimum length excludes more regions whose alterations span stretches shorter than the search length.

#### dStruct identifies DRRs caused by single-nucleotide variants

We compared the performances of dStruct and StrucDiff in a guided discovery context with PARS data for human RNAs by Wan et al. [9]. PARS utilizes a pair of nucleases as probing reagents, and the degrees of reactions from the nucleases are summarized as a PARS score for each nucleotide. The PARS dataset from Wan et al. features RNAs obtained from cell lines derived from a family trio of a father, a mother, and a child, with no replicates for any cell line. Wan et al. obtained a list of transcripts with single-nucleotide variants for this trio and identified DRRs of lengths 11 nt with the variants at their centers. To this end, they developed the StrucDiff approach (Additional file 1: Section S4). For each variant, they compared each pair of individuals separately using StrucDiff. They called a region a riboSNitch (i.e., a regulatory element whose structure is altered by a single-nucleotide variant) if any of the pairwise comparisons for the region yielded a significant result.

StrucDiff has five steps. Given a pair of profiles, first, the data is locally smoothed using a rolling mean over sliding windows of 5 nt to calculate smoothed PARS scores. Second, the absolute difference in the smoothed PARS scores (denoted as $\Delta {\overline {r}_{i}}$ for nucleotide *i*) is calculated. Third, given the variant’s location, the structural change score around the variant (henceforth, called *v*_SNV_) is calculated as the average $\Delta {\overline {r}_{i}}$ for the nucleotides flanking it. In the fourth step, StrucDiff assesses the statistical significance of *v*_SNV_. To this end, it permutes the sequence of $\Delta {\overline {r}_{i}}$ 1000 times. For each permutation, it assesses a structural change score under the null hypothesis (henceforth, called *v*_null_). A *p* value is assigned to the variant as the fraction of *v*_null_ values greater than *v*_SNV_. In addition, StrucDiff controls the FDR using the Benjamini-Hochberg procedure. Finally, a variant region is classified as a riboSNitch if it has significant *p* values and *q* values, *v*_SNV_>1, high local coverage, and high signal strength in a window of 11 nt.

**Fig. 6:**
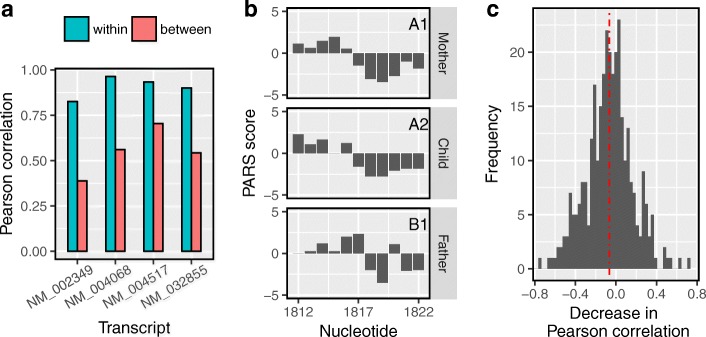
dStruct reported riboSNitches from a PARS dataset. **a** The within-group Pearson correlation coefficients (green bars) for riboSNitches reported by dStruct were higher than their respective between-group Pearson correlations (red bars). **b** Example of reactivity profiles for the mother, the child, and the father for one of the regions that dStruct reported as riboSNitch, i.e., the single-nucleotide variant at site 1817 for NM_032855. Note that for this region, the mother and child were allelically identical and therefore labeled as group A (A1 and A2). They appear identical, but they differ from the father, who had a different allele and was labeled as group B (B1). **c** A histogram of the differences between Pearson correlation coefficients between and within groups. Many of the riboSNitches reported by StrucDiff had only a minor change in their Pearson correlation. For many of the regions, the between-group Pearson correlations were also higher. The dotted vertical line in red marks the median of distribution

Of the regions examined by Wan et al., only those found to be riboSNitches were reported. For our analysis, we selected those for which two of the three individuals were allelically identical, i.e., they were either both heterozygous or both homozygous with the same allele. However, none of the studied cell lines were probed in replicates. Hence, we used profiles from the two cell lines with identical allele at a variant site as two replicate samples of the same PARS profile (labeled group A) for a region of 11 nt centered at the variant. This is reasonable under the assumption that the variant at the center of a region is the only distinguishable structure-altering factor. The remaining cell line with a different allele (labeled group B) could potentially have a significantly altered profile in this region. Hence, we used dStruct (guided discovery mode) to identify the regions with variants that were DRRs. Since there were no independent validations that could provide a ground truth for the variants under consideration (see the “Methods” section), we resorted to an indirect way of comparing the results from dStruct and StrucDiff using the Pearson correlation coefficient. The correlation between a pair of differentially reactive profiles is expected to be lower than the correlation between the samples of the same group [35]. Hence, we calculated the within-group and between-group Pearson correlation coefficients for each region. We found that the within-group correlations for DRRs identified by dStruct were high (Fig. 6a). In addition, the between-group correlations were substantially lower in comparison with the within-group values. This trend in within-group and between-group correlations is expected because dStruct aims to find regions where the between-group variation exceeds that within groups. We further confirmed the similarity of reactivity patterns within groups and their dissimilarity between groups by visual inspection (Fig. 6b, Additional file 1: Figure S7). The inferences from the Pearson correlation coefficients and visual examination support our previous finding of a properly controlled FDR by dStruct. In agreement with dStruct, for two of the DRRs, StrucDiff consistently found significant changes in both the pairwise comparisons of profiles between groups. However, for each of the remaining two DRRs reported by dStruct, it inconsistently called a DRR in one pairwise comparison but not in the other. This is anomalous because both pairwise comparisons involved the same pair of variants.

To glean the FDR of StrucDiff, we took the difference of the between-group correlations and the within-group correlations for all regions. For DRRs, the difference should be significant and negative. However, we found that for many of the regions, the difference was positive, with a median − 0.06 (Fig. 6c). This suggests that there could possibly be a significant proportion of false positives reported by StrucDiff. In other words, StrucDiff’s FDR might be higher than the specified level of 0.1 as well as than that of dStruct. An alternative explanation for this observation could be that the variants at the center of the examined regions were not always the only relevant factors that influenced local structures. In fact, Wan et al. proposed that co-variation of variants in the close vicinity of a variant under consideration might influence the local structure. However, they also found that riboSNitches (identified using StrucDiff) have fewer variants around them in comparison with variants that do not alter structure. Nonetheless, it is possible that our starting assumption that allelic similarity implies the absence of a DRR does not apply for at least some of the variants. This would explain the low proportion of riboSNitches found by our method. It could also explain the anomalous distribution of changes in the correlations for the riboSNitches reported by StrucDiff. Note that we could not compare the powers of dStruct and StrucDiff due to the lack of a ground truth for the data. Besides a comparison of FDRs, it is worthwhile to observe that the permutation test approach utilized by StrucDiff might not be suitable for locally smoothed reactivity data (Additional file 1: Section S4). This is because local smoothing introduces local correlations in $\Delta \overline {r}_{i}$. However, these local correlations are absent in the permuted data. As such, the sampling distribution of *v*_SNV_ under the null hypothesis turns out to be different from the distribution of *v*_null_ values, which can lead to inflated error rates [65] (see Additional file 1: Section S4 and Additional file 1: Figure S8).

## Discussion

### Accounting for biological variation in reactivity patterns

Biological variation in measurements from samples of the same group has been observed across all areas of genomics [66]. In fact, RNA biologists that use SP protocols have been aware of its presence [6, 30]. A recent study by Selega et al. shows that accounting for biological variation improves the estimates of reactivities [30]. Two methods, PARCEL and RASA, which explicitly account for biological variation in the context of differential analysis, have also been published recently [20, 21]. PARCEL uses edgeR to compare the counts between the groups of samples [36]. However, it does not consider coverage variation within a transcript, which is known to be significant [57]. RASA accounts for coverage variation, but similarly to PARCEL, it does not use untreated control samples in computing reactivities. Instead, it assesses the background noise from the untreated samples and then excludes from analysis nucleotides whose noise level exceeds a threshold. It favors this strategy because it was developed to be used with DMS-MaPseq, which does not consider untreated samples in reactivity estimation [67]. Yet, this limits the detection power in transcriptome-wide data from other technologies by filtering a major fraction of the nucleotides because these datasets are highly noisy [7]. Additionally, this places the burden on the user to optimize the threshold level for noise. Recently, broadly applicable computational methods for reactivity estimation have been developed, namely, PROBer and BUM-HMM [30, 31]. These address several challenges in estimating reactivities from transcriptome-wide data, e.g., multi-mapping reads, background noise, and coverage variation. Therefore, it is necessary for novel differential analysis methods to either address these challenges directly or be compatible with methods such as PROBer and BUM-HMM. However, RASA and PARCEL neither account for some of these major issues nor are they compatible with the analog reactivities output by said methods. The incompatibility arises because RASA and PARCEL were designed to take read counts as their (digital) input. Hence, the need for a robust differential analysis method remains unmet for the majority of SP technologies.

Besides accounting for biological variation, it is desirable to identify regions that display differences in their reactivity patterns [33]. An altered pattern in a region could indicate a change in the composition of its structural ensemble [33, 42, 44, 64]. Reactivity pattern is defined collectively by the reactivities of all the nucleotides in a region (Fig. 1a). Hence, one must consider every nucleotide in a region for inferences on pattern changes. However, RASA, PARCEL, and deltaSHAPE first evaluate the changes at individual nucleotides and subsequently chain nucleotides with significant changes together as DRRs. Furthermore, the criteria for the number of nucleotides with significant changes are not always stringent (see Additional file 1: Section S1-S3). For example, PARCEL requires only one significantly altered nucleotide to call a DRR. In contrast to these three methods, StrucDiff considers all nucleotides in a region but only after smoothing the read counts (see Additional file 1: Section S4). This effectively obscures the reactivity patterns. classSNitch is the only method that explicitly accounts for patterns (see Additional file 1: Section S5). However, it does not account for biological variation and is also currently limited to SHAPE data. dStruct presents a major advance over existing methods as it accounts for biological variation and reactivity patterns and is also compatible with diverse technologies. Notably, it smoothes the *d* scores in the second step but only to construct candidate regions. Once constructed, it reverts to the unsmoothed *d* scores to perform the statistical inference (Fig. 2). In guided discovery, it does not perform smoothing at all.

Our approach deviates from the methods for differential analysis of other kinds of high-throughput data, which do not generally account for signal patterns, because our feature of interest is reactivity pattern. For example, in differential methylation studies, the feature of interest for a region is the average methylation level. Such a feature could be described as higher or lower when comparing two samples [48, 49, 66]. However, reactivity pattern is a geometrical feature. As such, it cannot be described as being higher or lower when comparing samples. It has to be described in terms of agreement in reactivity pattern, which can be numerically captured in a transformation of the data. For example, a secondary feature of the data could be assessed, such as the sequence of slopes of segments joining reactivities for two adjacent sites (see slopes of segments of red line in Fig. 1). If two profiles were parallel, the slope of the segment between any pair of adjacent sites would be the same for both profiles. Indeed, this idea forms the basis for the classical approach of profile analysis for test of parallelism [68]. However, this approach requires a large sample size to account for biological variation and can potentially be applied only to predefined regions. In addition, it would require normality of reactivities. If two profiles were to be found as parallel by this approach, they could be tested for coincidence in a second hypothesis test. Since the telling feature for DRRs is coincidence of profiles, dStruct assesses coincidence at the nucleotide level directly in terms of *d* score. Two profiles can be classified as coincident if the vertical distance (or difference in reactivity) between them is 0 at each nucleotide. However, such a definition would be applicable only for two profiles. Our dissimilarity measure, *d* score, extends the concept of pairwise “vertical distance” to multiple profiles. We use *d* score to assess dissimilarity within groups and between groups. Then, we test the null hypothesis that profiles for the two groups are coincident and *d* scores are not significantly different within and between groups.

Our dissimilarity measure is based on the mean and standard deviation of reactivities for each nucleotide. In differential analysis studies in other fields, it has been noted that when standard deviation from very few samples is used to estimate *t*-type test statistics, the test statistics can be unreliable and lead to false positives and reduced power [69]. However, despite this issue, dStruct has reasonably high power and low observed FDR. dStruct’s high performance is possible because we do not utilize standard deviation to assess a test statistic directly. Instead, it contributes to the assessment of a secondary feature of the data. Additionally, from the point-of-view of Wilcoxon signed-rank test, the test statistic in our method is the sum of signed ranks. This test statistic pools information from all nucleotides in a candidate region, and hence, its susceptibility to noise in nucleotide-level *d* scores is reduced. While untransformed ratio of *μ*_*i*_ and *σ*_*i*_ is very sensitive to small changes in *μ*_*i*_ when *μ*_*i*_ is close to zero, we have improved our dissimilarity measure with a monotonic transformation (see Additional file 1: Figure S9). Yet, it is to be noted that our approach focuses on variation at the level of reactivities. Indeed, in our analysis, we have not modeled the mean-variance relationship of reactivities. While dStruct provides a significant improvement over existing methods in its current form, accurate models of heteroscedasticity in mean-variance relationship might enhance the dStruct’s performance. For example, methods for differential gene expression analysis utilize such models [70]. Moreover, we do not model variation directly at the level of counts. In the future, it might be possible to achieve better performance with rigorous models for variation directly at counts level [49].

Finally, our approach differs from other methods of differential analysis in one additional way. For each region of interest, other methods assess a single test statistic [48, 49, 69]. To classify the region, they either rely on a cutoff or assess statistical significance in reference to a null distribution. Moreover, the null distribution is generally obtained by permuting the sample assignment labels for data points and calculating test statistic for permuted data. In contrast to the approach of capturing the effect size and within-group variation in a single test statistic for each region, our approach of quantifying within-group and between-group variations in reactivity patterns provides two vectors of *d* score profiles for each region. The vectors consist of values that reflect nucleotide-level dissimilarity of reactivity patterns. Given these vectors, our goal is to test if between-group variation is significantly higher than within-group variation, in which case it can be reasonably classified as a DRR. Hence, we forgo label permutations in favor of the Wilcoxon signed-rank test. Wilcoxon signed-rank test is an alternative to paired Student’s *t* test and does not assume normal distribution of *d* scores. It compares *d* scores for within-group and between-group variations. In other words, our method assesses the significance of differential reactivity in a region by comparing it to within-group variation in that region. In fact, experts can identify altered reactivity patterns by inspecting a region alone, without needing to resort to transcript-wide or transcriptome-wide data for reference. This suggests that there is adequate information within the candidate regions for classification purpose [35]. Our approach in dStruct takes advantage of this characteristic of the reactivity data. In addition, such an approach of significance testing confers robustness to the presence of outliers or poor quality data outside the regions of interest.

### Limitations

While we found that dStruct can identify DRRs with reasonable power and a properly controlled FDR, several limitations are worth noting. First, dStruct does not automatically identify a search length for DRRs (i.e., the minimum allowed length). With little known about RNA structures, users might not a priori know the optimal search length. Importantly, the analysis results can vary depending on the search length. For example, consider the impact of decreasing the search length from *l*_1_ to *l*_2_. Given the new search length, in addition to identifying the same candidates that were found with length *l*_1_, dStruct might identify additional ones, which are shorter than *l*_1_. While the *p* values of candidates common to both searches should remain the same, their *q* values might change. This might lead to loss of power if the true DRRs were generally longer than *l*_1_. On the other hand, if the true DRRs were shorter than *l*_1_, specifying a minimum search length of *l*_1_ might also lead to loss of power. This is because dStruct disregards all evidence of between-group variation in regions shorter than the specified search length. In our simulations, we found that for a wide range of input search lengths (5–25 nt), dStruct maintained approximately constant power and properly controlled the observed FDR (Additional file 1: Figure S5F). However, this might not always be the case.

Another limitation to note is that dStruct might not determine DRR boundaries accurately, as it opts for the longest contiguous regions possible. Thus, it might join DRRs that are separated by fewer nucleotides than the search length. Moreover, dStruct might miss regions where a majority of the nucleotides have zero reactivities. While zero PARS scores could be considered no information, zero SHAPE/DMS reactivities may report either high-quality information or no information (e.g., a manifestation of high background noise) [37]. In our experience, for PARS as well as SHAPE/DMS data, a substantial fraction of the nucleotides have zeros across all replicates. Considering all of them as high-quality information and defining their *d* scores as 0 results in erroneous inferences (data not shown). Hence, we leave the *d* score for such nucleotides as undefined. Yet, it is worth noting that the quality criteria that we use to filter candidate regions ensure that no more than a small fraction of the nucleotides have undefined *d* scores in candidate regions (see the “Methods” section). Regions containing zero or very low reactivities for most nucleotides are not found by dStruct, even if they are true DRRs. In addition, if one of the groups manifests only zero reactivities in a region, it does not contribute to the assessment of within-group variation in that region.

Another limitation of dStruct is that it leaves the burden of normalizing the reactivities to the user. Normalization is a common practice in the field and aims to bridge differences in reaction conditions [6]. Several approaches have been utilized, which heuristically identify outliers and subsequently use the remaining values to determine a normalization constant [56, 71]. Thus, they critically depend on outlier detection. However, outliers are typically noisy and can easily distort the scaling [6]. Furthermore, their prevalence and characteristics in the context of transcriptome-wide SP are still poorly understood. For these reasons, proper normalization is a critical step in differential analysis, and when done well, it could substantially enhance power. A hallmark of proper normalization is good agreement between the normalized replicates [72]. In that context, we designed dStruct to consider only those regions which satisfy a minimum requirement for replicate agreement (see the “Methods” section for minimum quality threshold and Additional file 1: Figure S2). Specifically, transcripts or regions thereof, which display poor replicate agreement, are filtered by dStruct. We caution users to check for agreement between replicates from the same group in those regions that dStruct discarded. Furthermore, if it excluded a large fraction of the transcripts due to quality considerations, this could suggest that samples were not properly normalized.

Another limitation of dStruct and all other methods is that they might miss the *differentially structured* regions if they do not manifest differential reactivities, as there might be regions in a transcript that pair with alternative partners between groups. For example, nucleotides 42–47 of the *crcB* fluoride riboswitch (Fig. 4a, b) change partners between groups but remain paired in both groups. Such nucleotides might not exhibit significant reactivity changes. Notably, this limitation is due to the nature of SP data.

Besides these limitations, dStruct might miss DRRs that exhibit significant changes at only one to two nucleotides. For long search lengths, it might even overlook such DRRs as candidate regions. This is because differential signals concentrated at only a few nucleotides get diluted when searching over windows. The longer the search length, the more is the signal dilution. Notably, specifying a short search length might not remedy this issue, as it arises from the limited power of the Wilcoxon signed-rank test when applied to very small samples. For example, at a significance level of 0.05, this test cannot identify DRRs shorter than 5 nt in length. This places a hard limit on dStruct’s detection power. Nevertheless, regions shorter than 5 nt might be listed with insignificant *p* values if the specified search length were ≤ 5. Hence, if it is of interest to find isolated single-nucleotide changes, users can specify a short search length and visually examine all the candidate regions. Detection power in such a case could also be improved by replacing Wilcoxon signed-rank test with paired *t* test, which might be more powerful for small samples [73].

Some of the dStruct’s limitations could be mitigated. It is possible that in a study, RNAs are expected to have altered reactivity patterns over multiple non-contiguous regions, yet no region has a sufficiently strong effect size. In such a case, the detection power could be improved by testing all candidate regions identified within an RNA collectively (see dStruct’s manual). Note, however, that this assesses the significance of differences at the level of a transcript and not a region. This distinction should thus be clearly reported. An alternative scenario is that the biological question warrants a short search length, but due to the noisy nature of data, it results in screening of candidate regions that do not represent DRRs. This might impact the detection power because non-DRR candidates impact the correction for multiple tests. However, it is plausible that the real DRRs among the candidates are closely located, say separated by 5–10 nt, while the non-DRR candidates are separated by larger distances. In such cases, power could be improved by leveraging the proximity of real DRR candidates and testing candidate regions collectively if they are located within a certain distance of each other. Another way to improve power in such situations is to integrate differential analysis of SP data with other kinds of relevant data. For example, consider a study on how a protein impacts RNA structure upon binding. Let there be two groups with wild-type samples and samples where the protein’s binding domain has been eliminated. Let us say that dStruct is given a search length of 5 nt and constructs a lot of candidate regions but calls no DRRs due to the subtleness of reactivity changes. It might be possible to integrate the information from a collateral study on sites of RNA-protein binding with regions constructed by dStruct and perform enrichment tests. The null hypothesis underlying such a test could be that constructed regions are not associated with the change in protein’s function. Such tests have been used in other fields of genomics to yield useful biological insights, e.g., gene set enrichment analysis [74, 75]. Future developments of such methods specialized to the SP data could benefit RNA structure studies. Furthermore, it might be possible to do SP in the presence of a range of concentrations of wild-type protein [11]. This would result in several groups of samples with different concentrations of the protein. Differential analysis of such data could be performed in the following manner. One could compare each group of samples to the group with the lowest concentration of the protein. Emergence of certain constructed regions as the difference in concentrations between groups increases might reveal the DRRs. If such regions consistently appear beyond a level of concentration differences, they could be considered as evidence in support of DRRs.

### Additional recommendations

We strongly recommend using dStruct in conjunction with the data obtained from paired-end reads. While dStruct works with both single- and paired-end reads, reactivities are most reliable when the treated and untreated detection rates are estimated using local coverages instead of transcript-level coverages [6, 37]. In addition, it is critical to secure at least two samples per group. In our experience, reactivity patterns could change merely due to biological variation. In the absence of replicates for a group, it does not contribute to the estimates of within-group variation. This might lead to false positives. Moreover, in some studies, one of the groups might be expected to manifest much higher variation than the other due to experimental limitations and/or known biological factors. For example, Watters et al. compared genome segment RNA3 of the *Cucumber mosaic virus* between infected cell lysates (group A) and in vitro refolded viral RNA extracted from virions (group B) [13]. They observed much higher variation in group A than in group B. In such a case, *d*_within_, which summarizes the within-group variation in both groups, might be very high and thereby limit the detection power. However, as has been recently done in methylation studies [48], it may be possible to enhance dStruct’s power by assessing *d*_within_ only for the less variable group (see dStruct’s manual for details). DRRs found using this approach would represent regions where one group varies much more than the other. Importantly, if such an approach was utilized, the supporting details should be clearly reported. Finally, in guided discovery situations, it is possible that collateral studies do not pinpoint the exact regions where DRRs might be found but only indicate their approximate locations. For example, in RNA-protein interaction studies, DRRs might be anywhere within say 100 nt upstream/downstream of CLIP-seq signal peaks. In this case, performing guided discovery with say a 20 nt window centered at the peak may preclude the discovery of more distant DRRs. However, de novo discovery within the entire transcript may not be optimal either. Hence, if the precision of the CLIP-seq data was known, it may be better to perform de novo discovery with say a 200-nt window centered at the peaks.

## Conclusions

We described dStruct, a novel approach to identify DRRs from SP data. dStruct is compatible with diverse SP protocols and accounts for biological variation in SP data. First, it quantifies the within-group and between-group variation. Then, it constructs regions that are potential candidates for DRRs to facilitate de novo discovery or utilizes candidate regions identified by collateral studies to aid guided discovery. Finally, it assesses the statistical significance of differential reactivities in candidate regions and controls for false discoveries. To validate dStruct, we used diverse datasets, which span a range of SP technologies, structure-altering factors, and organisms. We demonstrated that for a properly controlled FDR, dStruct has a higher power than existing approaches. While we validated dStruct with the SHAPE-Seq, Structure-Seq, and PARS protocols, it is applicable to many other SP technologies. With SP technologies reaching the phase of maturation, there is a need to develop robust methods to perform differential analysis of SP data. We discussed the unique aspects of SP data that distinguish it from other kinds of genomic data. These unique aspects present a need for differential analysis methods tailored to the needs of diverse SP technologies. dStruct is a first step in this direction.

## Methods

### Quantifying dissimilarity of reactivities

We used a *d* score to quantify the dissimilarity of reactivities. Its definition was motivated by the need for a robust measure of agreement/disagreement in reactivity patterns in a transcript or in regions thereof. We devised the *d* score by examining the deficiencies of existing approaches in serving this need. For example, classSNitch utilizes a feature of reactivity profiles called a pattern correlation coefficient. It is the Pearson correlation coefficient of sequences of signs of slopes of the segments joining reactivity scores for adjacent nucleotides in plots of reactivity profiles (slope of segments of red line in Fig. 1a). While correlation in sequences of slopes could assess if two profiles were parallel, a region with approximately parallel profiles might still be a DRR if the profiles were not coincident. classSNitch assesses the coincidence in profiles by taking the Euclidean distance between a pair of profiles. However, the Euclidean distance is valid for only two profiles and can be sensitive to outliers. At nucleotide resolution, the coincidence of two profiles could be captured as the vertical distances between the profiles or the differences in reactivities. If the differences were zero or significantly low for a pair of profiles, they might be called coincident. This is the basis of structural change scores used in deltaSHAPE and StrucDiff. However, the utility of reactivity differences is limited to a comparison of two profiles. Besides the need for a measure that could simultaneously summarize the agreement of reactivity patterns for more than two replicates, we identified a need to account for the fact that nucleotides with higher average reactivity tend to have higher fluctuations [56]. This aspect of the data could be accounted for by considering the ratio of the reactivity difference and the mean reactivity at a nucleotide, i.e., if *r*_1,*i*_ and *r*_2,*i*_ are the reactivities in two replicates at nucleotide *i*, we could consider:

3 $$\begin{array}{@{}rcl@{}} \frac{\left| r_{1,i} - r_{2,i} \right|}{\frac{1}{2}\left| r_{1,i} + r_{2,i} \right|}. \end{array} $$

The above expression yields a sequence of zeros for perfectly coincident profiles. It yields a sequence of very high numbers (or infinity) for nearly anti-parallel profiles. For two profiles, *d* score is defined as the arctan of the above expression, with additional scaling as described next. To account for multiple profiles simultaneously, we replaced the numerator in the above expression with the sample’s standard deviation (denoted *σ*_*i*_), which gave us the absolute value of the coefficient of variation, or |CV|. However, |CV| is very sensitive to small changes in the mean reactivity (denoted *μ*_*i*_), especially when *μ*_*i*_ is close to zero (see Additional file 1: Figure S1). This could lead to excessively large |CV|. For example, PARS scores can take both positive and negative values, which can yield *μ*_*i*_ values close to zero. As *μ*_*i*_ decreases and approaches zero, *σ*_*i*_/|*μ*_*i*_| increases very fast and approaches infinity. This is problematic because excessively large values can dominate the averages that we use within the dissimilarity measures (i.e., nucleotide-wise averages across all the homogeneous subsets or all the heterogeneous subsets in step 1; average at the regional level in step 2; see the “Differentially reactive regions” section). Hence, we applied a monotonic transformation to |CV| that prevents the occurrence of excessively large values (Fig. 1c and Additional file 1: Figure S1). While logarithmic transformation is a common choice, it is not suitable for |CV| directly as it goes to −*∞* for |CV| close to 0. Indeed, |CV| being close to zero suggests that the reactivities being compared are identical, which can happen in regions with good data quality. While log transforming (1+|CV|) is a possible alternative, log transformation does not restrict the range of the transformed values. In fact, their range remains the same as that of the untransformed |CV|, i.e., [0,*∞*). Hence, log does not guarantee bounded values in transcriptome-wide data, which displays numerous instances of extremely high |CV| for PARS data (data not shown). We use a transformation that yields values of identical order of magnitude as the log transformation for *σ*_*i*_/|*μ*_*i*_| up to ∼ 10^3^ and which does not increase to infinity for higher values of *σ*_*i*_/|*μ*_*i*_| (Additional file 1: Figure S1). A natural choice to transform ratios is to use inverse trigonometric functions. For example, proportions are often transformed using the arcsin function [49]. However, arcsin’s domain is limited to [−1,1]. Hence, we transformed |CV|, which can take any positive value, using an arctan function—a monotonic transformation that ranges from 0 to *π*/2 (Fig. 1b, c and Additional file 1: Figure S9). arctan(*σ*_*i*_/|*μ*_*i*_|) is approximately equal to *σ*_*i*_/|*μ*_*i*_| for *σ*_*i*_/|*μ*_*i*_|<1. Additionally, for higher values of *σ*_*i*_/|*μ*_*i*_|, it is close to log10(1+*σ*_*i*_/|*μ*_*i*_|) when *σ*_*i*_/|*μ*_*i*_| is less than or around order 10^3^. Importantly, arctan(*σ*_*i*_/|*μ*_*i*_|) asymptotically reaches *π*/2 as *σ*_*i*_/|*μ*_*i*_| increases beyond order 10^3^, whereas log10(1+*σ*_*i*_/|*μ*_*i*_|) continues to increase with *σ*_*i*_/|*μ*_*i*_|. This is a useful property because we do observe *σ*_*i*_/|*μ*_*i*_|≫10^3^ in transcriptome-wide data (not shown). Furthermore, we compared performances of log and arctan transformations in the context of differential analysis with dStruct. In addition, we compared a threshold approach to bound *σ*_*i*_/|*μ*_*i*_| by restricting large values to the threshold. We observed identical performances of all three approaches. This is because dStruct utilizes a non-parametric test. In such a test, only the relative ranks of *d* scores are of concern (Additional file 1: Figure S9). Since log and arctan transformations are both monotonic transformations, using one instead of another alters the absolute magnitudes of *d* scores but not their relative ranks. Nonetheless, for our purpose, the major advantage of the arctan transformation is that it results in values that are bounded to a finite interval. This allows a convenient scaling such that the *d* scores are bound between 0 and 1, which is a desirable feature for interpretation [76] (Additional file 1: Figure S10).

Since arctan(*σ*_*i*_/|*μ*_*i*_|) ranges from 0 to *π*/2, we rescaled it such that it ranges from 0 to 1. Finally, we obtained the following expression for the *d* score:

4 $$\begin{array}{@{}rcl@{}} d_{i} &=& \frac{2}{\pi} \arctan \left(\frac{\sigma_{i}}{\left| \mu_{i} \right|} \right). \end{array} $$

For reactivity scales that are restricted to non-negative values (e.g., SHAPE), the *d* score will never reach the maximum value of 1. For PARS-type data, positive and negative reactivities carry information about the likelihood of a nucleotide forming or not forming a base pair. However, if the PARS reactivities across samples were such that *μ*_*i*_=0, it would imply that some samples indicated the presence of a base pair at *i* while others indicated the contrary, thereby resulting in *μ*_*i*_=0. Hence, *μ*_*i*_=0 is indicative of maximal dissimilarity between reactivities for nucleotide *i*, and *d*_*i*_=1 when *μ*_*i*_=0.

Note that we previously reported an approach to quantify agreement between reactivity profiles, which is similar to *d* score, namely, the signal-to-noise ratio (SNR) [37, 38]. We demonstrated the utility of SNR in quality control of SP data, where we showed that given several samples of the same group, SNR-based analysis could identify the discordant replicates and regions. SNR was defined as the inverse of the CV and was tested only for SHAPE and DMS data. In addition, we dealt with the sensitivity of the mean SNR to small changes in *σ*_*i*_ by restricting its maximum value to 35 based on the properties of the data. The *d* score can be interpreted as a redefinition of SNR. While SNR captures agreement of reactivities, *d* score captures variation. In addition, sensitivity of *d* score to small changes in *μ*_*i*_ was reduced by a monotonic transformation of |CV|. As such, the *d* score could be used to replace SNR in quality control applications (see Additional file 1: Figure S11).

### Overview of dStruct

We developed dStruct to identify DRRs in three steps. In the first step, we assess the within-group and between-group variation. In the second step, we identify regions that could potentially be DRRs. This step is performed only for de novo discovery. In the third step, the regions identified in the second step are statistically tested to detect DRRs.

Given the two groups labeled A and B and *m*_*A*_ and *m*_*B*_ replicate samples from these groups, respectively, let *m*= max(*m*_*A*_,*m*_*B*_). We construct all the possible subsets of the *m*_*A*_+*m*_*B*_ samples, such that each subset has *m* samples. Among these subsets, some will be homogeneous, i.e., all the samples in the subset will come from the same group, whereas others will be heterogeneous. In a subset, suppose there are *g*_*A*_ samples from group A and *g*_*B*_ from group B. For *m*=2, all the heterogeneous subsets will have *g*_*A*_=1 and *g*_*B*_=1. In other words, the ratio of the numbers of samples from the two groups in all the heterogeneous subsets will be 1:1. Similarly, for *m*=3, all the heterogeneous subsets will have either *g*_*A*_=1 and *g*_*B*_=2 or *g*_*A*_=2 and *g*_*B*_=1. The ratio of the numbers of samples from the two groups in all heterogeneous subsets will be 2:1. However, for *m*>3, the heterogeneous subsets can have different ratios. For example, for *m*=4, some heterogeneous subsets will have *g*_*A*_=3;*g*_*B*_=1 or *g*_*A*_=1;*g*_*B*_=3, resulting in a ratio of 3:1, while others will have *g*_*A*_=2;*g*_*B*_=2, resulting in a ratio of 1:1. Hence, for *m*>3, dStruct retains only those heterogeneous subsets which have the highest degree of heterogeneity defined as *g*_*A*_*g*_*B*_/*m*^2^. For each subset, we assess *d* scores as described before “Dissimilarity measure”. We use the nucleotide-wise average of *d* scores from the homogeneous subsets, called *d*_within_, as the measure of the within-group variation in the second and the final steps. Similarly, we use the average of the *d* scores from the heterogeneous subsets, called *d*_between_, as the measure of the between-group variation.

Before we describe the second and the third steps, we note that the *d* score is a sample statistic. Hence, it is best estimated from sets with a large number of samples. To ensure high confidence in the estimated *d* scores, we define the number of samples in the homogeneous/heterogeneous sets as *m*= max(*m*_*A*_,*m*_*B*_). However, this definition could be problematic if *m*_*A*_ and *m*_*B*_ differ by a large number. For example, if *m*_*A*_=5 and *m*_*B*_=1, then under the scheme described above, there will be one homogeneous set with five samples from group A. In addition, there will be five heterogenous sets, each with four samples from group A and one sample from group B. Due to the large concentration of samples from the same group in heterogeneous sets, *d*_between_ might be low. In fact, *d*_between_ might be close in magnitude to *d*_within_, even in the presence of a differential signal. This could reduce the power because we identify DRRs by comparing *d*_between_ and *d*_within_. Hence, if heterogeneous sets have unequal numbers of samples from A and B, i.e., *g*_*A*_≠*g*_*B*_, we adjust *m* such that the resulting heterogeneous subsets would have equal numbers of samples from both groups. Specifically, we adjust *m* by reducing it in decrements of 1, but not below 3. We stop reducing *m* once *g*_*A*_=*g*_*B*_ has been achieved or *m*=3. Notably, we do not reduce *m* below 3 because of the heavy loss in confidence when estimating a statistic (e.g., standard deviation) from two samples instead of three. Hence, whenever possible, we use a minimum set size of three samples to estimate the *d* scores. A properly chosen *m* enables estimation of *d*_within_ and *d*_between_, such that the power could be maximized in the following steps.

In the second step, we identify candidate regions of lengths greater than or equal to a user-specified search length, *l*. To this end, we define *Δd*=*d*_between_−*d*_within_. We smooth *Δd* with a rolling mean over windows of lengths *l*. If the smoothed *Δd* is positive for all the nucleotides in a contiguous region of length *l* or longer, we consider the region a potential candidate for DRR. Additional details on this step are noteworthy. They are implemented to ensure a reasonable quality of identified regions. By default, for the sake of constructing candidate regions, we mask *Δd* for nucleotides with |*r*_*i*_|<0.1 across all samples. Alteration in reactivity patterns due to changes in the relative magnitudes of very low reactivities is not meaningful. Hence, we mask *Δd* for nucleotides with very low reactivities to prevent identification of regions with low signal strength for the majority of nucleotides as candidates. In addition, for the identified regions of length 11 or more, we trim nucleotides with reactivity < 0.1 in all samples from the edges. We do not trim for shorter regions as it can lead to loss of power. Besides this, we require that the identified regions have non-missing *Δd* for at least five nucleotides if *l*>5 and for at least *l*−1 nucleotides if *l*≤5 (i.e., *Δd* not masked due to low signal strength and not 0/0). This might not be the case in poor quality regions (due to lack of coverage or high background noise) or for short regions identified in data from base-selective probes, such as DMS. Finally, we require that the identified regions have no more than an allowed level of average *d*_within_. We impose this requirement because our statistical test only assesses if the between-group variation is significantly more than the within-group variation. However, it is desirable that the reported DRRs have at least moderate correlations within groups to ensure a minimum quality in DRRs. Importantly, filtering out poor-quality, and hence unreliable candidates, before statistical testing could improve power [41]. Hence, we set a liberal threshold for average *d*_within_ in identified regions, i.e., we filter regions with poor within-group correlation but keep those that have moderate to good correlation. In other words, we only filter regions that have highly unreliable reactivity profiles. We call this a *minimum quality threshold*. We require that the average *d*_within_ be < 0.5 if min(*m*_*A*_,*m*_*B*_)≥2 and < 0.2 if min(*m*_*A*_,*m*_*B*_)=1. Note that average *d* scores of 0.5 and 0.2 correspond to mean SNR values of 1 and ∼ 3, respectively; we have previously shown that these SNR values filter regions with poor agreement between replicate samples and/or poor coverage [37], and hence, they are liberal thresholds for quality. We impose a more stringent requirement (chosen based on simulation results) for *d*_within_ if only one sample is available for one of the groups. This is because in such cases, the within-group variation in one of the groups cannot be estimated. Hence, we utilize a less liberal threshold for *d*_within_ to compensate for unavailable quality information from one group. Note that while we do not screen for regions if the user inputs candidate regions (guided discovery), even in this case, we require that the candidate regions have no more than an allowed level of average *d*_within_. The threshold is set identical to that for de novo discovery. Besides this, for guided discovery, we also require that the median of *Δd* in candidate regions be positive for the regions to be called DRRs. We impose this requirement because DRRs are expected to have observable increase in variation from within-group to between-group. The candidate regions that satisfy all the quality criteria are statistically tested in the final step.

In the third step, we obtain the significance of the differential reactivities in a candidate region (obtained in the second step or provided by the user) by comparing *d*_within_ and *d*_between_ for the region. Specifically, we perform a Wilcoxon signed-rank test to test the null hypothesis against a one-sided alternative hypothesis that the population mean of *d*_between_−*d*_within_>0 [52]. The FDR of the screened regions from all transcripts is controlled using the Benjamini-Hochberg procedure [53]. Finally, we obtain a list of regions with corresponding *p* values and *q* values.

It is worthwhile to note that the application of a Wilcoxon signed-rank test to compare *d*_within_ and *d*_between_ requires two assumptions under the null hypothesis. First, we assume that if the null hypothesis were true, then *d*_within_ and *d*_between_ would be identically distributed. This is a reasonable assumption because a true null hypothesis implies that all the samples are identical, irrespective of the groups they belong to. Hence, the *d* scores assessed from the homogeneous and heterogeneous subsets of the samples should have identical distributions. Second, we assume that under the null hypothesis, *Δd* for different nucleotides in a candidate region are independent of each other. This is reasonable under the assumption that *σ*_*i*_ is directly proportional to *μ*_*i*_ [40, 56]. Under this assumption, *σ*_*i*_/*μ*_*i*_ should be a constant plus an error term. In other words, while *μ*_*i*_ might exhibit correlation between adjacent nucleotides, *σ*_*i*_/*μ*_*i*_, and hence *d*_*i*_, should be independent of *μ*_*i*_. Furthermore, autocorrelation in *μ*_*i*_ should not carry over to *Δd*_*i*_’s. We confirmed that this is indeed the case for our Structure-Seq data for three identical replicates. We assessed the autocorrelation in the *μ*_*i*_ profiles for the mRNAs represented in the data. In addition, we randomly assigned one of the replicates to group A and the other two to group B. We assessed the *Δd* profile for each mRNA. Since all replicates were obtained identically, these *Δd* profiles represented values under the null hypothesis. Then, we computed the autocorrelation in each *Δd* profile. For a lag of 1, we found that the *μ*_*i*_’s had median autocorrelation of 0.2, while the *Δd*_*i*_’s had median autocorrelation of 0.02 (Additional file 1: Figure S12). In other words, the *Δd*_*i*_’s were essentially uncorrelated under the null hypothesis.

#### Software

An R package implementing dStruct is freely available online under the BSD-2 license. dStruct utilizes the “parallel” package in R to enable faster processing. In addition, it utilizes the “ggplot2” package to provide detailed plots for differentially reactive regions.

### Structure-Seq library preparation and sequencing

Structure-Seq was adapted from Ding et al. [77]. Yeast cells (*S. cerevisiae*, BY4741) were grown to an O.D. of 0.5 ∼0.7 in 50 mL of YP with 2% glucose at 30 ^∘^C, and then incubated with 10 mM dimethyl sulfate (DMS) for 10 min at 30 ^∘^C with vigorous shaking. To stop the reaction, 75 mL of 4.8 M 2-mercaptoethanol (BME) and 25 mL of isoamyl alcohol were added to the cells. Cells were harvested and pellets were washed once with 5 mL of 4.8 M BME and then once with 5 mL of AE buffer (50 mM sodium acetate pH 5.2, 10 mM EDTA). Total RNA was extracted using acid phenol/chloroform. Polyadenylated RNAs (poly(A) RNAs) were enriched with the Poly(A)Purist MAG kit (ThermoFisher Scientific). The poly(A) RNAs were incubated with TURBO DNase and isolated using acid phenol/chloroform. For each biological replicate, 1 *μ*g of DNase-treated poly(A) RNAs were used to generate cDNAs by SuperScript III (ThermoFisher Scientific) using the random hexamer fused with Illumina TruSeq adaptor (Random-hex RT-primer, Additional file 1: Table S1). This reverse transcription (RT) reaction was performed according to the manufacturer’s instruction. The reaction was then stopped by heating the samples at 85 ^∘^C for 5 min. After the samples cooled down, they were treated with 1 *μ*L of RNase H (5 U/ *μ*L, ThermoFisher Scientific) to degrade residual RNAs at 37 ^∘^C for 20 min. The cDNAs were purified with phenol (pH 8.0)—chloroform extraction and resolved on a 10% denaturing polyacrylamide gel and stained with SYBR Gold. Products with length > 30 nt were collected and eluted from the gel in TEN buffer [77] overnight at 4 ^∘^C. Gel purified cDNAs were ethanol-precipitated, re-suspended in water, and ligated with an ssDNA linker (Additional file 1: Table S1) at 3 ^′^ ends using CircLigase I (epicenter) as previously described [77]. Products > 60 nt were gel purified as above and suspended in 10 *μ*L of water. The ligated cDNAs were subjected to PCR as previously described [77]. To identify potential non-specific primer-dimers in the following steps, a non-template control without any cDNA was also included in the PCR reaction. The products were then resolved on a 10% non-denaturing polyacrylamide gel, and only those above 180 bp were gel purified to eliminate primer dimers. After purification, the library was ethanol-precipitated and re-suspended in water. These libraries were analyzed by Agilent Bioanalyzer to determine the size distribution. A total of six libraries, including three samples with and three samples without DMS treatment, were sequenced on the Illumina Hiseq 2500 platform for 2 × 100 bp paired-end cycle run. Note that we performed paired-end sequencing to ensure accurate assessment of local coverage for reactivity calculations [37].

### Pre-processing of Structure-Seq data

Illumina adaptors were removed from the reads using Trimmomatic (version 0.36). Next, cutadapt (version 1.9.1) was used to trim the random trimers from the 5 ^′^ end of the forward reads. Trimmed reads were aligned to the S288C reference genome (R64-2-1, from the Saccharomyces Genome Database [78]) using STAR (version 2.5.2b) [79] and only uniquely aligned reads (MAPQ = 255 after mapping) were kept for the subsequent analyses. We mapped the reads once to the whole genome sequence and again to rRNA sequences only. We compared mapping to the genome sequence with mRNA annotations to obtain counts and coverages for use in simulations. The mapping to the rRNAs was used for null comparisons as described in the section on validations with small datasets.

The annotation for mRNA untranslated regions (UTRs) was derived as follows. The UTRs for each mRNA were obtained from two published datasets [80, 81]. If the UTR coordinates for the same transcript were different in the two datasets, the coordinates with the widest range were used. For mRNAs without UTR annotations, 135 nucleotides (close to the median lengths of all *S. cerevisiae* 5 ^′^ and 3 ^′^ UTRs) were added before and after the ORF region as 5 ^′^ and 3 ^′^ UTRs. After ignoring genes with sequence overlaps with other genes on the same strand, we retained 4681 mRNAs for use in simulations. Reads were grouped according to their source mRNA, and the start and end indices from genomic alignment of each read were converted to the mRNA coordinates with the start of the 5 ^′^ UTR as position + 1.

Due to multiple copies of rRNA sequences in the genome, reads did not map uniquely to rRNA loci. Hence, we separately mapped the reads to the rRNA sequences after adaptor removal and random trimer trimming. The uniquely mapped reads were grouped according to the source rRNA and the start and end indices of each read were converted to a 1-based coordinate system.

### Reactivity calculations

The reactivity of a nucleotide is a measure of its degree of reaction with the probing reagent. In this study, we used reactivities obtained from the Structure-Seq, SHAPE-Seq, SHAPE-MaP, and PARS protocols. Structure-Seq utilizes DMS as a probing reagent. DMS methylates the base pairing faces of unpaired As and Cs [25]. SHAPE-Seq and SHAPE-MaP utilize SHAPE (*s*elective 2 ^′^-*h*ydroxyl *a*cylation analyzed by *p*rimer *e*xtension) reagents, which form a 2 ^′^−*O*−ester adduct on the RNA backbone [22]. The adduct formation is favored at unpaired nucleotides relative to paired ones. This is because the higher flexibility of unpaired nucleotides enables them to adopt conformations favorable for reaction with the SHAPE reagent. Both DMS- and SHAPE-modified nucleotides impact primer extension by reverse transcriptase. The “-Seq” and “-MaP” approaches differ in how they are impacted by nucleotide modification. In “-Seq” approaches, primer extension stops upon encountering a modified nucleotide [32]. In “-MaP” approaches, primer extension proceeds upon encountering a modified nucleotide but misreads it, thereby incorporating a noncomplementary nucleotide into the cDNA [82]. Besides treating samples with reagents, Structure-Seq, SHAPE-Seq, and SHAPE-MaP utilize untreated samples to assess background noise. On the other hand, PARS utilizes two nucleases, V1 and S1. The V1 and S1 nucleases cleave the RNA strands next to paired and unpaired nucleotides, respectively. A cDNA library is prepared for RNAs treated with the nucleases by primer extension with reverse transcriptase. In all protocols, the cDNA library is sequenced and reads are analyzed to calculate reactivities.

For Structure-Seq and SHAPE-Seq, the number of reads starting 1 nt downstream of each nucleotide were tallied to get the detection counts for the nucleotide (i.e., detection of reagent-induced modifications and noise in treated samples and noise in untreated samples). In addition, the number of reads starting anywhere upstream of, at, or 1 nt downstream of each nucleotide, and ending anywhere downstream of the nucleotide, were tallied as its local coverage. Detection rates were calculated for each nucleotide as the ratio of detection counts to local coverage [32, 37]. Raw reactivities were calculated by combining the information from the treated and untreated samples prepared in the same batch. Raw reactivities, *r*_*i*,raw_, were obtained as:

$$\begin{array}{@{}rcl@{}} r_{i,\text{raw}} & = & \max\left(\frac{r_{i}^{+} - r_{i}^{-}}{1 - r_{i}^{-}}, 0 \right)  \end{array} $$

where $r_{i}^{+}$ and $r_{i}^{-}$ are the detection rates at nucleotide *i* for treated and untreated samples, respectively [32, 83]. Note that it is a common practice to assign a reactivity score of 0 to nucleotides where $r_{i}^{-} > r_{i}^{+}$, which can happen due to high background noise [6]. For Structure-Seq data, due to the base-selective nature of DMS, reactivities for Gs and Us were masked as missing information. This step was skipped for SHAPE-Seq data, as SHAPE reagents probe all four nucleotides. Next, raw reactivities were normalized using a 2–8% approach [56, 84], i.e., the top 2% of reactivities were filtered as outliers and the mean of the next 8% reactivities was used to normalize all the reactivities in that sample. This provided a single sample of reactivity profiles for each batch. Normalized SHAPE-MaP reactivities were available directly from the Weeks lab website. For PARS data, we downloaded the V1 and S1 counts for all transcripts, which were available online [9]. The nucleotide-wise V1 and S1 counts for each cell line were combined as previously described to obtain PARS scores [9], *r*_*i*_, for nucleotide *i*, as:

$$\begin{array}{@{}rcl@{}} r_{i} = \log_{2} \left(\frac{V1_{i} + 5}{S1_{i} + 5} \right).  \end{array} $$

A small number 5 added to V1 and S1 counts in the above equation prevents over-estimation of PARS scores for nucleotides with low coverage. Note that we use *r*_*i*_ to denote PARS scores as well as normalized reactivities from Structure-Seq, SHAPE-Seq, or SHAPE-MaP. Download links for all the datasets used in this study are available in Additional file 1: Table S2.

### Implementation of deltaSHAPE

deltaSHAPE was implemented using the software version 1.0 available for download from the Weeks lab website [8]. In addition to the reactivity of a nucleotide, deltaSHAPE requires the standard error of the reactivity (see Additional file 1: Section S1). It is obtained as the standard deviation of the sampling distribution of the reactivity. It can be computed using theoretical models that require counts and local coverage information for a sample. For the *Xist* long non-coding RNA SHAPE-MaP data, standard errors were available online alongside reactivity data [85]. For SHAPE-Seq and Structure-Seq data, we utilized a simplified expression for a formula derived in our previous publication [37] to estimate the standard error, SE _*i*_, at nucleotide *i*:

$$\begin{array}{@{}rcl@{}} \text{SE}_{i} &=& \frac{1}{f} \sqrt{\frac{r^{+}_{i}}{C^{+}_{i}} + \frac{r^{-}_{i}}{C^{-}_{i}} }, \end{array} $$

where *f* is the normalization constant for the transcript, obtained using the 2-8% approach [56, 84], *r*^+^ and *r*^−^ represent the detection rates at nucleotide *i* for the treated and untreated samples, respectively, and *C*^+^ and *C*^−^ represent the local coverages in the corresponding samples.

### Implementation of PARCEL

To the best of our knowledge, no software implementing PARCEL is available publicly. Hence, we implemented PARCEL to the best of our understanding based on descriptions by Tapsin et al. and email correspondence with them [21]. We identified DRRs in four steps (see Additional file 1: Section S2). We executed all the steps separately for each RNA. First, we ran edgeR on the detection counts for two groups of samples as input [36]. For each nucleotide of a candidate RNA, edgeR outputs the logarithm of fold change in detection counts between the groups. In addition, it outputs *p* values quantifying the statistical significances of changes in counts. Let the *p* value for nucleotide *i* be *p*_*i*_. In the second step, we converted the *p*_*i*_’s to scores, *s*_*i*_:

5 $$\begin{array}{@{}rcl@{}} s_{i} & = & \log \left(0.1 \right) - \log \left(p_{i} \right). \end{array} $$

In simple terms, nucleotides with *p*_*i*_<0.1 received a positive score. We assigned *s*_*i*_=−10 to nucleotides that had 1 or fewer detection counts. In the third step, we utilized a recursive implementation of the Kadane algorithm to identify regions with high aggregate scores [86] (see Additional file 1: Section S2 for details). Given the aggregate score of a region, *S*, we assessed the statistical significance of the structural changes in the region in terms of *E* values. *E* values were defined as *E*=*Ke*^−*λS*^. In simple terms, an *E* value represents the number of regions that are expected to have at least as high an aggregate score as *S* if there were no real differential signal. As such, a lower *E* value indicates a more significant differential signal in a region. The values of *K* and *λ* were derived by Tapsin et al. theoretically. These were *K*=0.0809635 and *λ*=0.862871. Hence, given *S* for a region, its *E* value could be computed. We considered a region as having a high score if its *E* value was less than a cutoff. In keeping with Tapsin et al., we used a cutoff of *E*=10 for tests with small datasets. We varied the *E* value cutoff for tests with simulated data. Finally, high-scoring regions were declared as DRRs if they contained at least one nucleotide with (a) Bonferroni-corrected *p*_*i*_<0.1 and (b) absolute value of the logarithm of fold change > 2.

### Implementation of RASA

We received scripts utilized by Mizrahi et al. for data analysis (correspondence via email) [20]. We extracted key steps from their scripts and implemented them in custom written scripts for the sake of computational efficiency and proper code organization. RASA accepts detection counts and local coverages for two groups of samples. In addition, it accepts the mean reactivity of a suitable ribosomal RNA in each sample. The latter information helps account for the normalization requirements for reactivities. To this end, Mizrahi et al. utilized the mean reactivity of 28S rRNA in their study on human SP data. For our tests with *S. cerevisiae* and simulated data, we used the mean reactivities of 25S rRNA in each sample. For the test with fluoride riboswitch data, we used the mean reactivity of the riboswitch in each sample.

Given the abovementioned information, we identified DRRs in two steps. We executed the first step (regression analysis) separately for each nucleotide. In this step, we fit two generalized mixed models (with logistic regression) to the sample-wise counts and the coverages while also accounting for variation in the mean reactivities of samples. The null model assumed no effect of grouping of the samples. It attempted to explain the variation in detection rates from one sample to another as inherent biological variation. The alternative model considered the possibility of differential signal between the groups in addition to the biological variation. We compared the goodness of fit from the two models using a likelihood ratio test. In the presence of a real differential signal, the alternative model is expected to fit the data better. Hence, we summarized the output of the likelihood ratio test in terms of a *p* value to quantify the statistical significance of the improvement in the fit by the alternative model. In addition, the alternative model provided an assessment of the change in detection rates between the groups. If the *p* value for a nucleotide was < 0.01 and its absolute fold change in detection rates was > 1.33, the nucleotide was said to have a significant change in reactivity. We call such nucleotides as altered nucleotides.

In the second step, we searched for regions where altered nucleotides were clustered (spatial analysis). This step was executed separately for each RNA. We scanned an RNA in windows of a specified length. Mizrahi et al. used 50 nt as the window length. We used 50 nt for the *S. cerevisiae* rRNAs, 5 nt for the fluoride riboswitch data, and 11 nt for the simulated data (for justifications, see the relevant subsections of the “Results” section). Let the number of altered nucleotides in a window centered at nucleotide *i* be *w*_*i*_. We recorded two parameters for each transcript. The first parameter was the maximum value of *w*_*i*_. The second was the chi-square distance of the observed distribution of *w*_*i*_’s from their expected distribution in the absence of a differential signal. Specifically, in the absence of a differential signal, the *w*_*i*_’s should follow a Poisson distribution with the mean equal to the observed mean of the *w*_*i*_’s. Hence, we calculated the second parameter as the chi-square distance of the observed distribution and the expected Poisson distribution. In addition, we assessed both parameters for 1000 permutations of the observed arrangement of altered nucleotides. The permuted arrangements provided null values for the parameters. Next, we computed *Z* scores for each parameter value by comparing their observed values with the distribution of null values. Finally, we classified region(s) with the highest *w*_*i*_ and *Z*>2 for both parameters as DRRs.

A few more details are worth noting. In keeping with Mizrahi et al.’s implementation, we excluded nucleotides with untreated sample detection rates greater than 0.008 for As and 0.005 for Cs from the first step. We performed this filtering for both the real and the simulated Structure-Seq data. However, we skipped the filtering for the fluoride riboswitch as the cutoffs for untreated sample detection rates from SHAPE data were unknown. Moreover, due to the high quality of the fluoride riboswitch data, the untreated sample detection rates were generally low (median ∼ 0.002). In addition, if the local coverage at a nucleotide was greater than 10,000, we scaled down the local coverage to 10,000. For such nucleotides, we also scaled the detection count, such that the detection rate remained constant. The scaling was done to reduce the computational burden of performing regression analysis for each nucleotide separately.

### Simulations

We added simulated DRRs to experimentally obtained Structure-Seq data for three replicate samples of *S. cerevisiae*. To start with, we selected regions with lengths ranging from 50–75 nt out of 4681 mRNAs. We required that the selected regions have a minimum local coverage of > 25 and be among the top ∼ 20% of the mRNAs sorted according to average coverage. In addition, we allowed for more than one region in the same transcript. In total, out of the regions that satisfied the coverage criteria, we obtained 1000 regions in 630 mRNAs. Let us represent the selected regions as *R*_*i*_, with *i* ranging from 1 to 1000. For each selected region, we simulated three reactivity profiles, one labeled group A and the other two as two samples of group B. The typical way to simulate reactivity profiles given a secondary structure for a region is to sample reactivities randomly from probability density functions for reactivities of paired and unpaired nucleotides [54, 55]. However, such an approach results in zero correlation between replicates (data not shown). Hence, it does not result in realistic simulations as real data exhibits correlation within groups as well as between groups even in DRRs. Hence, we developed a new approach to simulate data for replicates, which displays a range of within-group and between-group correlations as well as between-group correlations. In what follows, we describe how we simulated reactivities and controlled the correlations within and between groups.

To simulate a reactivity profile for a region *R*_*i*_, first, we sampled 1000 secondary structures using its mRNA sequence as input to RNAsubopt (ViennaRNA package) [63]. We retained only the unique structures from those returned by RNAsubopt. In addition, we ensured that the MFE structure was represented in this set. Let us denote the generated structures for region *R*_*i*_ by *T*_*ij*_, where *j* ranges from 1 to the number of unique structures for *R*_*i*_. For each *T*_*ij*_, we generated a reactivity profile. To this end, we used patteRNA (with argument “-l”) to fit a Gaussian mixture model to our experimental data [33, 64]. Note that the fitting was done on the average reactivity profile from the three samples. Next, given a sequence of base pairing states for *T*_*ij*_, we sampled reactivities using the fitted model (we used scripts published with patteRNA for this purpose). Hence, for each region, we obtained a set of secondary structures and a reactivity profile for each structure. Let us denote the reactivity profile for structure *T*_*ij*_ as *r*_*k*,*ij*_, where *k* ranges from 1 to the length of *R*_*i*_. The final reactivity profile, denoted *r*_*k*,*i*_, for *R*_*i*_ for each sample was an ensemble-weighted average of *r*_*k*,*ij*_. Hence, we assigned each secondary structure an ensemble weight such that all ensemble weights summed to 1. For each *R*_*i*_, the *T*_*ij*_’s were divided into two categories—dominant structures (up to 5 in number) and infrequent structures. The dominant structures received a total ensemble weight between 0.33–0.66. The remaining ensemble weight was randomly distributed among the infrequent structures. Let us denote the ensemble weight for *T*_*ij*_ by *w*_*ij*_. Then, the reactivity profile of an *R*_*i*_ was obtained as $r_{k,i} = \sum _{j} w_{ij}r_{k,ij}.$

The three samples differed in the assignment of ensemble weights. We ensured that the two groups had different structure ensembles by ensuring that the sets of dominant structures for groups A and B are disjoint. Let us represent the *w*_*ij*_’s for groups A and B by *w*_*ij*,*A*_ and *w*_*ij*,*B*_, respectively. In addition, we ensured that the *r*_*k*,*i*_’s displayed a range of within-group and between-group variations as quantified in terms of within-group and between-group Pearson correlation coefficients. Note that the between-group correlation coefficient was obtained as the average of the correlation coefficients from comparing a sample from group A with two samples from group B. To ensure a range of within-group correlations, we added random noise to *w*_*ij*,*B*_ to represent two replicates from group B. The parameters and probability density functions for adding random noise were tuned by trial and error to ensure that a range of within-group correlations was obtained. In addition, to ensure a range of between-group correlations, we controlled the ensemble weight for the MFE structure in *w*_*ij*,*A*_ and *w*_*ij*,*B*_. Increasing the weight of the MFE structure in both groups to identical levels increased the between-group correlations. The parameters and probability density function dictating the selected level of MFE for an *R*_*i*_ were tuned based on a trial-and-error approach to ensure that a range of between-group correlations was obtained. Overall, we used five sets of parameters and probability density functions tuned by trial-and-error to obtain a range of within-group and between-group correlations for selected *R*_*i*_’s (Additional file 1: Figure S4). In keeping with the base-selective nature of DMS, reactivities for Gs and Us were masked as missing information in all simulated profiles. Then, *r*_*k*,*i*_’s for each sample were normalized using the 2–8% approach. After normalization, these *r*_*k*,*i*_’s replaced experimentally obtained reactivity profiles in the corresponding *R*_*i*_’s and samples. Let us represent the final reactivity for a transcript *t* as *r*_*k*,*t*_, where *k* ranges from 1 to the transcript’s length.

In addition to simulating reactivities, we needed counts and coverage information for running deltaSHAPE. Hence, we back-calculated count profiles that corresponded to *r*_*k*,*t*_’s. We preserved the experimentally observed hit rates of DMS on an mRNA (estimated as the sum of raw experimental reactivities [32]) for all mRNAs, their untreated detection rates/counts and local coverages in both the treated and untreated samples. With these pieces fixed, only the counts from the treated samples remained unknown. First, we estimated raw reactivities corresponding to *r*_*k*,*t*_’s. Let the hit rate of transcript *t* be *h*_*t*_. Then, raw reactivities for the transcript were obtained as $\nicefrac {r_{k,t}h_{t}}{\sum _{k} r_{k,t}}$. To these, we added the untreated sample detection rates to get the treated detection rates. Multiplying the treated detection rates and treated local coverages and rounding the result provided the treated sample counts. The back-calculated counts and local coverages were used along with the *r*_*k*,*t*_’s when running deltaSHAPE as described earlier.

dStruct, deltaSHAPE, PARCEL, and RASA were used to identify DRRs in the simulated data. We ran these for a range of parameter values more conservative as well as more liberal than the default parameters. For dStruct, we varied the minimum quality criterion for candidate regions, specified in terms of average *d*_within_. The maximum allowed value of average *d*_within_ ranged from 0.1 to 0.5. *d*_within_ of 0.1 and 0.5 correspond to mean SNR > 6 (stringent high quality criterion) and > 1 (very liberal quality criterion), respectively [37]. For deltaSHAPE, we varied the colocalization requirement for the number of screened nucleotides with a high reactivity change. At minimum, colocalization of two nucleotides (liberal criterion) within a search window of 11 nt was required to define a DRR, and we increased this requirement to up to six nucleotides (conservative criterion). For PARCEL, we varied the *E* value cutoff. Lower cutoffs amount to a more conservative criterion. The tested cutoff values ranged from 5 (conservative criterion) to 10000 (liberal criterion). For RASA, we varied the *Z* score cutoffs. Higher cutoffs amount to a more stringent criterion. The tested cutoff values ranged from 1 (liberal criterion) to 5 (conservative criterion).

### List of single-nucleotide variants for validation with PARS data

We obtained a list of single-nucleotide variants from the supplementary information provided by Wan et al. [9]. The list contained only those regions with variants (1907 in number), which were found to be riboSNitches by StrucDiff. Of these, 1576 variants were such that two cell lines out of the mother, father, and child trio were allelically identical. Note that none of these 1576 variants were independently validated by Wan et al. to be structure altering. We considered the PARS profiles of cell lines that were allelically identical for a variant as biological replicates for the 11 nt centered at the variable nucleotide. However, not all of the 1576 variants were unique. There were several duplicates of variants at the same genomic location. The duplicates corresponded to related transcripts, which were either splicing variants of the same gene or splicing variants of a gene and their fusion products with a neighboring gene. We verified that the counts in at least the 11 nt window centered at the variant for the related transcripts were exactly identical for all three cell lines. Hence, we collapsed all duplicates to a single variant. This resulted in 351 variants. While Wan et al.’s pairwise approach ensured that at least 2 of the 3 cell lines had high coverages for the reported variants, it did not ensure that all three had high coverage. Therefore, from the reduced set of 351 variants, we further filtered out variants that had average counts less than 10 in an 11-nt window around the variant, i.e., a variant at site *k* was excluded if $\frac {1}{11}{\sum \nolimits }_{i=k-5}^{k+5} \left (V1_{i} +S1_{i}\right) < 10$ for any of the 3 cell lines. In total, we retained 323 variants for our analysis.

### Implementation of StrucDiff

To the best of our knowledge, no software implementation of StructDiff is available. Hence, we implemented it as described by Wan et al. [9] using custom scripts. First, we smoothed the V1 and S1 counts using a rolling mean in windows of 5 nt. We obtained smoothed PARS scores, $\overline {r}_{i}$, for nucleotide *i* from the smoothed counts:

6 $$\begin{array}{@{}rcl@{}} \overline{r}_{i} = \log_{2} \left(\sum\limits_{j= i - 2}^{i+2} \frac{V1_{j} + 5}{5} \right) - \log_{2} \left(\sum\limits_{j= i - 2}^{i+2} \frac{S1_{j} + 5}{5} \right). \end{array} $$

Second, we calculated the absolute difference in the smoothed PARS scores, $\Delta \overline {r}_{i}$, between any pair of samples, say father (denoted with subscript *f*) and child (denoted with subscript *c*), as $\Delta \overline {r}_{i} = \left | \overline {r}_{i,f} - \overline {r}_{i,c} \right |$. Third, in terms of $\Delta \overline {r}_{i}$, we estimated the structural change score, *v*_SNV_, around a single-nucleotide variant at site *k* as:

7 $$\begin{array}{@{}rcl@{}}  v_{\text{SNV}} & = & \frac{1}{5} \sum\limits_{i = k-2}^{k+2} \Delta\overline{r}_{i}. \end{array} $$

Fourth, we assessed the statistical significance of the observed *v*_SNV_. To this end, we permuted the sequence of non-zero $\Delta \overline {r}_{i}$ values 1000 times. For each permuted sequence, we assessed a structural change score under the null hypothesis, *v*_null_, which we defined similarly to *v*_SNV_. A *p* value was estimated for a single-nucleotide variant as the fraction of *v*_null_ values greater than the corresponding *v*_SNV_. We used this implementation to estimate the *p* values for a subset of riboSNitches reported by Wan et al. The subset was selected by screening for variants that were (i) shared by at least 2 related transcripts, (ii) had identical V1 and S1 counts in an 11-nt window around a shared variant for the related transcripts, and (iii) were not classified identically by StrucDiff in the context of all related transcripts that shared the variant, i.e., in some contexts, the variant was classified as structure altering and in other contexts, it was classified as not structure altering.

## Additional file

Additional file 1 Supplementary information. Detailed overview of deltaSHAPE, PARCEL, RASA, StrucDif,f and classSNitch, supplementary figures and tables. The download links for all datasets used in this study and organization of data on Zenodo are described in **Table S2**. (PDF 11,040 kb)

## Abbreviations

DRR: Differentially reactive region
FDR: False discovery rate
MFE: Minimum free energy
SP: Structure profiling or structure probing
