# Supplementary material for: dStruct: identifying differentially reactive regions from RNA structurome profiling data
Source: Genome Biol. 2019 Feb 21;20:40. doi: 10.1186/s13059-019-1641-3 (PMC6385470; doi:10.1186/s13059-019-1641-3)
Supplement: Supplementary file 1 — Supplementary information. Detailed overview of deltaSHAPE, PARCEL, RASA, StrucDif,f and classSNitch, supplementary figures and tables. The download links for all datasets used in this study and organization of data on Zenodo are described in Table S2. (PDF 11,040 kb) [file 13059_2019_1641_MOESM1_ESM.pdf]

Supplementary Information for "dStruct: identifying differentially reactive regions from RNA structurome profiling data"

Krishna Choudhary, Yu-Hsuan Lai, Elizabeth J. Tran, Sharon Aviran

January 23, 2019

Contents

S1 deltaSHAPE **2**
S1.1 Overview . . . 2
S1.2 Limitations . . . 2

S2 PARCEL **6**
S2.1 Overview . . . 6
S2.2 Limitations . . . 6

S3 RASA **8**
S3.1 Overview . . . 8
S3.2 Limitations . . . 8

S4 StrucDiff **9**
S4.1 Overview . . . 9
S4.2 Limitations . . . 9

S5 classSNitch **12**
S5.1 Overview . . . 12
S5.2 Limitations . . . 12

List of Figures

S1 $\sigma/|\mu|$ is very sensitive to changes in mean. . . . 15
S2 dStruct uses a quality threshold. . . . 16
S3 dStruct identified sites of RNA-protein interactions. . . . 17
S4 Characteristics of simulated data. . . . 18
S5 Additional performance summaries from simulations. . . . 19
S6 dStruct's results correlate well with the ground truth in tests with simulated data. . . . 20
S7 riboSNitches reported by dStruct. . . . 21
S8 StrucDiff results are confounded by data quality. . . . 22
S9 All monotonic transformations result in the same relative ranks. . . . 23
S10 Arctan transformation. . . . 24
S11 d scores are related to Spearman correlation coefficient. . . . 25
S12 Differences of d_{between} and d_{within} for nearby nucleotides are uncorrelated. . . . 26
S13 Factors leading to high false discovery rate of deltaSHAPE. . . . 27
S14 deltaSHAPE is sensitive to outliers. . . . 29

List of Tables

S1 PCR primers and oligonucleotides used for Structure-Seq. . . . 30
S2 Download links for all datasets used. . . . 31

S1 deltaSHAPE

In this section, we briefly present an overview of deltaSHAPE and its limitations.

S1.1 Overview

Let us represent the reactivity of nucleotide i in groups A and B as $r_{A,i}$ and $r_{B,i}$ respectively. Note that in the following discussion, we use subscripts A and B to represent the groups A and B respectively and subscript i to represent a nucleotide with index i in a transcript. Given one reactivity profile for each group, deltaSHAPE first calculates the difference of reactivities, $\Delta r_i = r_{A,i} - r_{B,i}$ at each nucleotide. Next, it smoothes Δr_i by averaging over a 3 nt sliding window to obtain $\Delta \overline{r}_i$, i.e.,

$$\Delta \overline{r}_i = \frac{1}{3} \sum_{k=i-1}^{i+1} \Delta r_k. \tag{1}$$

Standard score, S_i is assesed for each nucleotide based on the mean and standard deviation of the distribution of $\Delta \overline{r}_i$ values for a transcript, which are denoted as $\mu_{\Delta \overline{r}}$ and $\sigma_{\Delta \overline{r}}$ respectively in the following equation, i.e.,

$$S_i = \frac{\Delta \overline{r}_i - \mu_{\Delta \overline{r}}}{\sigma_{\Delta \overline{r}}}. \tag{2}$$

Standard score captures the magnitude of reactivity change for a nucleotide in relation to the magnitude of changes across the transcript. Indeed, the rationale underlying deltaSHAPE is that stable RNA-protein interactions would have a strong effect on the reactivity of nucleotides. Hence, it utilizes standard score to identify nucleotides with largest changes in reactivity.

Besides the standard score, deltaSHAPE utilizes another screening criterion based on Z-factor to assess the local coverage level at a nucleotide [1]. If the local coverage at nucleotide i is high, r_i is derived from a large sample of reads mapping to i. Hence, standard error, SE_i of r_i should be low. SE_i can be assessed as standard deviation of sampling distribution for r_i using theoretical models. Indeed, it is inversely related to local coverage [2, 3]. Estimating Z-factor requires estimates of standard errors in smoothed reactivities, which are denoted as $\overline{\mathrm{SE}}_i$. The developers of deltaSHAPE implicitly assume that r_i's are independent to assess $\overline{\mathrm{SE}}_i$ in terms of the standard errors in unsmoothed reactivities.

$$\overline{\mathrm{SE}}_i = \frac{1}{3} \sqrt{\mathrm{SE}_{i-1}^2 + \mathrm{SE}_i^2 + \mathrm{SE}_{i+1}^2}. \tag{3}$$

Finally, Z-factor for nucleotide i, Z_i is calculated as

$$Z_i = 1 - \frac{1.96 \left(\overline{\mathrm{SE}}_{A,i} + \overline{\mathrm{SE}}_{B,i} \right)}{|\Delta \overline{r}_i|}. \tag{4}$$

In simple terms, Z_i assesses if the local coverage at a nucleotide is sufficiently high to exclude sampling variation as the explanation for observed $\Delta \overline{r}_i$. Depending on $\overline{\mathrm{SE}}_i$ and $\Delta \overline{r}_i$, Z_i can take negative values or, positive values less than 1. The developers implicitly assume that the sampling distribution of reactivities is normal (hence the factor of 1.96 in Eq. 4; for a normal distribution, 95% of area under the curve lies within 1.96 standard deviations of the mean). Under this assumption, positive values of Z_i indicate that the sampling distributions of reactivities in samples A and B do not have a significant overlap [1]. For well-covered sites, the sampling variation will be low. Furthermore, for such sites, $\overline{\mathrm{SE}}_i$ will be low leading to high Z_i. Hence, deltaSHAPE screens out substantially altered nucleotides as those that have $|S_i| \geq 1$ and $Z > 0$.

Under the null hypothesis, nucleotides that satisfy the $|S_i|$ and Z_i criteria should occur at random locations within a transcript. In other words, indices for such nucleotides should be independent random variables. Hence, if null hypothesis were true, it should be unlikely that several such nucleotides would be in close proximity of each other. However, sites interacting with proteins would manifest large reactivity changes at several closely located nucleotides. Hence, of the sites screened by $|S_i|$ and Z_i, if 3 or more nucleotides are colocalized in a 5 nt window (default settings), they are chained together and collectively reported as a single DRR [2].

S1.2 Limitations

While deltaSHAPE may identify DRRs if they have strong change in magnitudes of reactivities, it has several limitations. We list four of its limitations below.

1. deltaSHAPE eliminates inadequate coverage as a possible explanation for high $\Delta\bar{r}_i$ values. However, it does not consider or eliminate biological variation as it does not use replicates.

2. deltaSHAPE's results may be influenced by outliers in the distribution of the $\Delta\bar{r}_i$'s. This is because outliers can have a large effect on the sample mean and standard deviation, and thereby on the standard score of a $\Delta\bar{r}_i$. Hence, deltaSHAPE might report a different set of DRRs in presence of outliers than in their absence. To illustrate the impact of outliers, we considered reactivity profiles from *ex vivo* and in cell probing of 168 nt U1 snRNA (arbitrarily selected from the set of RNAs probed by Smola *et al.* [4]). We screened for DRRs in the pair of profiles using deltaSHAPE (default parameter settings) for three cases — (a) original profiles, (b) original profile altered by addition of three outliers with $|S_i| \geq 1$ but $Z < 0$ and, (c) original profile altered by addition of three outliers with $|S_i| \geq 1$ and $Z > 0$ (Fig. S14). We added the outliers at adjacent nucleotides in the 3' end of transcript where reactivity information was missing in original profiles. In addition, the outliers had Δr_i's comparable to the largest Δr_i's in the original profile. For comparison of original profiles, deltaSHAPE reported four DRRs. In both the cases with outliers, one of these regions with moderate Δr_i's was not reported. Furthermore, deltaSHAPE reported the region containing outliers when they had $|S_i| \geq 1$ and $Z > 0$. The values and proportion of outliers as well as the locations where they occur in the transcript would determine how different the set of DRRs might be. However, deltaSHAPE does not automatically filter outliers and guidelines on how to detect/filter outliers are lacking. Nevertheless, *ad hoc* outlier filtering has been performed before applying deltaSHAPE [2].

3. Given adequate coverage, deltaSHAPE is biased to report DRRs in any pair of samples. While we observed this behavior of deltaSHAPE in DMS probing data for *S. cerevisiae* rRNAs and simulations, we observed the same behavior of deltaSHAPE on SHAPE-MaP data. We downloaded data for two replicates each of SHAPE-MaP probing of *Xist* lncRNA under *ex vivo* and in-cell conditions [2]. On screening for DRRs between replicates of the same condition (i.e., null comparison), deltaSHAPE reported 177 DRRs (nucleotide-level FPR of 5.5 %) between replicates of in-cell probing. Similarly, it reported 185 DRRs (nucleotide-level FPR of 5.3 %) between replicates of *ex vivo* probing. Such a bias to always report DRRs can be understood by examining the criteria used by deltaSHAPE to call out DRRs. Below, we discuss the limitations of $|S_i|$, Z_i and colocalization criteria used by deltaSHAPE to show how they collectively lead to high FDR (Fig. S13).

   $|S_i|$ criterion. For any data, there will always be some nucleotides with $|S_i| \geq 1$ (Fig. S13A). This can be shown mathematically. For a transcript of length n,

$$\begin{aligned} \sigma^2_{\Delta\bar{r}} &= \frac{1}{n-1}\sum_{i=1}^{n}(\Delta\bar{r}_i - \mu_{\Delta\bar{r}})^2 \\ &< \frac{1}{n-1}\sum_{i=1}^{n}\left\{\max_i\left(|\Delta\bar{r}_i - \mu_{\Delta\bar{r}}|\right)\right\}^2 \\ &= \frac{n}{n-1}\left\{\max_i\left(|\Delta\bar{r}_i - \mu_{\Delta\bar{r}}|\right)\right\}^2 . \end{aligned} \tag{5}$$

   For unimodal distribution of $\Delta\bar{r}_i$, the proportion of data points with extreme values of $|\Delta\bar{r}_i - \mu_{\Delta\bar{r}}|$ will be small. Hence, from Eq. 5, $\sigma_{\Delta\bar{r}} \ll \max_i\left(|\Delta\bar{r}_i - \mu_{\Delta\bar{r}}|\right)$. In simple words, for any distribution, there will always be data points more than one standard deviation away from the mean. Therefore, there will be several nucleotides with $|S_i| \geq 1$ irrespective of presence/absence of DRRs. For example, this is the case for replicates of SHAPE-MaP data for *Xist* lncRNA (Fig. S13A).

   Z_i criterion. High observed values of $|S_i|$ could potentially be explained by low coverage and/or high biological variation. The Z-factor criterion checks for coverage and is a safeguard used by deltaSHAPE to prevent false positives. For each nucleotide, it compares the magnitudes of a theoretically estimated sampling variation and the observed reactivity difference. If the local coverage at a nucleotide is high, the corresponding sampling variation should be low. In such a case, the nucleotide might have $Z > 0$ indicating that sampling variation might not be sufficient to explain the observed reactivity difference. However, the observed difference might be explained by biological variation, which Z-factor does not account for. In addition, this criterion is easily met by well-covered nucleotides in good quality experiments that aim for high sequencing depth (Fig. S13B-C). Hence, Z-factor might be inadequate to prevent false discoveries by deltaSHAPE. Note that Smola *et al.* applied deltaSHAPE to identify DRRs in highly covered transcripts (median local coverage >5000) [4]. Hence, the DRRs identified by them should not be a consequence of sampling variation.

   Colocalization criterion. While we find that deltaSHAPE is biased to screen nucleotides based on $|S_i|$ and Z_i for any comparison, the screened nucleotides might not be colocalized. In such case, they

would not be reported by deltaSHAPE. However, the step of data smoothing artificially introduces correlations in $|S_i|$ of neighboring nucleotides (Fig. S13D-E). This enhances the likelihood that neighboring nucleotides will be screened and reported as DRRs. From Eq. 1-2,

$$S_{i+1} = S_i + \frac{\Delta r_{i+2} - \Delta r_{i-1}}{\sigma_{\Delta \overline{r}}}. \tag{6}$$

Hence, if $S_i \geq 1$, the probability that $S_{i+1} \geq 1$,

$$P(S_{i+1} \geq 1 | S_i \geq 1) > P(\Delta r_{i+2} \geq \Delta r_{i-1}). \tag{7}$$

The amount by which $P(S_{i+1} \geq 1| S_i \geq 1)$ exceeds $P(\Delta r_{i+2} \geq \Delta r_{i-1})$ depends on magnitude of S_i. Under the null hypothesis, both Δr_{i+2} and Δr_{i-1} have expected values of zero and symmetrical sampling distributions. Additionally, let us assume that their joint distribution, $f(x, y)$ is also symmetrical such that $f(x, y) = f(-x, -y)$ for all x and y. For example, this would be the case if Δr_{i+2} and Δr_{i-1} were independent or, if they were correlated such that their joint distribution had elliptical contours. Then, $P(\Delta r_{i+2} \geq \Delta r_{i-1})$ will be ≥ 0.5. This can be understood by examining the three possible relationships — Δr_{i+2} can be greater than, equal to or less than Δr_{i-1}. Of these, $\Delta r_{i+2} > \Delta r_{i-1}$ and $\Delta r_{i+2} < \Delta r_{i-1}$ are equally likely events [1]. Let $P(\Delta r_{i+2} > \Delta r_{i-1}) = P(\Delta r_{i+2} < \Delta r_{i-1}) = P_{\text{not equal}}$. Also, let $P(\Delta r_{i+2} = \Delta r_{i-1}) = P_{\text{equal}}$. Then,

$$\begin{aligned} P(\Delta r_{i+2} > \Delta r_{i-1}) + P(\Delta r_{i+2} < \Delta r_{i-1}) + P(\Delta r_{i+2} = \Delta r_{i-1}) &= 1 \\ \implies 2P_{\text{not equal}} + P_{\text{equal}} &= 1 \\ \implies P_{\text{not equal}} &= 1 - (P_{\text{not equal}} + P_{\text{equal}}) \end{aligned} \tag{8}$$

Since $P_{\text{equal}} \geq 0$,

$$\begin{aligned} P_{\text{not equal}} + P_{\text{equal}} &\geq P_{\text{not equal}} \\ \implies P_{\text{not equal}} + P_{\text{equal}} &\geq 1 - (P_{\text{not equal}} + P_{\text{equal}}) \text{ (using Eq. 8)} \\ \implies P_{\text{not equal}} + P_{\text{equal}} &\geq 0.5 \\ \implies P(\Delta r_{i+2} \geq \Delta r_{i-1}) &\geq 0.5. \end{aligned} \tag{9}$$

Note that above evaluation of probabilities makes two assumptions under the null hypothesis. First, we assume that for all i, Δr_i has expected value of zero and symmetrical sampling distribution. In addition, we assume that $f(x, y) = f(-x, -y)$ for all x and y. These assumptions should be valid if samples A and B are independent, have identical structures and identical coverages. Using Eq. 7 and Eq. 9,

$$P(S_{i+1} \geq 1| S_i \geq 1) > 0.5. \tag{10}$$

Similarly, it can be shown that $P(S_{i+1} \leq -1|S_i \leq -1) > 0.5$. With these probabilities in hand, we can examine the colocalization criterion. The colocalization criterion is implemented by deltaSHAPE with the rationale that sites of RNA-protein interactions should lead to substantial reactive changes at several closely located nucleotides. Indeed, if any isolated nucleotides manifest $|S_i| \geq 1$, they are rejected by deltaSHAPE given its underlying rationale. This rationale can be expressed in terms of a null hypothesis. The null hypothesis is that nucleotides with $|S_i| \geq 1$ are independently located, which makes it unlikely that several of them will be colocalized. Hence, the conditional probability, $P(|S_{i+1}| \geq 1| |S_i| \geq 1)$ should be $P(|S_{i+1}| \geq 1)$, regardless of whether $|S_i| \geq 1$. Moreover, if the distribution of $\Delta \overline{r}_i$'s is given, we know the proportion of nucleotides with $|S| \geq 1$, which is typically $\ll 0.5$. Under the null hypothesis, if a nucleotide is known to have $|S_i| \geq 1$, the probability that nucleotide $i + 1$ has $|S_{i+1}| \geq 1$ should be less than the proportion of nucleotides with $|S| \geq 1$ [2].

[1] If local coverage at nucleotide i is identical for samples A and B, $r_{A,i}$ and $r_{B,i}$ are independent and identically distributed under the null hypothesis. Hence, $r_{A,i}$ is equally likely to be greater or less than $r_{B,i}$. Moreover, Δr_i's have sampling distributions that are symmetrical with mean 0. Let us consider the joint distribution, $f(x, y)$ of Δr_{i+2} and Δr_{i-1} in 3D. If we assume that $f(x, y) = f(-x, -y)$ for all x and y, the plane $\Delta r_{i+2} = \Delta r_{i-1}$ divides the space in halves that correspond to $\Delta r_{i+2} > \Delta r_{i-1}$ and $\Delta r_{i+2} < \Delta r_{i-1}$. Due to symmetry, both halves should have equal volumes under the probability surfaces and hence, $P(\Delta r_{i+2} > \Delta r_{i-1}) = P(\Delta r_{i+2} < \Delta r_{i-1})$. Using a sampling experiment in R, we confirmed that two correlated/independent random variables, sampled from a normal distribution with mean 0 but arbitrary standard deviations and correlation satisfy this property (data not shown).

[2] An analogy might facilitate understanding of this point. Given the distribution of $\Delta \overline{r}_i$'s, we have two kinds of nucleotides — with $|S_i| \geq 1$ or, with $|S_i| < 1$. Let us say that these two kinds represent balls of two colors in an urn — blue and red, respectively. If we know the proportion of nucleotides with $|S| \geq 1$, we know the proportion of blue balls in the urn, which is say f_{blue}. If we pick a ball from the urn and it is blue, the probability that the next ball picked from the urn will be blue must be less than f_{blue}, i.e., if there is no bias.

However, as we have shown, if a nucleotide is found with $|S_i| \geq 1$, data smoothing introduces a bias that results in more than 50% chance of screening of additional nucleotides close to it. This bias is due to autocorrelation introduced in the data by data smoothing and, it will prevail regardless of presence/absence of real regional effects of a structure-altering factor.

Overall, the deficiencies of standard score and Z-factor as screening criteria and bias due to data smoothing lead to high FDR from deltaSHAPE.

4. deltaSHAPE tends to report only short stretches of a transcript as DRR as we observed in our simulations (Fig. S5E), irrespective of the true extent of DRRs. This is because deltaSHAPE rejects all nucleotides that have $|S_i| < 1$, even if a majority of nucleotides in the DRRs have $|S_i| < 1$. While the rationale to do this is to screen only nucleotides that have highest change in reactivities, not all nucleotides in a DRR may have a large change in reactivities. For example, in our simulations of the ensemble of structures, for a given region, some of the nucleotides could be in a paired state for say, $\sim$80 % of the dominant structures in both groups, while they differ in their pairing state for only the remaining proportion. Such nucleotides might have modest changes in reactivities compared to the nucleotides that are always paired in one group and always unpaired in other group. However, deltaSHAPE might not identify the former category of nucleotides if they have $|S_i| < 1$. This effectively limits the length of a transcript reported as DRR by deltaSHAPE. In other words, by its very design, deltaSHAPE can report only a limited fraction of a transcript's length as DRRs. However, a majority of a transcript's length can possibly be DRRs. For example, transcripts can have multiple interaction sites with several promiscuous/nonspecific proteins or be altered globally by SNVs [5, 6, 7]. With little known about RNA structures and the field currently in exploratory phase, it might be inadvisable to limit discovery by method design.

S2 PARCEL

Tapsin *et al.* developed PARCEL for *de novo* discovery of RNA aptamers [8]. PARCEL is a combination of experimental and supporting computational approaches. Unless specified otherwise, we use PARCEL to refer to only the differential analysis method utilized by Tapsin *et al.*. An overview and limitations of PARCEL are given below.

S2.1 Overview

PARCEL has four steps. These steps are executed for each RNA separately. In the first step, counts from multiple samples of an RNA are compared using edgeR [9]. edgeR is a popular software package for differential gene expression analysis. To adopt edgeR for comparing reactivity data, PARCEL considers each nucleotide as an equivalent of a "gene" or "genomic region" in edgeR model. In addition, it considers detection counts from only the reagent-treated samples. It utilizes the detection counts for a nucleotide as the equivalent of reads mapped to a gene in edgeR-based differential gene expression analysis. The samples of groups A and B are fed to edgeR. This yields a table containing p-values for each nucleotide and the corresponding fold changes between groups. Let the p-value for nucleotide i be p_i.

In the second step, p_i's are converted to scores,

$$s_i = \log(0.1) - \log(p_i). \tag{11}$$

The scores are inversely related to p_i's. s_i is positive when $p_i < 0.1$. If $p_i > 0.1$, s_i is negative indicating that the change in detection counts is not statistically significant. Nucleotides with low detection counts (< 1) are assigned a score of -10.

In the third step, PARCEL searches for regions that have high aggregate scores. If a region stretches from nucleotide j to k, its aggregate score, $S = \sum_{i=j}^{k} s_i$. To classify a region as high-scoring, a threshold value is needed for S. To this end, PARCEL adapts an approach developed by Karlin and Altschul and meant originally for assessing statistical significance of molecular sequence features [10]. In keeping with the model of Karlin and Altschul, PARCEL models the expected number, E of regions with score greater than or equal to S as

$$E = Ke^{-\lambda S}, \tag{12}$$

where K and λ are constants. PARCEL requires $E < 10$ for a region to be classified as high-scoring. Eq. 12 implies that the higher the aggregate score, the fewer will be the regions with a higher or equal score, that can be expected to occur by random chance. To evaluate E, values of K and λ are needed. Tapsin *et al.* derived values of K and λ based on the assumption that most nucleotides in a transcript do not undergo any structural changes. These values are $K = 0.0809635$ and $\lambda = 0.862871$. Given the values of K, λ and Eq. 12, $E < 10$ implies that aggregate score of a high-scoring region must be greater than -5.58. PARCEL uses Kadane algorithm to identify high scoring regions [11]. In our implementation, we utilized a recursive execution of Kadane algorithm to identify the regions with $S > -5.58$. Our execution scheme for any transcript is as follows:

1. Find the region with highest aggregate score using Kadane algorithm (available as a function 'maxsub' in R package 'adagio' [12]).
2. If the aggregate score for region found in step 1 is less than -5.58, terminate the search and report that no regions were found. Otherwise, report the region found in step 1 and repeat steps 1-2 for the segments to the left and right of region found in step 1.

In the final step, out of all the regions reported by Kadane algorithm, only those that satisfy the following criteria are called DRRs. The criteria are length greater than 1 nt and, at least 1 nucleotide with absolute fold change (reported by edgeR) > 2 and Bonferroni-corrected $p_i < 0.1$.

S2.2 Limitations

1. PARCEL was developed specifically to identify DRRs from PARCEL experimental data. The experimental component of PARCEL does not involve untreated samples. Hence, PARCEL does not provide a way to include the information in untreated samples for differential analysis. Nevertheless, the untreated sample is an integral component of most structure profiling technologies, for example, SHAPE-Seq, Structure-Seq, etc. [13, 14] Untreated samples are utilized to account for detection counts contributed by background noise. For example, such noise could be caused by random terminations of reverse transcription before reaching a modified nucleotide, degraded RNA

molecules, etc. While PARS does not utilize untreated samples, it involves probing of samples with two nucleases in separate experimental channels [15]. The reactivity information from PARS is obtained by combining counts information from both nucleases. However, PARCEL does not provide a way to combine information from the two nucleases either. Hence, it is limited in scope to technologies with only a single type of reagent and no untreated samples.

2. Even if one considers information from treated samples only, PARCEL does not account for variation in local coverages within a transcript. This is due to its dependence on edgeR to assess significance of changes at nucleotides. Generally, local coverage variation within a transcript may not be of significant concern in gene expression studies, which are the focus of edgeR. Indeed, typical gene expression studies concern with counts at the level of a transcript (or genomic region) and not individual nucleotides [16]. Hence, edgeR does not account for such variation. Yet, significant variation in coverages has been noted in RNA-seq experiments [17]. Moreover, such variation is also observed in typical structurome profiling experiments. As structurome profiling concerns with nucleotide-resolution signal, the counts and local coverage information should be considered simultaneously to compute the signal [13, 14, 18]. For example, 2 detection counts out of a local coverage of 10 reads should not be considered the same as 2 detection counts out of a local coverage of 100 reads. Ignoring the local coverage information can lead to high false discovery rate due to the confounding effect of variation in coverage.

3. The edgeR model of how reads are generated in RNA-seq experiments does not capture the reagent treatment step, which is a key distinction between RNA-seq and RNA structurome profiling. Direct application of edgeR to structurome profiling data can result in inaccurate p-values.

4. PARCEL does not provide a way to account for variation in reagent hit rates from one sample to another [13]. Typical studies account for such variation by normalizing raw reactivities. Hence, even in the absence of local coverage variation, DRRs found by PARCEL could be an artifact of sample-to-sample variation in reagent hit rates.

5. To derive values of K and λ, Tapsin *et al.* assume that most nucleotides in any transcript do not undergo structural changes. However, as we highlighted earlier, a majority of a transcript's length can possibly be DRRs. For example, transcripts can have multiple interaction sites with several promiscuous/nonspecific proteins or be altered globally by SNVs [5, 6, 7]. With little known about RNA structures and the field currently in exploratory phase, it might be inadvisable to make a strong assumption about the extent of structural alterations. At this point, it should be noted that this assumption does not prevent PARCEL from reporting most of a transcript's length as DRR. For example, in our test with fluoride riboswitch data (Figure 4), PARCEL reported a 72 nt long DRR out of 100 nt. However, the statistical significance (E value) of the aggregate score of a region might be inaccurate.

S3 RASA

Mizrahi *et al.* developed an approach to identify DRRs from DMS-MapSeq data for human foreskin fibroblast cells [19]. They described their approach under the title “Regression and spatial analysis”. For convenience, we acronymize it as RASA. An overview and limitations of RASA are given below.

S3.1 Overview

RASA has two steps, which are performed separately for each RNA. The goal of first step is to identify nucleotides with significant changes. Hence, it is performed once for each nucleotide. RASA excludes nucleotides with detection rates in the untreated samples greater than a cutoff value. Mizrahi *et al.* determined different cutoffs for As and Cs — 0.008 and 0.005, respectively, by optimizing the structure-prediction accuracy for 28S rRNA. Besides filtering based on untreated detection rates, RASA excludes nucleotides with local coverage < 10 in any of the samples or, with non-zero detection counts in fewer than two samples. For each of the remaining nucleotides, RASA utilizes two generalized mixed models. Each model compares detection rates across treated samples. One of them is based on null hypothesis of no difference between groups. It attempts to explain sample-to-sample variation as a random effect of inherent biological variation across samples. The alternative model considers group label of a sample as an explanatory variable. The group labels qualitatively represent the states of structure-altering factor in the two groups. If the two groups have differential structure, then there should be a significant fixed effect of the structure-altering factor on the detection rates. In addition to biological variation and structure-altering factor, both models consider variation in reagent hit rate as an additional source of sample variance in detection rates. To this end, RASA uses the mean reactivity of 28S rRNA in a sample as an offset in the models. The alternative and null models are compared using likelihood ratio test. This yields a p-value and effect size for each nucleotide. If the p-value is less than 0.01 and the absolute value of effect size is greater than 0.3, then the nucleotide is considered as significantly changing.

In the second step, RASA identifies regions where significantly changing nucleotides are clustered. To this end, it uses two parameters for each RNA, both based on the number of significantly changing nucleotides in sliding windows of 50 nt. Let the number of significantly changing nucleotides in window centered at nucleotide i be w_i. Then, the first parameter is the maximum value of w_i. The second parameter is the Chi square distance of the observed distribution of w_i's and the expected distribution of w_i's. The expected distribution is obtained under the assumption that w_i's are Poisson distributed with the same mean as the observed distribution. A null distribution for each parameter is obtained by permuting the nucleotides 1000 times and computing null values for the paramters in each iteration. The observed values of parameters are compared with corresponding null distributions to obtain standard scores. If the standard scores are greater than 2 for both parameters, then the transcript is classified as differentially structured between groups. Region(s) with maximum w_i are called DRR(s).

S3.2 Limitations

1. RASA was developed in conjunction with DMS-MapSeq data. Such data have been found to provide better estimates of reactivities from treated samples only than from a combination of treated and untreated samples [20]. However, in general, structurome profiling utilizes untreated samples to improve reactivity estimates by accounting for background noise. Indeed, a common practice is to assess structural signal or reactivity as the difference of detection rates in treated and untreated samples [13]. Moreover, filtering out nucleotides with higher-than-cutoff detection rates could substantially limit power, especially in transcriptome-wide *in vivo* probing data. Importantly, the detection rates in treated samples could be sufficiently high to assess structural signal even for nucleotides with high untreated detection rates. However, RASA does not provide a way to directly integrate information from untreated samples.

2. RASA does not address the problem of multiple testing. Since hypothesis tests are not free of error, simultaneous testing of thousands of RNAs in a transcriptome-wide study increases the likelihood of falsely rejecting the null hypothesis for at least some RNAs [21]. This can result in false discoveries. Hence, it is recommended that specialized methods be used to control false discoveries in a multiple testing scenario. However, RASA classifies nucleotides as significantly changing in first step and regions as DRRs in second step without performing multiple testing corrections in either step.

3. RASA filters nucleotides in the first step of analysis. The filtering is based on cutoff values for detection rates in untreated samples. The cutoff values were determined by optimizing the structure-prediction accuracy for 28S rRNA. These values might not be optimal for all studies.

S4 StrucDiff

The custom scripts that we used to implement StrucDiff are available online. In this section, we briefly present an overview of this method and its limitations.

S4.1 Overview

StrucDiff has five steps. Let us denote the PARS score of nucleotide i with r_{ij}, where j is m, f or c for mother, father or child, respectively. First, V1 and S1 counts are locally smoothed using rolling mean in sliding windows of 5 nt to calculate smoothed PARS scores, denoted as $\overline{r}_{ij}$,

$$\overline{r}_{ij} = \log_2\left(\sum_{l=i-2}^{i+2} \frac{V1_{lj}+5}{5}\right) - \log_2\left(\sum_{l=i-2}^{i+2} \frac{S1_{lj}+5}{5}\right). \tag{13}$$

In the second step, the absolute difference in smoothed PARS scores, $\Delta\overline{r}_i$ between any pair of samples, say $j = f$ and $j = c$, is calculated as $\Delta\overline{r}_i = |\overline{r}_{i,f} - \overline{r}_{i,c}|$. Third, in terms of $\Delta\overline{r}_i$, the structural change score, v_{SNV} around a SNV at site k is calculated as

$$v_{\text{SNV}} = \frac{1}{5}\sum_{i=k-2}^{k+2} \Delta\overline{r}_i. \tag{14}$$

In the fourth step, StrucDiff assesses statistical significance of observed v_{SNV}. To this end, it permutes the sequence of non-zero $\Delta\overline{r}_i$ values 1000 times (for the corresponding transcript). For each permuted sequence, it assesses a structural change score under null hypothesis, v_{null} defined similarly as v_{SNV}. A p-value is obtained for structure alterations in the SNV-containing region as the fraction of v_{null} values greater than v_{SNV}. In addition, StrucDiff controls FDR using Benjamini-Hochberg procedure. Finally, a SNV-containing region is classified as riboSNitch if it has $p < 0.05$, $q < 0.1$, $v_{\text{SNV}} > 1$ as well as high local coverage and signal strength (checked separately) in an 11 nt window around the SNV.

S4.2 Limitations

1. StrucDiff accounts for local coverage and signal strength only and eliminates these factors as a possible explanation for observed v_{SNV}. However, it does not eliminate biological variation as it does not use replicates.

2. Its scope is limited to guided discovery as it requires predefined regions for which v_{SNV} may be assessed. In the absence of predefined regions, it does not automatically construct these regions.

3. Since reactivity data manifest substantial noise at nucleotide-level, StrucDiff performs local smoothing of data to reduce noise. However, data smoothing might be done only at the expense of another issue. Due to local smoothing of data prior to estimating v_{SNV} and v_{null}, all pairs of $\Delta\overline{r}_i$'s do not have equal covariances under the null hypothesis, which violates the assumption of exchangeability for permutation tests [3]. This leads to different variances for v_{SNV} and v_{null} as shown below.

   While local smoothing is implemented on the V1 and S1 counts, for simplicity, let us assume smoothed PARS scores,

   $$\overline{r}_{ij} = \frac{1}{5}\sum_{l=i-2}^{i+2} r_{lj}. \tag{15}$$

   Additionally, between any pair of samples, say $j = f$ and $j = c$, let $\Delta\overline{r}_i = \overline{r}_{i,f} - \overline{r}_{i,c}$ and the difference in unsmoothed PARS scores, $\Delta r_i = r_{i,f} - r_{i,c}$. Then, from Eq. 15,

   $$\Delta\overline{r}_i = \frac{1}{5}\sum_{l=i-2}^{i+2} \Delta r_l. \tag{16}$$

   In applying permutation test, the developers implicitly assume that $\Delta\overline{r}_i$'s are exchangeable, i.e., the joint distribution of $\Delta\overline{r}_i$'s does not change under permutation of the subscripts [22]. As we noted earlier, if this assumption were valid, all pairs of $\Delta\overline{r}_i$'s would have equal covariances under

[3] "Permutation tests rely on the assumption of *exchangeability*, that is, under the hypothesis, the joint distribution of the observations is invariant under permutations of the subscripts. [For example] Observations are exchangeable if they are independent, identically distributed (i.i.d.), or if they are jointly normal with identical covariances." — Good, 2002 [22]

the null hypothesis. Let us assume that under the null hypothesis, Δr_i's are independent. We shall consider the impact of local correlations among Δr_i's later.

Since $\Delta \overline{r}_i$'s are obtained by local smoothing, $\Delta \overline{r}_i$ for a site has non-zero covariances with 4 neighboring sites on each side but no covariance with distant sites because smoothing is done locally. Hence, permutation test is inappropriate for locally smoothed data. Moreover, if $\Delta \overline{r}_i$'s were exchangeable, distribution of v_{null} values would represent the sampling distribution of v_{SNV} under the null hypothesis. If this were the case, v_{SNV} and v_{null} would have equal variances. Below, we show that this is not the case. Let the variance of Δr_i be σ_i^2. We derive expressions for v_{SNV} and v_{null} for two cases — once assuming that σ_i^2's are not equal and next, assuming that they are all equal to σ^2.

Under the assumption of independence of Δr_i's, the variance of v_{SNV} can be derived as,

$$\begin{aligned}
\sigma_{\text{SNV}}^2 &= Var\left(\frac{1}{5}\sum_{i=k-2}^{k+2}\Delta\overline{r}_i\right) \qquad \text{(using definition of } v_{\text{SNV}} \text{ in Eq. 14)}\\
&= Var\left(\frac{1}{25}\sum_{i=k-2}^{k+2}\sum_{l=i-2}^{i+2}\Delta r_l\right) \qquad \text{(using Eq. 16)}\\
&= \frac{1}{625}Var\left(\Delta r_{k-4}+2\Delta r_{k-3}+3\Delta r_{k-2}+4\Delta r_{k-1}+5\Delta r_k\right.\\
&\qquad\left.+4\Delta r_{k+1}+3\Delta r_{k+2}+2\Delta r_{k+3}+\Delta r_{k+4}\right)\\
&= \begin{cases} \frac{1}{625}\left(\sigma_{k-4}^2+4\sigma_{k-3}^2+9\sigma_{k-2}^2+16\sigma_{k-1}^2+25\sigma_k^2+16\sigma_{k+1}^2\right.\\ \qquad\left.+9\sigma_{k+2}^2+4\sigma_{k+3}^2+\sigma_{k+4}^2\right), & \text{if } \sigma_i^2 \neq \sigma_j^2 \text{ for all } i,j\\ \frac{17}{125}\sigma^2, & \text{if } \sigma_i^2 = \sigma^2 \text{ for all } i.\end{cases}
\end{aligned} \qquad (17)$$

This is not the same as the variance of v_{null} as we show below. To derive σ_{null}^2, let us assume that an iteration of permutation results in assigning $\Delta\overline{r}_i$ for arbitrary sites i_1, i_2, i_3, i_4, i_5 to sites $k-1, k-2, ..., k+2$ respectively. Then,

$$v_{\text{null}} = \frac{1}{5}\sum_{p=1}^{5}\Delta\overline{r}_{i_p}. \qquad (18)$$

σ_{null}^2 can be obtained approximately by assuming that i_1, i_2, i_3, i_4, i_5 are well-separated, i.e., the minimum separation between these sites is greater than 4 nt ($\Delta\overline{r}_i$'s borrow information from 2 nucleotides to each side; for our purpose, two sites are well-separated if there is no overlap in the nucleotides that they borrow from). Note that this is highly likely for a permuted sequence for general transcript lengths.

$$\begin{aligned}
\sigma_{\text{null}}^2 &= Var\left(\frac{1}{5}\sum_{p=1}^{5}\Delta\overline{r}_{i_p}\right) \qquad \text{(using definition of } v_{\text{null}} \text{ in Eq. 18)}\\
&= Var\left(\frac{1}{25}\sum_{p=1}^{5}\sum_{l=i_p-2}^{i_p+2}\Delta r_l\right) \qquad \text{(using Eq. 16)}\\
&= \begin{cases}\frac{1}{625}\sum_{p=1}^{5}\sum_{l=i_p-2}^{i_p+2}\sigma_l^2, & \text{if } \sigma_i^2 \neq \sigma_j^2 \text{ for all } i,j\\ \frac{1}{25}\sigma^2, & \text{if } \sigma_i^2 = \sigma^2 \text{ for all } i.\end{cases}
\end{aligned} \qquad (19)$$

If σ_i^2's were not equal, Eq. 17 and 19 show that v_{SNV} and v_{null} have unequal variances. Furthermore, even if $\sigma_i^2 = \sigma^2$ for all i, the variances of v_{SNV} and v_{null} are unequal. In simple terms, due to local smoothing, each $\Delta\overline{r}_i$ has a non-zero covariance with the $\Delta\overline{r}_i$ for 4 nucleotides flanking it. This covariance is reflected in the variance of v_{SNV}. However, after permuting the data, $\Delta\overline{r}_i$ for i_1, i_2, i_3, i_4, i_5 come from different locations within the transcript and hence, should not have any covariance. Therefore, v_{null} has a lower variance than v_{SNV}. Such unequal variances have been found to result in inflated error rates because the permutation test results can be confounded by unequal variances of v_{null} and v_{SNV} [23]. Indeed, due to larger variance of v_{SNV} than v_{null}, v_{SNV} can take extreme values just by chance, leading to increased FDR or decreased power [24] [4].

[4]Readers that lack background in statistics might find it helpful to grasp this point with a familiar example. Let us say a t-statistic is assessed from 6 data points that are independent observations of a physical quantity. If the goal were to

Note that above derivations assume that Δr_i's are independent. This may not be the case if Δr_i's depend on mean reactivities at corresponding nucleotides. Since reactivities might manifest correlations between nearby nucleotides, Δr_i's might also manifest local correlations, thereby violating independence. We can relax the independence assumption for Δr_i's by adding local covariance terms to Eq. 17 and 19. The local covariance terms would be different for σ^2_{SNV} and σ^2_{null}. For σ^2_{SNV}, local covariance terms would depend on local correlations in Δr_i's around site of SNV, i.e., for nucleotides $k-4$ to $k+4$. However, for σ^2_{null}, there would be separate terms for local correlations around sites i_1, i_2, i_3, i_4 and, i_5. Importantly, the local covariance terms for σ^2_{SNV} and σ^2_{null} might not be identical in general. Hence, local correlations in Δr_i's might enhance the difference between σ^2_{SNV} and σ^2_{null}.

Above derivations of variances for v_{null} and v_{SNV} were based on a simplified expression for $\overline{r}_{ij}$, which involved local smoothing at the level of reactivities. In addition, besides the magnitudes of reactivity differences, we considered their signs in above derivations. However, StrucDiff involves smoothing at the level of counts (Eq. 13) and utilizes absolute values of reactivity differences. We performed simulations to demonstrate that our conclusion about unequal variances of v_{null} and v_{SNV} is valid even if smoothing were done at the level of counts and absolute values of reactivity differences were used to compute v_{null} and v_{SNV}. To this end, we randomly sampled two pairs of 1000 nt long sequences of V1 and S1 counts. One pair was labeled sample A and the other as B. We assumed a Poisson distribution of counts with means for V1 and S1 counts randomly sampled from a uniform distribution between 10 and 100. Such sampling scheme resulted in independent and identically distributed counts for nucleotides in each sequence. The sampled counts were smoothed and $\overline{r}_{ij}$ was computed as described in Eq. 13. Next, we randomly selected 500 locations within the sequence, which were chosen to be SNV sites. We assessed p- and q-values for $\Delta\overline{r}_{ij}$ of each site using the StrucDiff approach. 9.4% of the 500 sites yielded significant result ($p < 0.05$, $q < 0.1$). Since the data was randomly generated, all sites should be identical and the significant results should be false positives. In addition, we recorded v_{SNV} for each site and computed 500 v_{null} values. Each v_{null} was mean of $\Delta\overline{r}_i$'s for 5 randomly sampled locations. We observed that σ^2_{SNV} was 3.1 times σ^2_{null}. Eq. 17 and 19 suggest that for local smoothing at reactivity level, $\sigma^2_{\mathrm{SNV}}/\sigma^2_{\mathrm{null}} = 3.4$ if Δr_i's are independent and identically distributed. Real data might have different relative magnitudes of σ^2_{SNV} and σ^2_{null}. Importantly, the two variances might not be equal because local correlations are introduced in data regardless of whether local smoothing is done at the counts level or at the reactivity level. It is worthwhile to note that on eliminating data smoothing in the above test, none of the 500 sites yielded significant p- and q-values and $\sigma^2_{\mathrm{SNV}}/\sigma^2_{\mathrm{null}}$ was 1.01.

4. Another issue with StrucDiff is in its approach to p-value estimation. Due to the finite number of permutations performed, the estimated p-values could be imprecise, understated and even 0 [25]. Such imprecise estimates in multiple testing context can result in inflated type I error rates [25].

5. For permutation test to be applicable, Δr_i need to be identically distributed under null hypothesis. However, Δr_i might not be identically distributed. In fact, Δr_i might depend on the local coverage and signal strength, or in general, data quality. Indeed, we found that StrucDiff method was confounded by data quality outside regions of interest. The impact of data quality on StrucDiff results could be understood with an example. We identified three related transcripts reported by Wan *et al.* – LY75 gene product and two splicing variants of naturally occurring readthrough transcription between neighboring LY75 and CD302 genes. They all shared a SNV located within the LY75 gene. In fact, the data mapped around the SNV was exactly the same for all three transcripts (Fig. S8). Yet, StrucDiff inconsistently classified the structural change for this SNV-containing region between father and child as not a riboSNitch for LY75 gene product and riboSNitch for the splicing variants of LY75-CD302 fusion product. Since the v_{SNV} was equal for all three transcripts, the anomaly in classification was due to different data quality for these transcripts outside the region of interest, which significantly impacted the v_{null} values. While we could identify this one example of anomaly due to the special relationship between transcripts affected by the anomaly, this example illustrates a general fact that conclusions from StrucDiff are confounded by quality of data outside regions of interest. We could not assess if this confounding factor significantly impacts StrucDiff's results as data quality varies unpredictably within a transcript due to priming biases [17].

test if the sample mean of observations is significantly different from 0, the test statistic should be compared to Student's t-distribution with 5 degrees of freedom instead of the standard normal distribution. This is because the test statistic is derived from 6 data points with population standard deviation unknown. Hence, its sampling distribution is given by t-distribution with 5 degrees of freedom, which is more spread out than the standard normal distribution. Importantly, comparing the test statistic with standard normal distribution would result in inflated error rate. For the same reason, under the null hypothesis, sampling distribution of v_{SNV} (test statistic in StrucDiff) should be well-represented by the distribution of v_{null} values.

S5 classSNitch

We could not compare performances of classSNitch and dStruct in discovering DRRs because the scope of classSNitch is limited to guided discovery and data from SHAPE-based structure probing. While SHAPE data for systems with predefined candidate regions are available, none to the best of our knowledge have replicate samples. In the absence of replicate samples, we could not run dStruct. Here, we provide a brief overview of classSNitch and discuss its limitations.

S5.1 Overview

classSNitch is based on the observation that human ability to detect changes in reactivity patterns is exceptional [26]. Indeed, in the field of RNA structure probing, researchers have traditionally relied on visual examination to identify DRRs. Hence, Woods *et al.* conducted an online survey in which expert users of SHAPE data were asked to classify a set of wild-type/mutant pairs of SHAPE profiles as unaltered between the strains, locally altered or globally altered [26]. They noted a high level of agreement in responses from different experts. Hence, they used the responses as ground truths for the set of wild-type/mutant pairs. This set of SHAPE profiles with class labels based on expert responses was used to train a random forest classifier (supervised learning). The classifier utilized 8 features (e.g., Pearson correlation coefficient, Euclidean distance, etc.) of a pair of SHAPE profiles for classification. The training data used by developers is available along with classSNitch software package and could be used to retrain the model before conducting comparative analyses on novel data.

S5.2 Limitations

1. A major limitation of classSNitch is that it does not account for biological variation as it does not use replicates. As such, its predictions might reflect inherent variability in candidate regions instead of real effect of a structure-altering factor.

2. classSNitch requires predefined candidate regions such that it can assess the set of 8 features for the given regions and use them for classification. In the absence of predefined regions, it does not automatically construct them.

3. The classification model used by classSNitch is trained with SHAPE data only. Hence, the decision boundaries learned by the model are specific to SHAPE reactivities. For example, classSNitch utilizes Euclidean distances between a pair of profiles as one of the features for classification. A pair with high Euclidean distance is more likely to be classified as altered. However, Euclidean distances between a pair of PARS profiles could be much larger than the distances between a pair of SHAPE profiles, even in the absence of any real alterations. This is because the former typically allows for a broader range of reactivities. Such dependence on allowed ranges presents an issue with interpreting results from classSNitch for data from structure probing methods that utilize reagents other than SHAPE. This issue could potentially be resolved by training classSNitch with a diverse set of training data and considering the reagent type as an additional feature relevant to classification. However, such a diverse training dataset is not currently available to the best of our knowledge.

References

[1] Ji-Hu Zhang, Thomas D. Y. Chung, and Kevin R. Oldenburg. "A Simple Statistical Parameter for Use in Evaluation and Validation of High Throughput Screening Assays". In: *Journal of Biomolecular Screening* 4.2 (1999), pp. 67–73. DOI: 10.1177/108705719900400206. URL: http://journals.sagepub.com/doi/abs/10.1177/108705719900400206.

[2] Matthew J. Smola et al. "SHAPE reveals transcript-wide interactions, complex structural domains, and protein interactions across the *Xist* lncRNA in living cells". In: *Proceedings of the National Academy of Sciences* 113.37 (2016), p. 10322. URL: http://www.pnas.org/content/113/37/10322.abstract.

[3] Krishna Choudhary et al. "Metrics for rapid quality control in RNA structure probing experiments". In: *Bioinformatics* 32.23 (2016), pp. 3575–3583. URL: http://dx.doi.org/10.1093/bioinformatics/btw501.

[4] Matthew J. Smola, J. Mauro Calabrese, and Kevin M. Weeks. "Detection of RNA–Protein Interactions in Living Cells with SHAPE". In: *Biochemistry* 54.46 (2015), pp. 6867–6875. DOI: 10.1021/acs.biochem.5b00977. URL: https://doi.org/10.1021/acs.biochem.5b00977.

[5] Solem Amanda C. et al. "The potential of the riboSNitch in personalized medicine". In: *Wiley Interdisciplinary Reviews: RNA* 6.5 (2015), pp. 517–532. DOI: doi:10.1002/wrna.1291. URL: https://doi.org/10.1002/wrna.1291.

[6] Eckhard Jankowsky and Michael E Harris. "Specificity and non-specificity in RNA–protein interactions". In: *Nature reviews. Molecular cell biology* 16.9 (2015), pp. 533–544. DOI: 10.1038/nrm4032. URL: http://www.ncbi.nlm.nih.gov/pmc/articles/PMC4744649/.

[7] Chen Davidovich et al. "Promiscuous RNA binding by Polycomb repressive complex 2". In: *Nature Structural &Amp; Molecular Biology* 20 (2013), 1250 EP –. URL: http://dx.doi.org/10.1038/nsmb.2679.

[8] Sidika Tapsin et al. "Genome-wide identification of natural RNA aptamers in prokaryotes and eukaryotes". In: *Nature Communications* 9.1 (2018), p. 1289.

[9] Mark D Robinson, Davis J McCarthy, and Gordon K Smyth. "edgeR: a Bioconductor package for differential expression analysis of digital gene expression data". In: *Bioinformatics* 26.1 (2010), pp. 139–140.

[10] Samuel Karlin and Stephen F Altschul. "Methods for assessing the statistical significance of molecular sequence features by using general scoring schemes". In: *Proceedings of the National Academy of Sciences* 87.6 (1990), pp. 2264–2268.

[11] Tadao Takaoka. "Efficient algorithms for the maximum subarray problem by distance matrix multiplication". In: *Electronic Notes in Theoretical Computer Science* 61 (2002), pp. 191–200.

[12] Hans Werner Borchers. *adagio: Discrete and Global Optimization Routines*. R package version 0.7.1. 2018. URL: https://CRAN.R-project.org/package=adagio.

[13] Krishna Choudhary, Fei Deng, and Sharon Aviran. "Comparative and integrative analysis of RNA structural profiling data: current practices and emerging questions". In: *Quantitative Biology* 5.1 (2017), pp. 3–24. DOI: 10.1007/s40484-017-0093-6. URL: https://doi.org/10.1007/s40484-017-0093-6.

[14] Sharon Aviran, Julius B Lucks, and Lior Pachter. "RNA structure characterization from chemical mapping experiments". In: *Communication, Control, and Computing (Allerton), 2011 49th Annual Allerton Conference on*. IEEE. 2011, pp. 1743–1750.

[15] Michael Kertesz et al. "Genome-wide measurement of RNA secondary structure in yeast". In: *Nature* 467 (2010), 103 EP –. URL: http://dx.doi.org/10.1038/nature09322.

[16] Alicia Oshlack, Mark D Robinson, and Matthew D Young. "From RNA-seq reads to differential expression results". In: *Genome Biology* 11.12 (2010), p. 220.

[17] Jun Li, Hui Jiang, and Wing Hung Wong. "Modeling non-uniformity in short-read rates in RNA-Seq data". In: *Genome Biology* 11.5 (2010), R50. DOI: 10.1186/gb-2010-11-5-r50. URL: https://doi.org/10.1186/gb-2010-11-5-r50.

[18] Alina Selega et al. "Robust statistical modeling improves sensitivity of high-throughput RNA structure probing experiments". In: *Nature Methods* 14.1 (2017), p. 83.

[19] Orel Mizrahi et al. "Virus-Induced Changes in mRNA Secondary Structure Uncover cis-Regulatory Elements that Directly Control Gene Expression". In: *Molecular Cell* (2018).

[20] Meghan Zubradt et al. "DMS-MaPseq for genome-wide or targeted RNA structure probing *in vivo*". In: *Nature Methods* 14 (2016), 75 EP –. URL: http://dx.doi.org/10.1038/nmeth.4057.

[21] Jelle J Goeman and Aldo Solari. "Multiple hypothesis testing in genomics". In: *Statistics in Medicine* 33.11 (2014), pp. 1946–1978.

[22] Phillip I Good. "Extensions of the concept of exchangeability and their applications". In: *Journal of Modern Applied Statistical Methods* 1.2 (2002), p. 34.

[23] Yifan Huang et al. "To permute or not to permute". In: *Bioinformatics* 22.18 (2006), pp. 2244–2248. URL: http://dx.doi.org/10.1093/bioinformatics/btl383.

[24] Keegan Korthauer et al. "Detection and accurate false discovery rate control of differentially methylated regions from whole genome bisulfite sequencing". In: *Biostatistics* (2018), kxy007–kxy007. URL: http://dx.doi.org/10.1093/biostatistics/kxy007.

[25] Belinda Phipson and Gordon K Smyth. "Permutation P-values should never be zero: calculating exact P-values when permutations are randomly drawn". In: *Statistical Applications in Genetics and Molecular Biology* 9.1 (2010).

[26] Chanin Tolson Woods and Alain Laederach. "Classification of RNA structure change by 'gazing' at experimental data". In: *Bioinformatics* 33.11 (2017), pp. 1647–1655. URL: http://dx.doi.org/10.1093/bioinformatics/btx041.

[27] Yun Bai et al. "RNA-guided assembly of Rev-RRE nuclear export complexes". In: *eLife* 3:e03656 (2014). URL: http://dx.doi.org/10.7554/eLife.03656.

[28] Krishna Choudhary et al. "dStruct: identifying differentially reactive regions from RNA structurome profiling data, Datasets." In: *Zenodo* (2019). DOI: 10.5281/zenodo.2536501. URL: https://doi.org/10.5281/zenodo.2536501.

[29] Eric J. Strobel et al. "Distributed biotin–streptavidin transcription roadblocks for mapping cotranscriptional RNA folding". In: *Nucleic Acids Research* 45.12 (2017), e109–e109. URL: http://dx.doi.org/10.1093/nar/gkx233.

[30] Yue Wan et al. "Landscape and variation of RNA secondary structure across the human transcriptome". In: *Nature* 505 (2014), 706 EP –. URL: http://dx.doi.org/10.1038/nature12946.

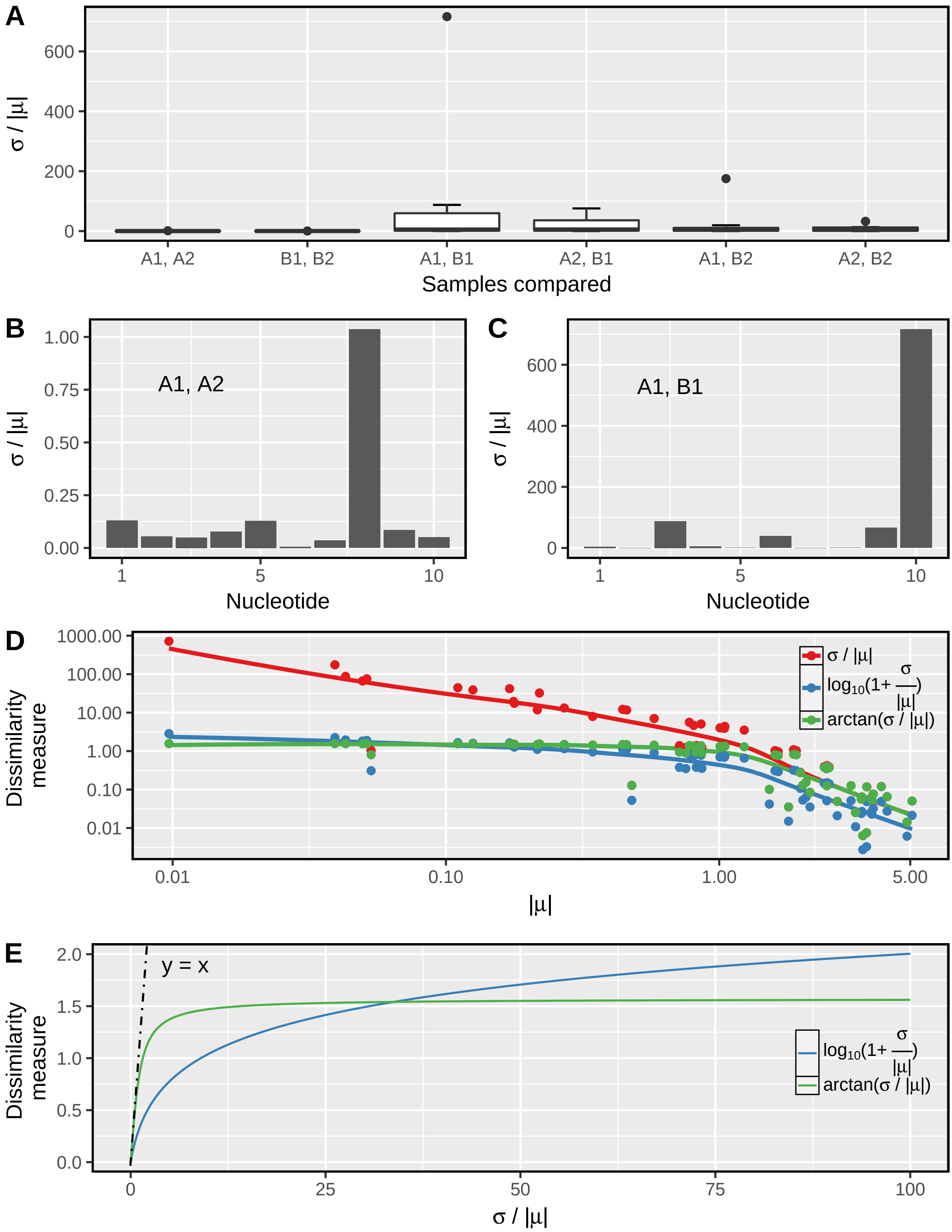

Fig. S1: **$\sigma/|\mu|$ is very sensitive to changes in mean. A.** Distributions of $\sigma/|\mu|$ for different pairwise comparisons of samples. Note that $\sigma/|\mu|$ can take very large values, especially for between-group comparisons. **B.** $\sigma/|\mu|$ for within-group pairwise comparison of samples A1 and A2. **C.** $\sigma/|\mu|$ for between-group pairwise comparison of samples A1 and B1. Note that the y-axis limits are different for **B** and **C** panels. **D-E.** Comparison of possible choices for dissimilarity measures. In **D**, three possible choices — untransformed $\sigma/|\mu|$, $\log_{10}(1+\sigma/|\mu|)$ and $\arctan(\sigma/|\mu|)$ are plotted against $|\mu|$. X- and Y-axes are plotted on log scale. $\sigma/|\mu|$ is calculated for all pairwise comparisons of samples A1, A2, B1 and B2 as shown in Figure 1 (main text). Note that $\sigma/|\mu|$ can be very high for small $|\mu|$. $\arctan(\sigma/|\mu|) \approx \sigma/|\mu|$ for $\sigma/|\mu| < 1$ (red and green curves overlap when Y-axis values are < 1). In addition, $\arctan(\sigma/|\mu|)$ is close to $\log_{10}(1+\sigma/|\mu|)$ for $\sigma/|\mu| < 1000$ (blue and green curves are close to each other in entire plotted range). In panel **E**, the dotted black line is the $y = x$ line. $\arctan(\sigma/|\mu|)$ asymptotically reaches $\pi/2$ as $\sigma/|\mu|$ increases, whereas $\log_{10}(1+\sigma/|\mu|)$ continues to increase with $\sigma/|\mu|$.

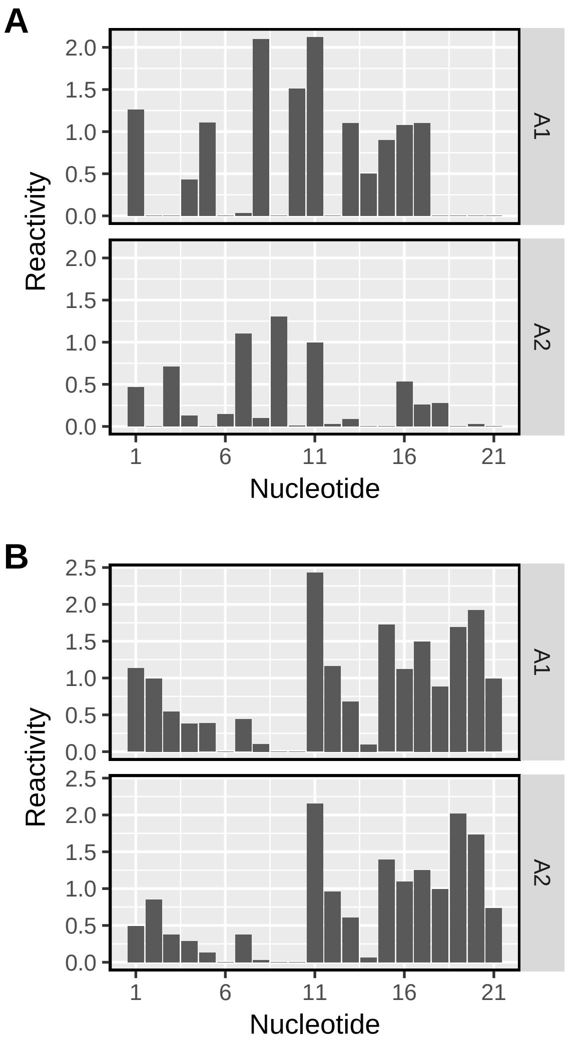

Fig. S2: **dStruct uses a quality threshold. A.** Example of a region with poor quality of agreement between replicates. This region has an average d score of 0.54. **B.** Example of a region with good quality of agreement between replicates. This region has an average d score of 0.13. Importantly, lower value of average d score corresponds to higher replicate agreement. Note that this is a simulated data for illustration purpose only.

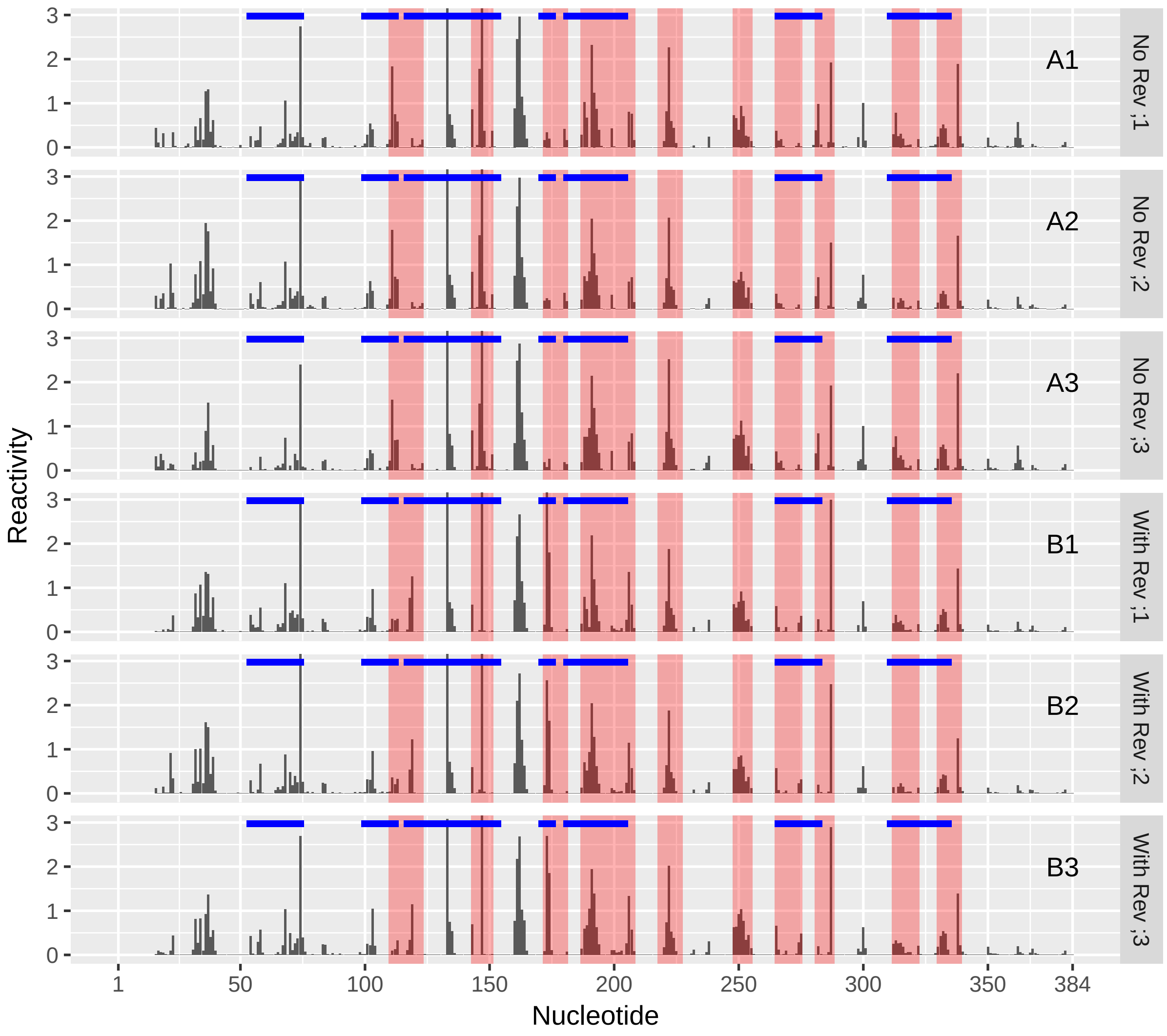

Fig. S3: **dStruct identified sites of RNA-protein interactions.** Plotted are 6 samples of reactivity profiles for HIV RRE in absence of Rev protein (samples A1, A2 and A3) and in its presence (samples B1, B2 and B3). Solid blue lines along top border of samples highlight regions known to interact with Rev proteins. Regions with red background highlight regions found to be DRRs by dStruct. Note that the ground truth of regional boundaries is only approximately available to the best of our knowledge. The extent of blue lines highlight the regions shown to interact with Rev in a schematic diagram by Bai *et al.*, 2014 [27].

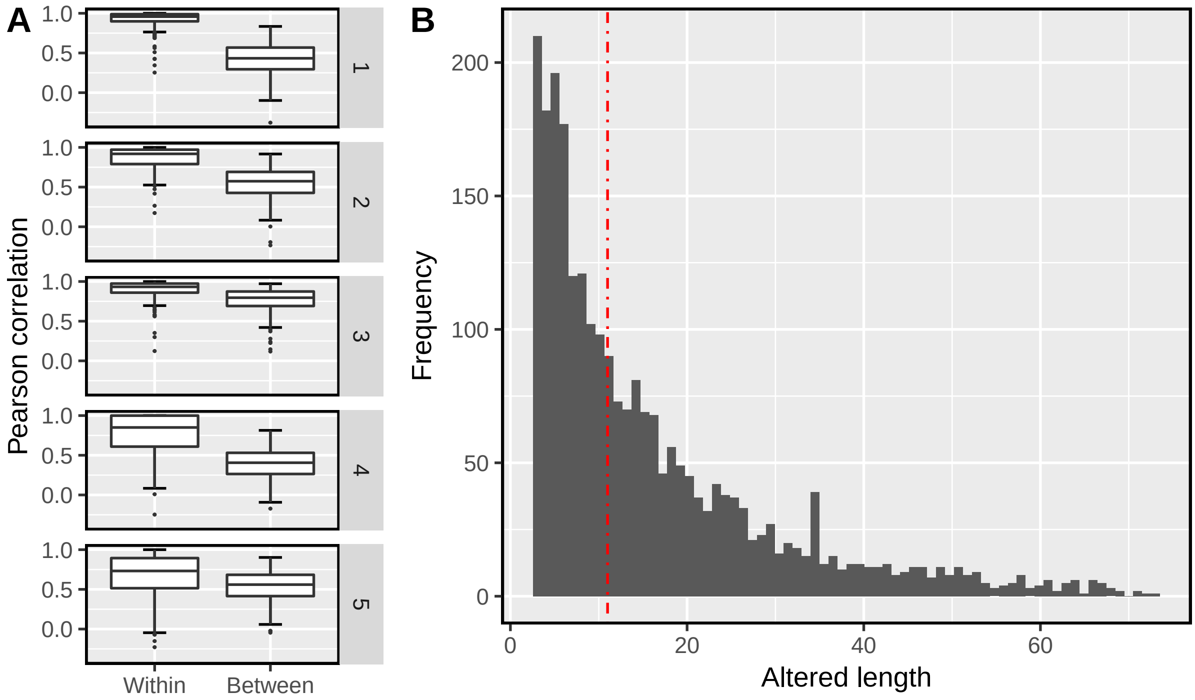

Fig. S4: **Characteristics of simulated data.** **A.** We tuned parameters in our simulation scheme such that plotted distributions of within-group and between-group Pearson correlation coefficients were obtained. In total, we used five sets of parameters, each for 200 regions. **B.** Distribution of lengths of contiguous stretches with altered base pairing states. Dashed vertical red line shows the median.

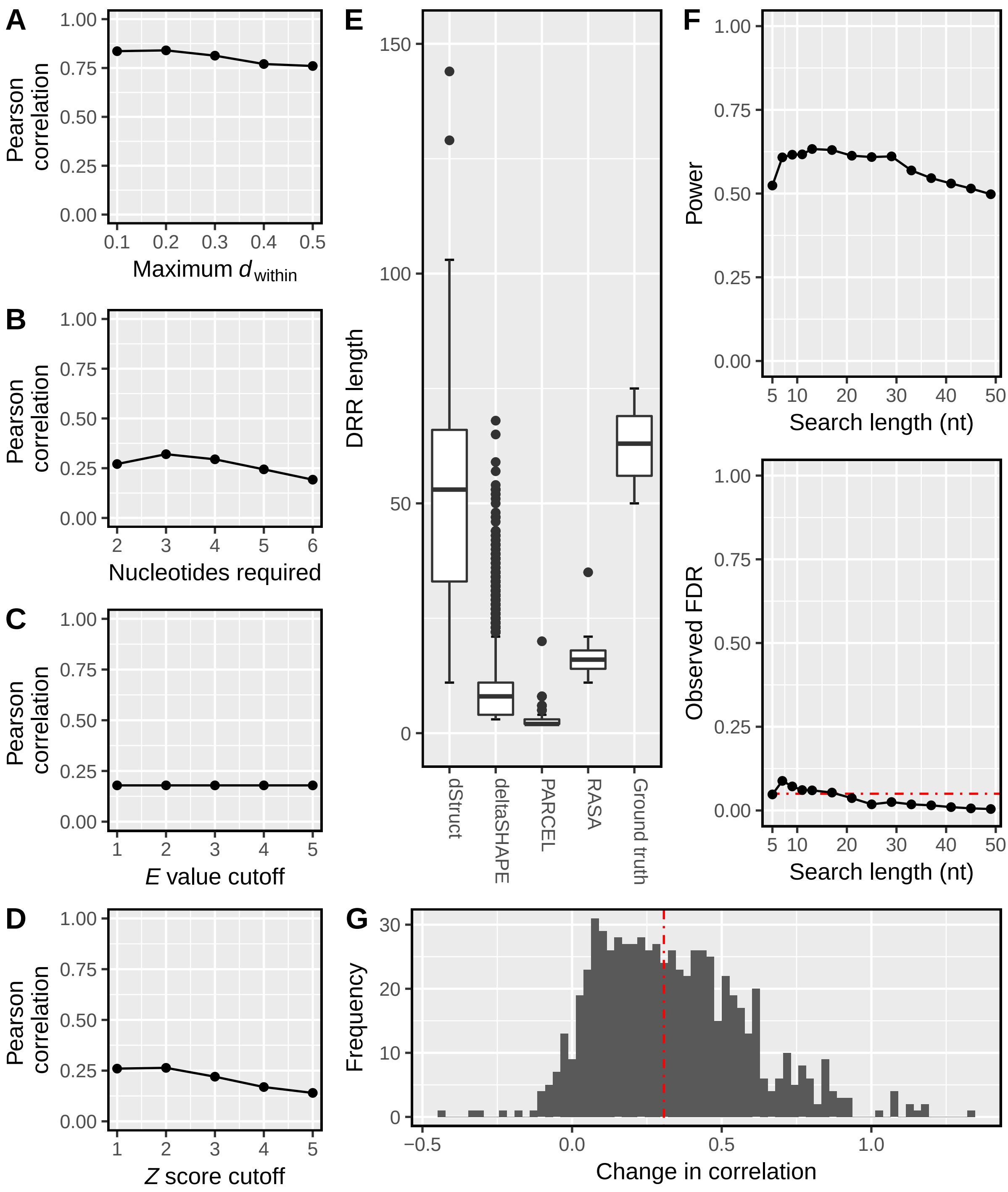

Fig. S5: **Additional performance summaries from simulations. A-D.** Correlation between the ground truths of fractions of transcript lengths that are real DRRs and the corresponding fractions reported as DRRs by **A.** dStruct on varying the minimum quality threshold, **B.** deltaSHAPE on varying the colocalization criterion, **C.** PARCEL on varying the E value cutoff and **D.** RASA on varying the Z score cutoff. Note that dStruct always correlated better with the ground truth. **E.** The distributions of DRR lengths as reported by dStruct, deltaSHAPE, PARCEL, RASA and that in the ground truth. Note that dStruct reports DRRs with almost the same median length as the simulated DRRs. **F.** Power and observed FDR of dStruct on varying the search length. Dashed red line indicates the specified FDR level. Note that dStruct has similar powers for all search lengths and the observed FDR is properly controlled to the level specified. **G.** We computed within-group and between-group Pearson correlation coefficients (see Methods) for the DRRs reported by dStruct. Plotted is the distribution of the difference of the within-group and between-group correlations in the DRRs. Positive values of the difference imply that reactivity patterns were more similar within groups and were altered between groups. Vertical dashed red line marks the median for the distribution. The distribution reveals that for the majority of DRRs reported by dStruct, the reactivity patterns were altered between groups.

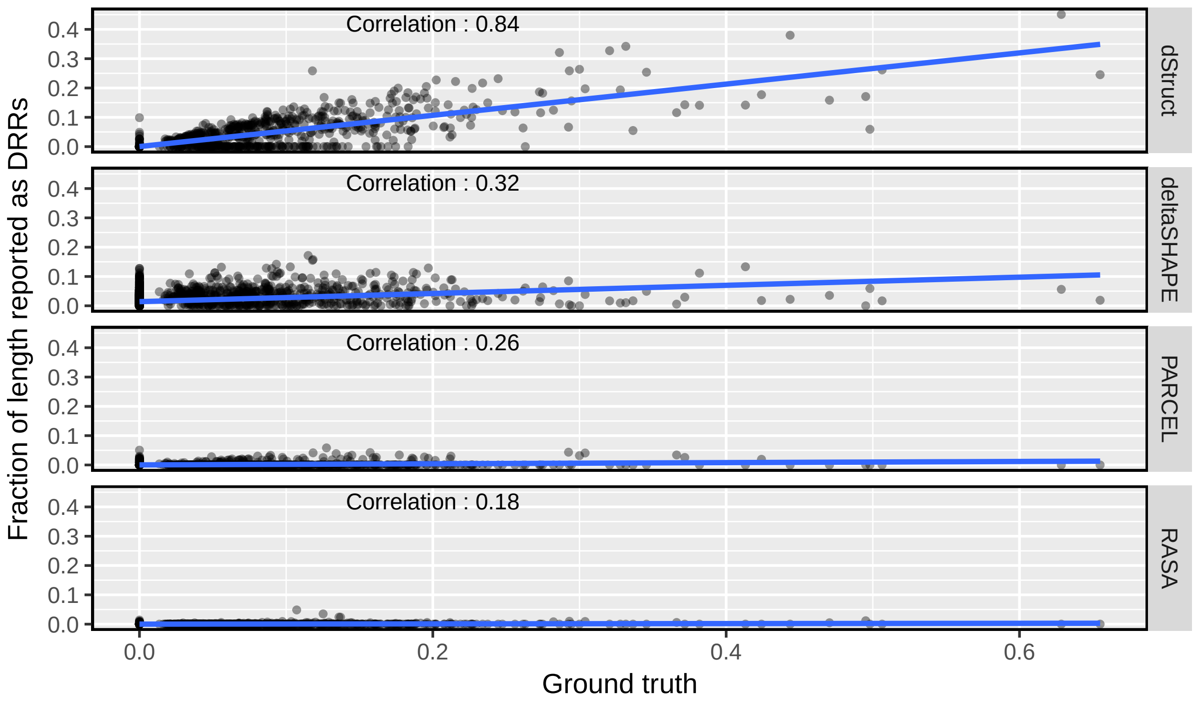

Fig. S6: **dStruct's results correlate well with the ground truth in tests with simulated data.** Fractions of lengths reported as DRRs by different methods are plotted against the ground truths of these fractions for dStruct, deltaSHAPE, PARCEL and RASA. The blue lines are linear fits to data points. Note that dStruct's results correlate the best with the ground truth.

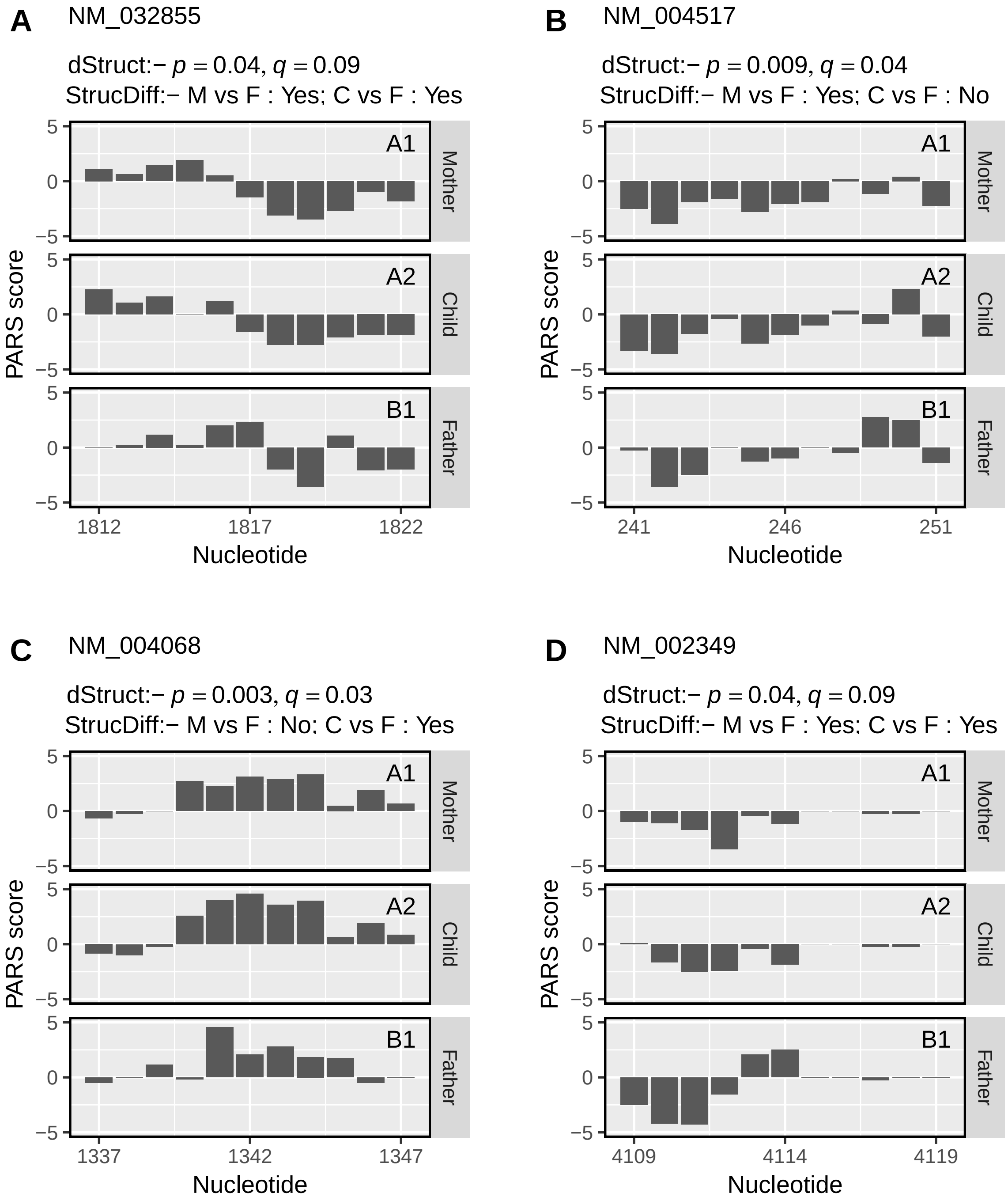

Fig. S7: **Regions found to have riboSNitches by dStruct. A-D.** Each panel shows three samples of reactivity profiles for 11 nt flanking a SNV site. The samples correspond to cell lines derived from mother, child and father. Interestingly, for all riboSNitches that dStruct found, mother and child (samples A1 and A2) formed the allelically identical pair. In a comparison with sample from father (B1), these regions were called DRRs by dStruct. Titles provide transcript ID, p-values and q-values from dStruct and results of pairwise comparisons from StrucDiff. A StrucDiff result of "No" means that the samples compared do not represent a DRR. On the other hand, "Yes" means that the samples are DRRs. Also note that, NM_004517 shares the region in panel **B** with NM_001014794 and NM_001014795. Similarly, NM_004068 shares the region in panel **C** with NM_001025205, and NM_002349 shares the region in panel **D** with transcripts NM_001198759 and NM_001198760.

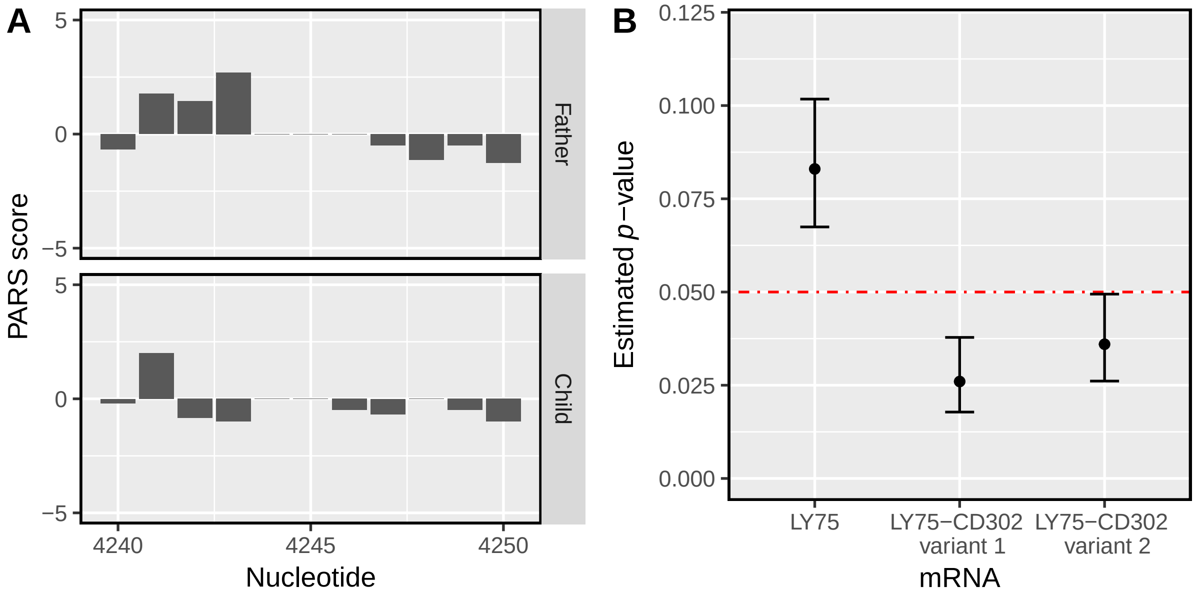

Fig. S8: **StrucDiff results are confounded by data quality. A.** Reactivity profiles for LY75 gene around site 4245, where father and child have different alleles. **B.** The pair of profiles shown in panel **A** was compared by StrucDiff in context of three transcription products – LY75 gene product, variants 1 and 2 of LY75-CD302 fusion product. Depending on the context, StrucDiff assessed different significance levels from the same comparison. Dots indicate the estimated p-values by StrucDiff and error bars represent binomial confidence interval around the estimate, obtained using Wilson method. Dashed red line shows the significance level set by StrucDiff.

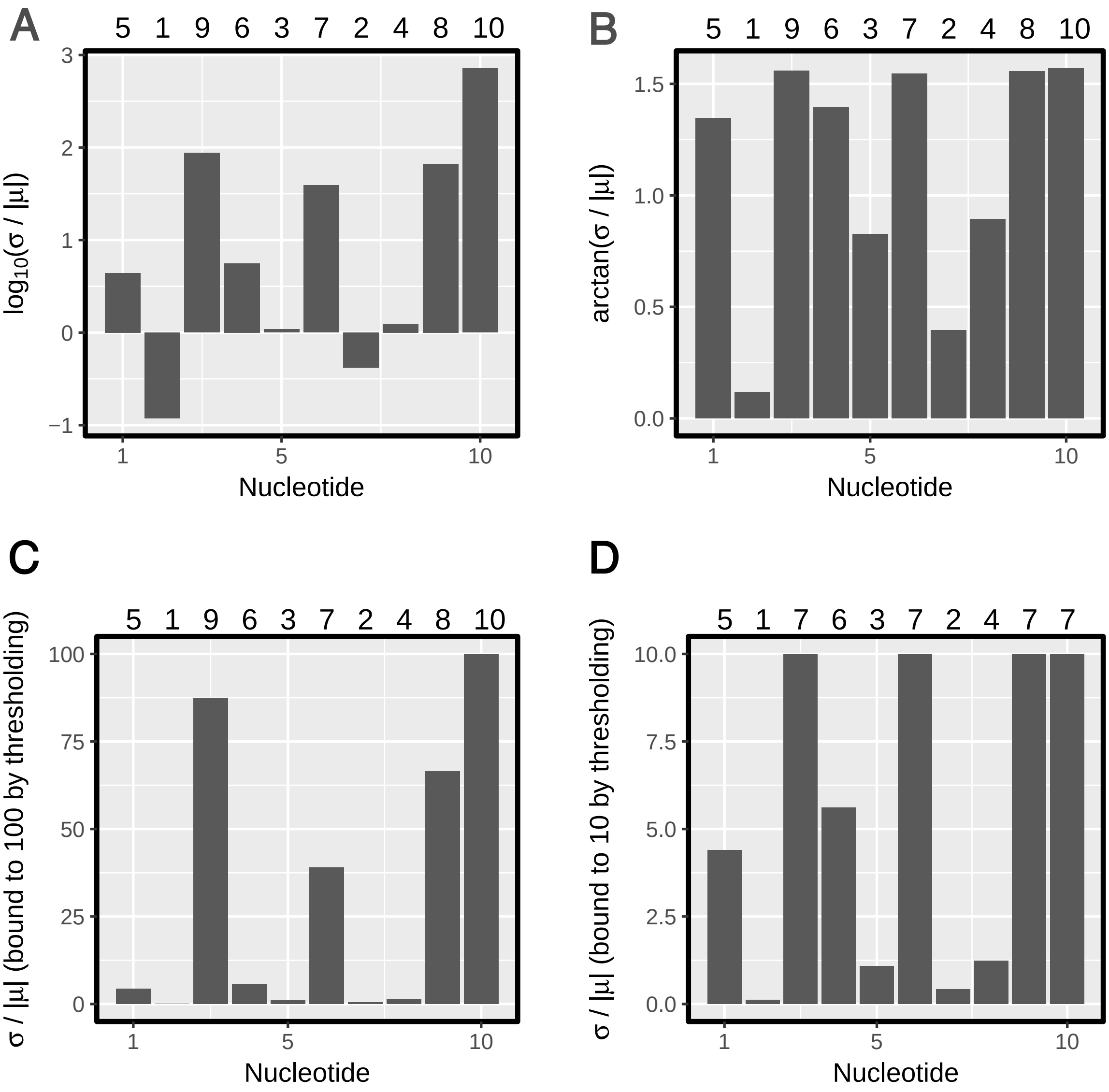

Fig. S9: **All monotonic transformations result in the same relative ranks.** Plotted are d scores for comparison of A1, B1 samples of Figure 1A. We considered four approaches to reduce the impact of outliers on d scores. Each panel shows results from a different approach. **A.** log transformation, **B.** arctan transformation, **C.** thresholding to 100, and **D.** thresholding to 10. Note that the absolute values resulting from each method are different. The numbers outside the top edge of each plot shows the rank of each value in the region. For example, nucleotide 2 has the lowest value in all panels and hence, its rank is 1. It is clear that even though the absolute values resulting from different approaches are different, the ranks of nucleotides are unaltered. This is because all the approaches that we considered are monotonic transformations of raw values. It is noteworthy that thresholding the raw values to a low level might obscure the differences in ranks of nucleotides. For example, in panel D, we used a low threshold of 10, which obscured the difference in rank between nucleotides 3, 6, 9 and 10.

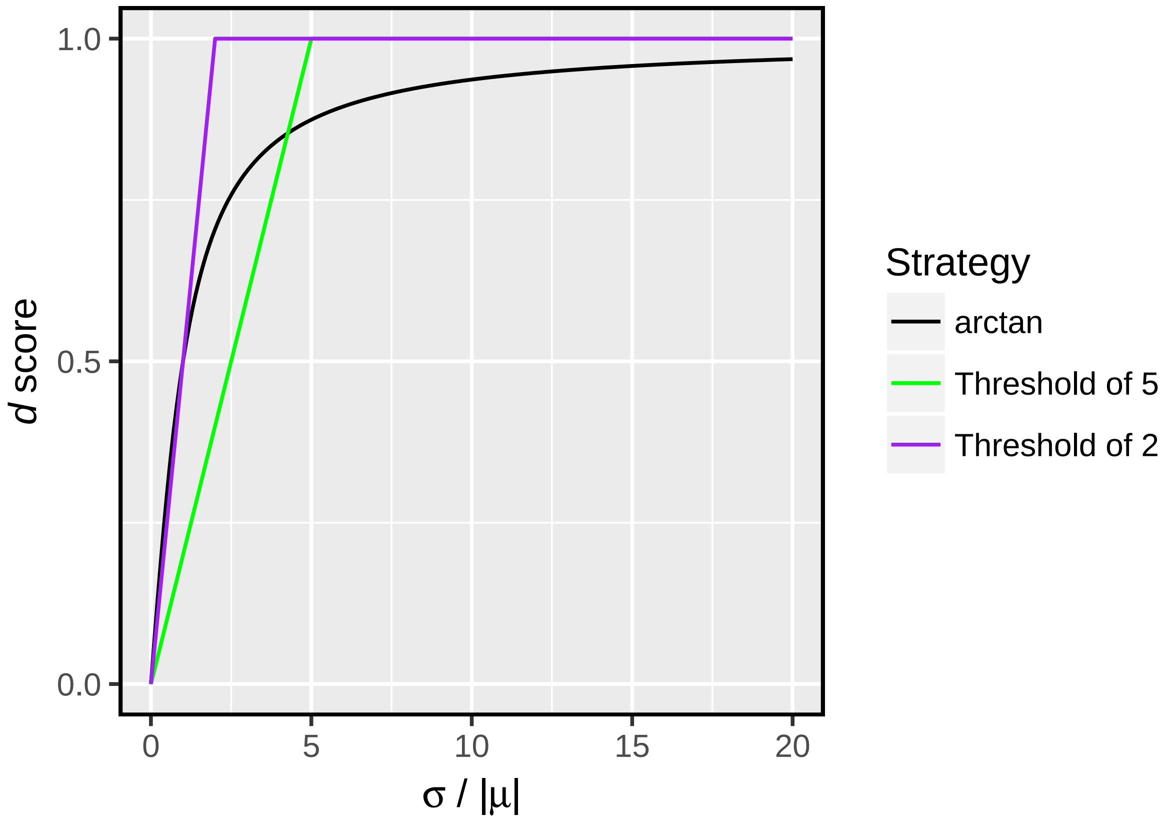

Fig. S10: **Arctan transformation.** We examined the relationship between d scores and σ/μ for the arctan (black line) and thresholding approaches (purple and green lines) to bound d score to 1. The curves reveal a desirable property of arctan transformation. Specifically, while threshold based approaches club all values greater than the threshold to the same level, arctan maps each value of σ/μ uniquely to a value in the range [0,1]. Unique mapping means that all values of σ/μ could be resolved, even if we restricted the range to [0,1]. Since arctan-transformed values asymptotically approach 1 as σ/μ approaches infinity, d scores of 0 and 1 correspond to the extreme cases of coincident and anti-parallel profiles (if two samples have equal reactivities at each nucleotide, we call them coincident; if one sample is exactly inverted version of another, we call them anti-parallel).

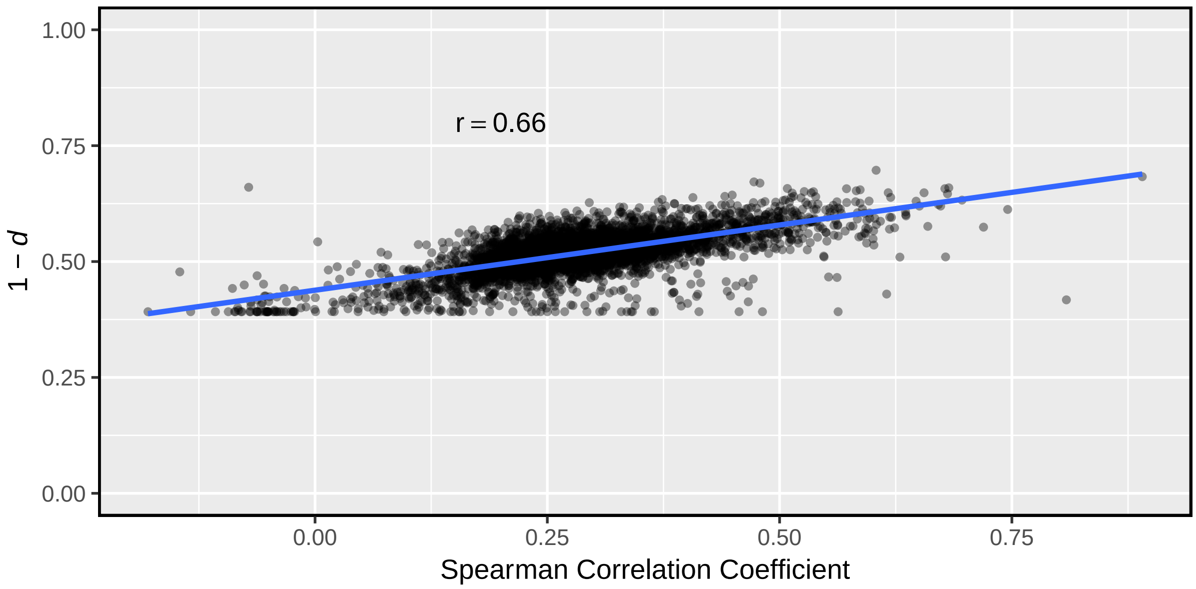

Fig. S11: ***d* scores are related to Spearman correlation coefficient.** Let us say average d score of a transcript is d. Then, $1 - d$ summarizes the degree of coincidence among its replicates. We have plotted $1 - d$ and Spearman correlation coefficients for a pair of replicates of all transcripts in our Structure-Seq data (see Methods). The plot reveals that $1 - d$ is related to Spearman correlation coefficient. The blue line shows a linear fit to the data points.

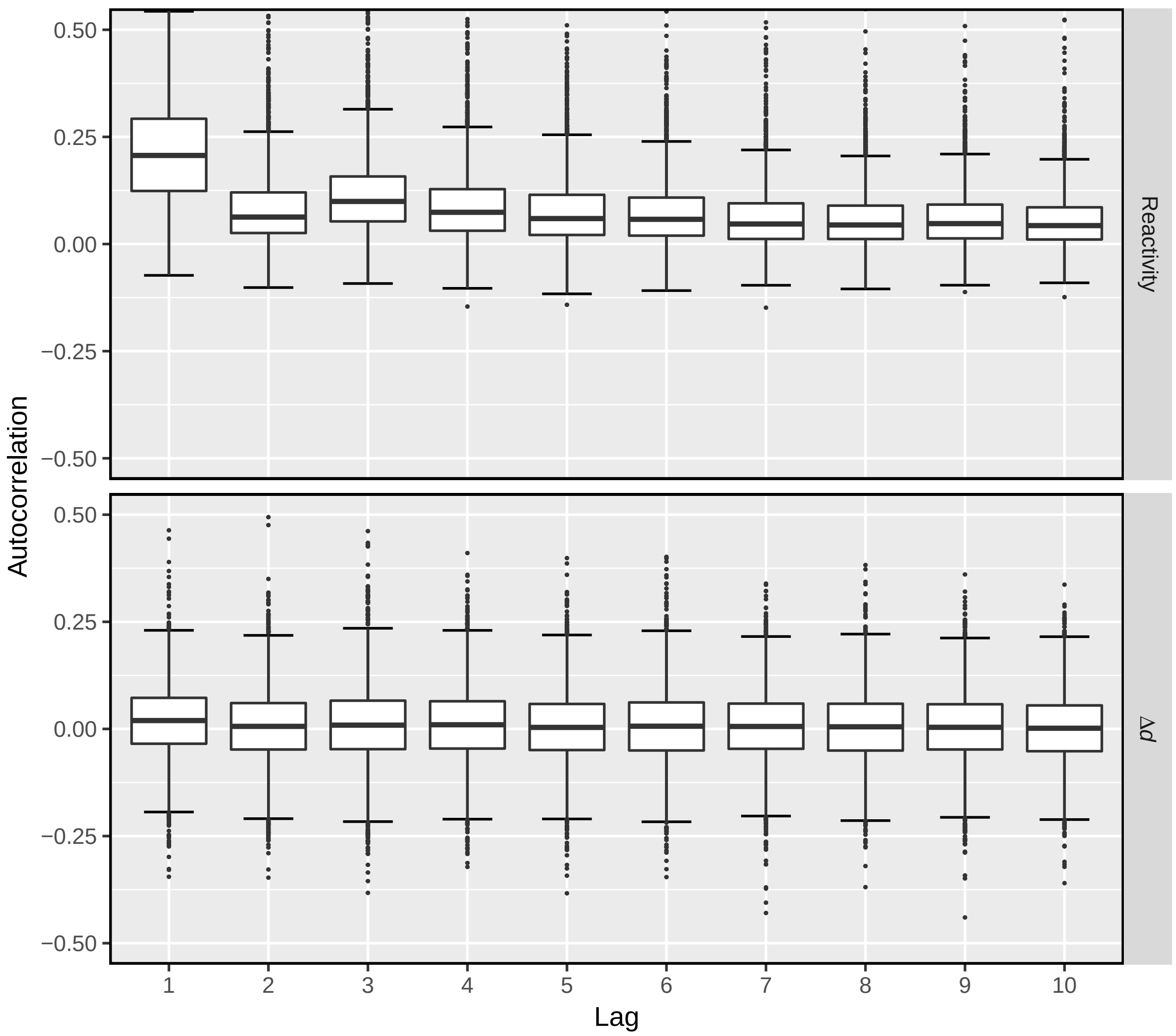

Fig. S12: **Differences of $d_{\mathbf{between}}$ and $d_{\mathbf{within}}$ for nearby nucleotides are uncorrelated.** We obtained Structure-Seq data for three identical replicate samples of *S. cerevisiae* (Supplementary Table S2). For the mRNAs captured in the data with average local coverage > 25, we computed autocorrelation in mean reactivity profiles for lags of 1 to 10 (top panel). Tukey boxplots for distributions of autocorrelations across mRNAs are plotted for different lags. Mean reactivity profiles exhibited correlation between nearby nucleotides. For lag of 1, the median autocorrelation in reactivities was 0.2. Similarly, we computed autocorrelation in Δd profiles (bottom panel). Δd profiles were obtained by randomly assigning one of the samples for each RNA to group A and the other two samples to group B (see Methods in main text). Since all samples were obtained identically, these profiles should be representative of null hypothesis. Δd profiles did not exhibit autocorrelation. For lag of 1, the median autocorrelation in Δd was 0.02. Note that there is a continuous distribution of autocorrelations, which is high for some RNAs. This may be due to miscellaneous sources of noise and/or batch effects. Besides this, a detail about autocorrelation assessments is noteworthy. Since Structure-Seq utilizes base-selective DMS probing, mean reactivity and Δd values were missing for Gs and Us. Each assessment of autocorrelation was done using pairwise complete observations in original and shifted copies of profiles. There were at least ∼30 pairwise complete observations for each assessment. The median number of pairwise complete observations for assessments of autocorrelations in mean reactivity profiles was ∼400 and for Δd profiles, it was ∼150.

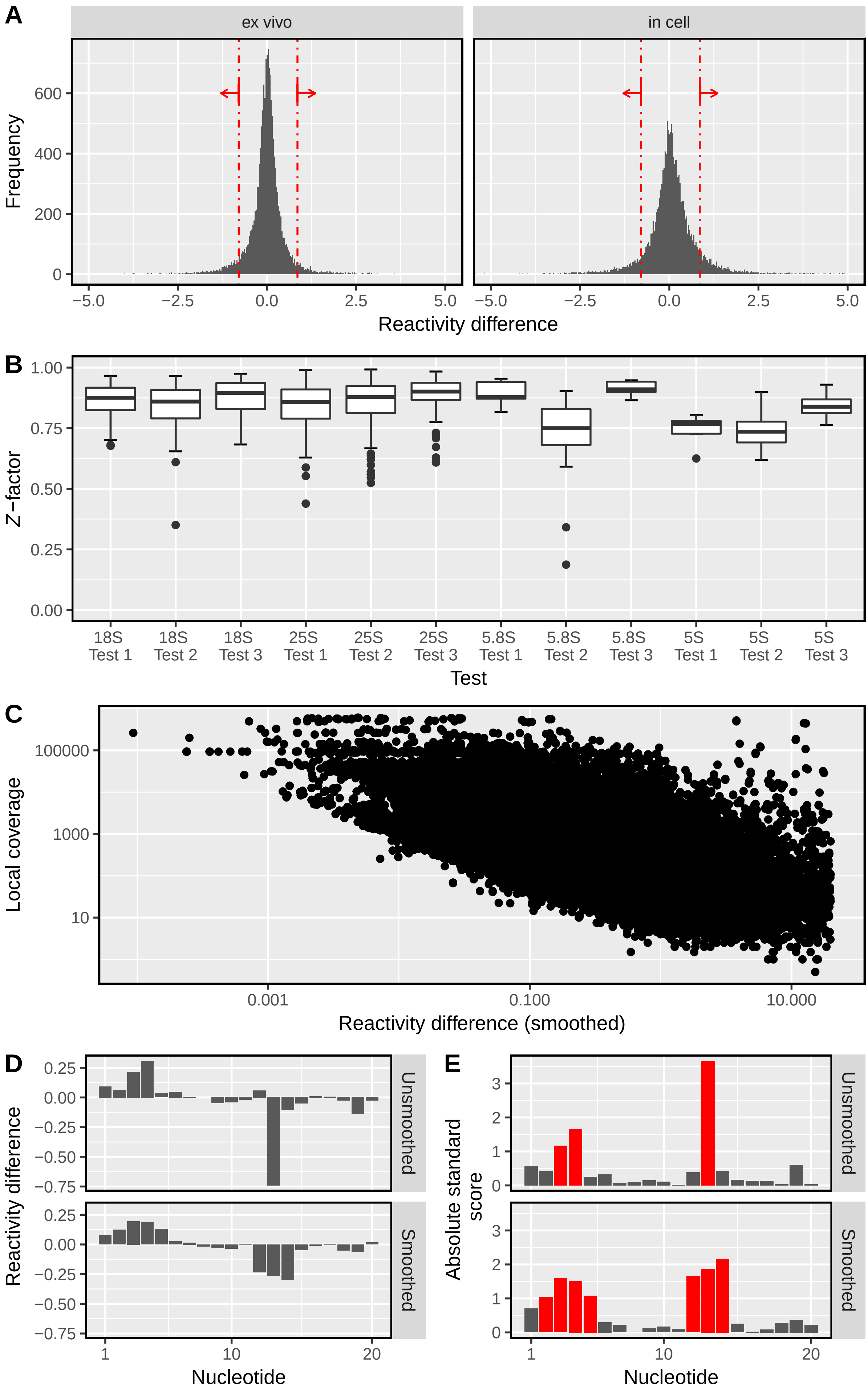
A
ex vivo
in cell
Frequency
Reactivity difference
B
Z−factor
18S Test 1
18S Test 2
18S Test 3
25S Test 1
25S Test 2
25S Test 3
5.8S Test 1
5.8S Test 2
5.8S Test 3
5S Test 1
5S Test 2
5S Test 3
Test
C
Local coverage
Reactivity difference (smoothed)
D
Reactivity difference
Unsmoothed
Smoothed
Nucleotide
E
Absolute standard score
Unsmoothed
Smoothed
Nucleotide

Fig. S13: **Factors leading to high false discovery rate of deltaSHAPE. A.** Distribution of difference of reactivities from pairwise comparisons of two replicate samples of *ex vivo* (left) and in cell (right) probing of *Xist* lncRNA. The arrows indicate direction of increasing absolute value of standard score. Vertical dotted red lines highlight where this value is 1. Note that for any distribution, some data points will fall outside vertical lines, i.e., will have absolute standard score > 1. **B.** Distribution of Z-factor values for the nucleotides with absolute standard score greater than 1 in 12 tests that we performed on highly covered *S. cerevisiae* rRNAs. Note that the requirement for Z-factor to be greater than zero was satisfied always if absolute standard score > 1 for this test dataset. Indeed, Z-factor is largely dependent on coverage. This criterion is satisfied given enough coverage. **C.** Local coverages of nucleotides that satisfied the Z-factor criterion plotted against the corresponding smoothed reactivity differences. The data corresponds to the Structure-Seq data (including simulated DRRs) for the set of 4681 *S. cerevisiae* mRNAs used in simulations. As expected, when reactivity difference is very small (order 10^{-3} or less), large local coverage (order 10^5) is required to satisfy Z-factor criterion. The required local coverage decreases with increase in reactivity difference and local coverage $\sim$10 might be enough to satisfy the criterion for reactivity difference of 0.1 or more. In good quality experiments, local coverage of 10 could be achieved for majority of transcripts. For example, our Structure-Seq data has $\sim$80% of transcripts with greater than 10 local coverage on average. **D.** Difference in reactivities for two samples, unsmoothed data and data after smoothing (for the purpose of illustration, we chose reactivity profiles averaged across replicates for nucleotides 36-55 of *crcB* fluoride riboswitch in absence and presence of fluoride, see Results in main text and Supplementary Table S2). deltaSHAPE requires that at least a few nucleotides screened by absolute standard score and Z-factor be colocalized in a short distance for them to represent a DRR. In simple terms, this is checking if change in reactivities is spread over a region. However, data smoothing (as performed by deltaSHAPE) artificially spreads the signal over a region. Hence, if a region is found, it could be because of a real DRR or an artifact of data smoothing. **E.** Absolute standard scores for reactivity differences shown in **D**. Red bars highlight sites with absolute standard score greater than 1. Note that on data smoothing, the noise/signal at 1 nucleotide is spread to its neighboring nucleotides.

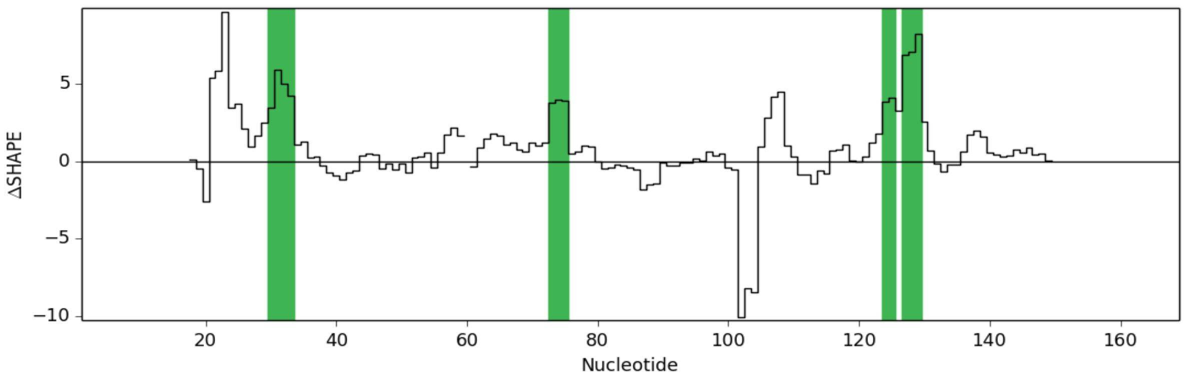

A. deltaSHAPE result on comparing *ex vivo* and in cell profiles of U1 snRNA obtained by SHAPE-MaP [4].

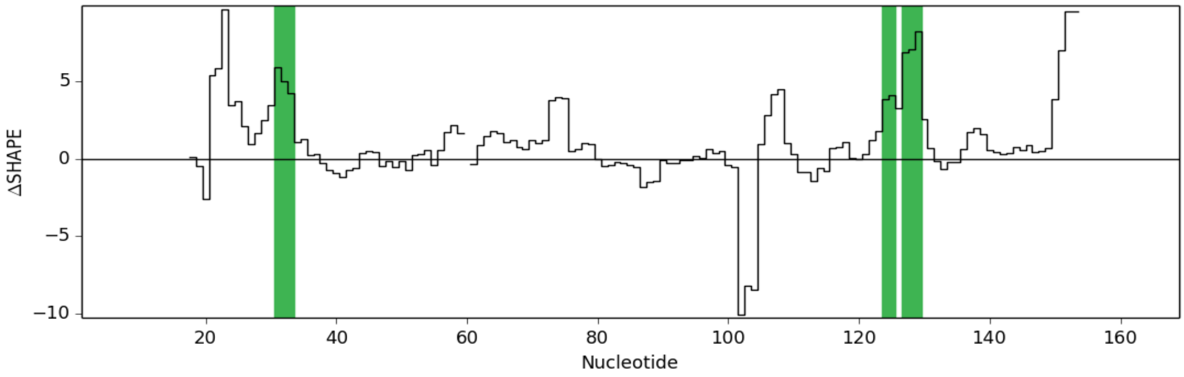

B. deltaSHAPE result on comparing profiles altered by addition of three outliers with $|S_i| \geq 1$ but $Z < 0$.

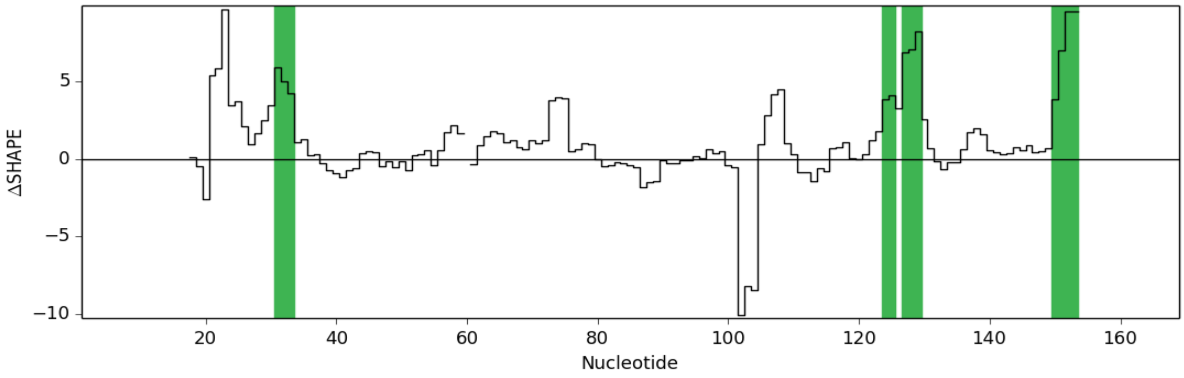

C. deltaSHAPE result on comparing profiles altered by addition of three outliers with $|S_i| \geq 1$ and $Z > 0$.

Fig. S14: **deltaSHAPE is sensitive to outliers.** The plots are obtained as direct outputs from deltaSHAPE. $\Delta\bar{r}_i$ (denoted as ΔSHAPE_i by deltaSHAPE developers) is plotted along Y-axis with X-axis indicating the nucleotide indices, i. DRRs are highlighted with green backgrounds. Rightmost DRR in panel **C** is the region containing outliers.

Table S1: **PCR primers and oligonucleotides used for Structure-Seq.** Barcodes for multiple libraries are highlighted in red letters.

Random-hex RT-primer	5'-CAGACGTGTGCTCTTCCGATCTNNNNNN-3'
ssDNA linker	5'-/5Phos/NNNAGATCGGAAGAGCG TCGTGTAG/3SpC3/-3'
Illumina TruSeq forward primer	5'-AATGATACGGCGACCACCGAGATCTAC ACTCTTTCCCTACACGACGCTCTTCCGATCT-3'
Illumina TruSeq reverse primer_index 1	5'-CAAGCAGAAGACGGCATACGAGATTGGTCAG TGACTGGAGTTCAGACGTGTGCTCTTCCGATCT-3'
Illumina TruSeq reverse primer_index 2	5'-CAAGCAGAAGACGGCATACGAGATGATCTGG TGACTGGAGTTCAGACGTGTGCTCTTCCGATCT-3'
Illumina TruSeq reverse primer_index 3	5'-CAAGCAGAAGACGGCATACGAGATCGTGATG TGACTGGAGTTCAGACGTGTGCTCTTCCGATCT-3'

Table S2: **Download links for all datasets used.**

Structure probing technology	Organism/RNA	Weblink	Database ID/search query	Format
Structure-Seq	*S. cerevisiae* /transcriptome	Zenodo [28]	DOI: 10.5281/zenodo.2536501	Counts, coverages and normalized reactivities for all RNAs are available in R data format alongside scripts used in this study (see note below this table)*. Also available is simulated data that was used in conjunction with experimental data for results illustrated in Figure 5 and Fig. S4-S6.
Cotranscriptional SHAPE-Seq [29]	*B. cereus*/*crcB* fluoride riboswitch	RMDB	FLUORSW_BZCN_0021, FLUORSW_BZCN_0024, FLUORSW_BZCN_0027, FLUORSW_BZCN_0028, FLUORSW_BZCN_0029, FLUORSW_BZCN_0030, FLUORSW_BZCN_0031, FLUORSW_BZCN_0032	.rdat file format
SHAPE-Seq [27]	HIV-1/Rev-response element	Zenodo [28]	Available with scripts used in this manuscript [28]	Excel file
SHAPE-MaP [2, 4]	Mouse trophoblast stem cells/*Xist* long non-coding RNA, U1 small nuclear RNA	Weeks lab	Direct download for Xist lncRNA, Direct download for U1 snRNA	.map file format
PARS [30]	Human lymphoblastoid cell lines/transcriptome	GEO	GSE50676 (Relevant samples are GSM1226157, GSM1226158, GSM1226159, GSM1226160, GSM1226161, GSM1226162)	CSV files with counts

*** NOTE:** We used three Structure-Seq samples for *S. cerevisiae*. The coverages, counts, reactivities and sequences for all RNAs are available over Zenodo. These are organized as follows.

1. The experimental information for all rRNAs is stored in RData format in folder titled "Yeast_rRNAs". There is a file named "rRNA_reactivities.RData". It can be loaded in R or in other languages using R interfaces, e.g., RPy2 for Python. The information stored in this file can be used to reproduce all results illustrated in Figure 3.

2. The experimental information for all mRNAs is stored in folder titled "Tran_lab_data". There is a file named "Scer_reactivity.RData". We introduced simulated reactivities and counts in the experimental data. The simulated reactivity profiles are available in "simulated_reactivities.RData" of "Simulations" folder. These were introduced in randomly selected regions, which are stored in a table in "regions.RData" of the "Simulations" folder. The counts and coverages corresponding to simulated reactivities were back-calculated using the script titled "7. Generate_map_sim_reac_files.R", which is also in "Simulations" folder. This script uses information in "Scer_reactivity.RData",

“simulated_reactivities.RData” and “regions.RData” and generates all the counts and coverages corresponding to simulated reactivity profiles. The simulated reactivities, counts and coverages can be used to reproduce all results illustrated in Figure 5.

Note that the process of simulating reactivities requires generating an ensemble of secondary structures using ViennaRNA for each region and also a reactivity profile for each structure in an ensemble using patteRNA. The structures reported by ViennaRNA and their reactivities are stored in text files, which are compressed together in a file named “sequence_and_RSS_of_selected_regions.zip”. This file is also available in “Simulations” folder.
